# A dimensionless group model of the gas–oil interface stability for CO_2_ gas cap flooding and storage in fault block reservoirs

**DOI:** 10.1038/s41598-025-21110-6

**Published:** 2025-10-24

**Authors:** Gang Hu, Xiangyi Yi, Xuanhua Tian, Rui Wang, Pengchun Li, Linxiong Peng

**Affiliations:** 1 Energy Resource School (State Key Laboratory of Oil and Gas Reservoir Geology and Exploitation), Chengdu University of Technology, Chengdu, China; 2 School of Petroleum Engineering (Engineering Technology Research Center of Guangdong Province for Unconventional Energy), Guangdong University of Petrochemical Technology, Maoming, China; 3 China Research Institute of Petroleum Exploration and Development of Shengli Oilfield, Sinopec Corp., Dongying, China; 4 CAS Key Laboratory of Ocean and Marginal Sea Geology, South China Sea Institute of Oceanology, Chinese Academy of Sciences, Guangzhou, China

**Keywords:** Artificial CO_2_ gas cap immiscible rigid stable gas flooding, Gas–oil interface, Stable gas flooding mechanism, Dimensionless assessment model, Enhanced oil recovery, CO_2_ storage potential, Climate sciences, Energy science and technology

## Abstract

The stability of the gas displacing oil front (i.e., gas–oil interface) is of the utmost importance for the success of the immiscible gas flooding project under crestal gas injection. However, the preceding gas flooding assessment models are deficient in their description of the gas flooding mechanism, and they do not take into account the critical influencing factors in a comprehensive manner. Utilizing theoretical derivation, oilfield justifications, criterion and experiment validation, and dimensional analysis on crestal gas injection for stable flooding, this study presents an innovative theory and technique for artificial CO_2_ gas cap immiscible rigid stable gas flooding under CO_2_ injection, which could not only greatly improve crude oil recovery but also realize CO_2_ geological storage on a large scale, and new insights into displacement mechanism on the gas–oil interface through artificial CO_2_ gas cap immiscible rigid stable gas flooding process. Based on the multiphase filtrate theory, considering the influencing factors such as crude oil density, crude oil viscosity, density of injected gas, gas injection rate, strata dip, liquid phase relative permeability, air permeability in formation direction, viscosity of injected gas, gas phase relative permeability and the acting forces such as buoyancy, gravity, driving pressure, capillary pressure, viscous force and additional resistance in multiphase flow during the artificial CO_2_ gas cap immiscible rigid stable gas flooding process under CO_2_ injection, A simple quantified artificial CO_2_ gas cap immiscible rigid stable gas flooding assessment model (${N_{{\text{GOI}}}}$) was established. The results indicate the artificial CO_2_ gas cap immiscible rigid stable gas flooding process has the theory and field feasibility of greatly enhancing crude oil recovery and realizing CO_2_ geological storage on a large scale. And the oil reservoirs with strata dip, which have large oil and gas density difference, small oil and gas viscosity ratio, large oil and gas relative permeability ratio, large strata dip, and large air permeability in the direction are easy to exert gravity and buoyancy, reduce the influence of capillary pressure, viscosity and additional resistance, benefit to maintain the stability of gas displacing oil front and improve microscopic oil displacement efficiency, and facilitate the implementation of artificial CO_2_ gas cap immiscible rigid stable gas flooding development. In addition, the theoretical deduction, field and experimental validation indicate that artificial CO_2_ gas cap immiscible rigid stable gas flooding under CO_2_ injection can be realized when ${N_{{\text{GOI}}}}$ is greater than 1. The proposed ${N_{{\text{GOI}}}}$ model can be used as a creterion to assess the stablity and efficiency of the crestal gas injection for stable flooding such as artificial CO_2_ gas cap immiscible rigid stable gas flooding, artificial CO_2_ gas cap immiscible stable gas flooding, GAGD, gravity assisted gas injection, and crestal gas injection for stable gravity flooding for theoretical investigation, numerical simulation, laboratory test and field trial project design or operation.

## Introduction

Carbon dioxide (CO_2_) capture, utilisation and storage (CCUS) is regarded as a pivotal approach to curtailing greenhouse gas emissions. The technology of injecting CO_2_ into oil reservoirs to flood crude oil and thus enhance oil recovery (CO_2_-EOR), if it utilises industrial emission CO_2_ captured by human beings or CO_2_ captured from the atmosphere, has the potential to not only increase the production of crude oil, as well as facilitating large-scale underground long-term storage of CO_2_, thereby reducing the direct emission of CO_2_ or lowering the concentration of CO_2_ in the air, which is the preferred option for CCUS at present. Nevertheless, there are still many challenges in the application of CO_2_-EOR in oilfields, especially in complex fault block reservoirs.

The complex fault block reservoirs, which are distributed extensively across central and eastern China, are characterised by a number of distinct features. These include a substantial stratigraphic dip, a relatively confined oil-bearing area, effective closure ability, a complex fracture structure, and a substantial oil layer. In addition, some of these reservoirs exhibit the distinctive characteristics of an original angry top and a side bottom water. Following the implementation of both natural energy and water drive development in this reservoir category, the inefficiency of extracting residual oil from the reservoir’s central and upper regions has led to the accumulation of flow-around oil within the water-flooded area and the formation of attic oil in the higher parts. Statistical analysis indicates that both bypass (i.e., flow-around) and attic oil are the predominant residual oil types in inclined block reservoirs following water drive development. The challenges associated with the optimisation of the injection and extraction well network, in conjunction with the constraints imposed by the reservoir boundary, frequently impede the effective implementation of the water-driven development method for attic oil in inclined fault block reservoirs. Historically, horizontal well technology was employed as a highly effective method to extract residual oil and enhance recovery in such reservoirs. However, the substantial expense and the complexity of its implementation hindered its widespread promotion.

The SINOPEC Shengli Oilfield E&P Research Institute has confirmed that the crestal gas injection flooding technology is a more effective method for the recovery of attic oil and flow-around oil in such reservoirs. In 2010, the artificial N_2_ gas cap immiscible flooding technology was proposed. Recent studies and project practices have concluded that artificial gas cap flooding is one of the more effective top gas injection technologies for restoring formation energy and enhancing oil recovery after the development of water flooding development in the above inclined block reservoirs. It has been demonstrated that artificial gas cap flooding overcomes the limitations of low displacement efficiency and premature gas channeling caused by gas viscous fingering instabilities and gravity overlap effect during gas flooding such as continuous gas injection, water alternating gas and gas assisted gravity drainage. Therefore, it can significantly improve the oil displacement efficiency and sweep efficiency of the oil reservoirs. Consequently, the final oil recovery of the artificial gas cap flooding is the highest among all among all immiscible flooding technologies. However, the development effect of artificial gas cap flooding is contingent on the stability of the gas displacing oil front (i.e., gas–oil interface). Theoretically, the stability of the gas–oil interface is not only a prerequisite for the efficient production of attic oil and bypass oil, but also a key factor for the success of the artificial gas cap immiscible stable flooding process.

As demonstrated and reviewed in Table 1, a number of group models and critical rate models have been proposed for the purpose of describing the crestal gas injection for stable flooding mechanism and evaluating the stability of the gas–oil interface. The group models are a set of hydrodynamics dimensionless indexes that include: the Reynolds index, Schmid index, Weber index, Froude index, Lewis index, Grashof index, Leverett index, Darcy index, and the Gravity indexes, the Capillary index, and the Bond index., Dombrowski–Brownell index, Diffusion index, Dispersion index, Pecklet’s index, and Dissolution index. Furthermore, the critical rate models encompass six distinct forms, namely the Hill Critical Injection Rate, the Dietz Critical Injection Rate, the Dumore Critical Injection Rate, the Rutherford Critical Injection Rate, the Slobod and Howlett Critical Rate, and the Wang Critical Rate.

**Table 1:** Major achievements and defects in the dimensionless group and critical injection rate models.

No	Resources	Models	Formulas	Major achievements	Major defects	Adaptability
1	Geertsma, et al.^1^	Reynolds number (${\text{Re}}$)	${\text{Re}} = \frac{v\rho l}{\mu }$	The ratio of the inertial force to the viscous force of a fluid	Ignores the effects of capillary force, buoyancy, gravity, contact angle, strata dip, etc	Cold-water drive
2	Geertsma, et al.^1^	Peclet number (${\text{Pe}}$)	$Pe = \frac{\lambda }{\rho cvl}$	The ratio of convection to diffusion	Ignores the effects of inertial force, viscous force, capillary force, buoyancy, contact angle, strata dip, etc	Hot-water drive
3	Geertsma, et al.^1^	Schmid number (${\text{Sc}}$)	$Sc = \frac{vD}{\mu }$	The product of the diffusion group ($\frac{D}{vl}$) and the Reynolds number	Ignores the effects of capillary force, buoyancy, gravity, contact angle, strata dip, etc	Solvent inject ion, cold-water drive
4	Geertsma, et al.^1^	Weber number (${\text{We}}$)	$We = \frac{{v^{2} \rho l}}{\sigma \cos \theta }$	The product of the ratio of viscous force to capillary force and the Reynolds number	Ignores the effects of buoyancy, gravity, strata dip, etc	Cold-water drive
5	Geertsma, et al.^1^	Froude number (${\text{Fr}}$)	$Fr = \frac{{v^{2} }}{gl}$	The product of the ratio of gravity to viscous force and the Reynolds number	Ignores the effects of capillary force, buoyancy, contact angle, strata dip, etc	Cold-water drive
6	Geertsma, et al.^1^	Prandtl number (${\text{Pr}}$)	$Pr = \frac{\lambda }{c\mu }$	The product of the Peclet number and the Reynolds number	Ignores the effects of capillary force, buoyancy, gravity, contact angle, strata dip, etc	Hot-water drive
7	Geertsma, et al.^1^	Lewis number (${\text{Le}}$)	$Le = \frac{\lambda }{\rho cD}$	The product of the diffusion group ($\frac{D}{vl}$) and the Peclet number	Ignores the effects of inertial force, viscous force, capillary force, buoyancy, contact angle, strata dip, etc	Hot-water drive
8	Geertsma, et al.^1^	Grashof number (${\text{Gr}}$)	$Gr = \frac{{\rho^{2} gl^{3} \beta T}}{{\mu^{2} }}$	The product of the state group ($\beta T$) and the Froude number	Ignores the effects of capillary force, buoyancy, contact angle, strata dip, etc	Hot-water drive
9	Kulkarni^2^, Kulkarni and Rao^3^	Index of productivity ($I.P.$)	$I.P. = \frac{Enhanced\,Production (Bbl/D)}{{Flood\;Volume (Ac - ft)}}$	The ratio of the enhanced production to the flood volume	Only be identified ex post factor	Unlimited
10	Shook, et al.^4^, Sharma and Rao^5^	Gravity number ($N_{{\text{G}}}$)	$N_{{\text{G}}} = \frac{{\Delta \rho g\left( {\frac{K}{\phi }} \right)}}{{\mu_{{\text{o}}} v_{{\text{d}}} }}$	The ratio of the Bond number to the Capillary number, or the ratio of gravity to viscous force	Ignores the effects of inertial force, capillary force, contact angle, strata dip, relative permeability, etc	Gas -assisted Gravity drainage processes
11	Kulkarni^2^, Kulkarni and Rao^3^	Gravity number ($N_{{\text{G}}}$)	$N_{{\text{G}}} = \frac{\Delta \rho gk}{{\Delta \mu v}}$	The ratio of the gravity, buoyancy to the viscous forces	Ignores the effects of inertial force, capillary force, contact angle, strata dip, relative permeability, etc	GAGD process, gravity drainage processes
12	Wu, et al.^6^	Gravity number ($N_{{\text{G}}}$)	$N_{{\text{G}}} = \frac{{\Delta \rho g\left( {\frac{K}{\phi }} \right)}}{{\mu_{{\text{g}}} v_{{\text{g}}} }}$	The ratio of the Bond number to the Capillary number, or the ratio of gravity to viscous force	Ignores the effects of inertial force, capillary force, contact angle, strata dip, relative permeability, etc	Gas-assisted gravity drainage processes
13	Wu, et al.^6^	Modified gravity number ($N^{\prime}_{{\text{G}}}$)	$N^{\prime}_{{\text{G}}} = \frac{{\Delta \rho g\left( {\frac{K}{\phi }} \right)}}{{\mu_{{\text{g}}} v_{{\text{g}}} }}\left( {1 - \cos {\uptheta }} \right)$	The ratio of the Capillary number to the Bond number, which considers the influence of reservoir wettability on the recovery factor	Ignores the effects of inertial force, capillary force, contact angle, strata dip, relative permeability, buoyancy, etc	gravity drainage processes
14	Shook, et al.^4^	Gravity drainage number ($N_{{{\text{GD}}}}$)	$N_{{{\text{GD}}}} = N_{{\text{G}}} + \left( {\frac{{\rho_{{\text{g}}} }}{{\rho_{{\text{o}}} }}\left( {N_{{\text{C}}} + N_{{\text{B}}} } \right)} \right)$	An immiscible gravity number contains gravity number, Bond number and Capillary number^3^. The literate review suggested that two ratios: density and viscosity (gas to oil), were pivotal for gravity drainage flow. Therefore, the density ratio (displacing to displaced phase) was factored	Ignores the effects of inertial force, buoyancy, contact angle, strata dip, etc	Gravity drainage processes, but excellent applicability was obtained for immiscible floods
15	Wu, et al.^6^	Gravity drainage number ($N_{{{\text{GD}}}}$)	$N_{{{\text{GD}}}} = N_{{\text{G}}} + \left( {\frac{{\rho_{{\text{g}}} }}{{\rho_{{\text{o}}} }}\left( {N_{{\text{C}}} + N_{{\text{B}}} } \right)} \right)$	An immiscible gravity number contains gravity number^6^, Bond number^3^ and Capillary number^3^. The literate review suggested that two ratios: density and viscosity (gas to oil), were pivotal for gravity drainage flow. Therefore, the density ratio (displacing to displaced phase) was factored	Ignores the effects of inertial force, buoyancy, capillary force, contact angle, strata dip, relative permeability, etc	Gravity drainage processes, but excellent applicability was obtained for immiscible floods
16	Wu, et al.^6^	Modified gravity drainage number ($N^{\prime}_{{{\text{GD}}}}$)	$N^{\prime}_{{{\text{GD}}}} = N^{\prime}_{{\text{G}}} \left( {1 + \frac{{\mu_{{\text{g}}} }}{{\mu_{{\text{o}}} }}} \right) + \frac{{\rho_{{\text{g}}} }}{{\rho_{{\text{o}}} }}\left( {N_{{\text{C,1}}} + N_{{\text{B}}} } \right)$	An immiscible gravity number contains modified gravity number^3^, Bond number^5^ and Capillary number^7^. The viscosity ratio and density ratio (displacing to displaced phase) were factored	Ignores the effects of inertial force, buoyancy, strata dip, etc	Gravity drainage processes, but excellent applicability was obtained for immiscible floods
17	Yang, et al. ^8^	Yang gravity number ($N_{{{\text{G}}{\text{Y}}}}$)	$N_{{{\text{G}}{\text{ Y}}}} = \frac{{KK_{{{\text{ro}}}} \Delta \rho_{o,g} g\sin \alpha }}{{v_{{\text{o}}} \mu_{{\text{o}}} }}$	The ratio of the gravity to the viscous force	Ignores the effects of buoyancy and capillary force of gas	Crestal gas injection for stable flooding processes
18	Ren, et al.^9^	Gravity assisted gas injection number ($N_{{{\text{GAGI}}}}$)	$N_{{{\text{GAGI}}}} = \frac{{kg\Delta \rho_{o,g} \sin \alpha }}{{V\mu_{{\text{o}}} }}$	Based on Darcy’s law and the potential energy relationship, the ratio of the product of the difference between gravity and buoyancy, permeability and the sine of the strata dip, to the product of the viscous force and the injection rate	Ignores the effects of gas viscous force, buoyancy, contact angle, relative permeability, etc	Gravity assisted gas injection
19	Chatzis and Morrow^7^, Morrow, et al.^10^, Grattoni, et al.^11^, Sharma and Rao^5^	Capillary number ($N_{{\text{C,1}}}$)	$N_{{\text{C,1}}} = \frac{v\mu }{\sigma }$	The ratio of viscous to capillary forces	Ignores the effects of inertial force, gravity, buoyancy, contact angle, strata dip, etc	Gravity drainage processes
20	Chatzis and Morrow^7^, Grattoni, et al.^11^	Capillary number ($N_{{\text{C,2}}}$)	$N_{{\text{C,2}}} = \frac{{k_{{\text{w}}} \Delta P}}{L\sigma }$	The ratio of viscous to capillary forces	Ignores the effects of inertial force, gravity, buoyancy, contact angle, strata dip, etc	Gravity drainage processes
21	Chatzis and Morrow^7^, Grattoni, et al.^11^	Capillary number ($N_{{\text{C,3}}}$)	$N_{{\text{C,3}}} = \frac{{k_{{\text{a}}} \Delta P}}{L\sigma }$	The ratio of viscous to capillary forces	Ignores the effects of inertial force, gravity, buoyancy, contact angle, strata dip, etc	Gravity drainage processes
22	Grattoni, et al.^11^	Three-phase Capillary number ($N_{{\text{C}}}$)	$N_{{\text{C}}} = \frac{{2v_{{\text{g}}} \mu_{g} }}{{P{\text{c}}_{{\text{i}}} R_{{\text{a}}} }}$	The ratio of viscous to capillary forces The specific density difference ($\Delta \rho_{{\text{i}}}$) in capillary force ($P{\text{c}}_{{\text{i}}}$) is defined for each distinct wettability and initial condition	Ignores the effects of inertial force, viscous force, contact angle, strata dip, etc	Three-phases gravity drainage processes
23	Grattoni, et al.^11^, Kulkarni^2^, Kulkarni and Rao^3^	Capillary number ($N_{{\text{C}}}$)	$N_{{\text{C}}} = \frac{v\mu }{{P_{{\text{C}}} R_{{\text{A}}} }}2\cos \theta$	The ratio of capillary to viscous forces	Ignores the effects of inertial force, gravity, buoyancy, strata dip, etc	GAGD Process, gravity drainage processes
24	Kulkarni^2^	Capillary number ($N_{{{\text{ca}}}}$)	$N_{{{\text{ca}}}} = \frac{v\mu }{{\sigma \cos \theta }}$	The ratio of capillary to viscous forces	Ignores the effects of inertial force, gravity, buoyancy, strata dip, etc	Miscibility gravity drainage processes
25	Yang, et al.^8^	Yang Capillary number ($N_{{\text{C}}}$)	$N_{{\text{C}}} = \frac{{v\mu_{{\text{o}}} H}}{{K_{{{\text{ro}}}} \sqrt {\phi K} \sigma \cos \theta \sin \alpha }}$	The ratio of capillary to viscous forces	Ignores the effects of buoyancy and capillary force of gas	Crestal gas injection for stableflooding processes
26	Yang, et al.^8^	Yang variation Capillary number ($N_{{{\text{Cam}}}}$)	$N_{{{\text{Cam}}}} = \frac{{v\mu_{{\text{o}}} H}}{{\left( {S_{{\text{o}}} - S_{{{\text{or}}}} } \right)K_{{{\text{ro}}}} \sqrt {\phi K} \sigma \cos \theta \sin \alpha }}$	The ratio of capillary to viscous forces	Ignores the effects of buoyancy and capillary force of gas	Crestal gas injection for stable flooding processes
27	Morrow, et al.^10^	Bond number ($N_{{\text{B}}}$)	$N_{{\text{B}}} = \frac{{\Delta \rho gr^{2} }}{\sigma }$	The ratio of buoyancy to the capillary force, or the ratio of the product of the difference between gravity and buoyancy, and the bead radius squared, to the capillary force	Ignores the effects of inertial force, viscous force, contact angle, strata dip, etc	Gravity drainage processes
28	Hove, et al.^12^	Bond number ($N_{{\text{B}}}$)	$N_{{\text{B}}} = \frac{{\Delta \rho gR^{2} }}{\sigma }$	The ratio of buoyancy to the capillary force, or the product of the difference between gravity and buoyancy, and particle radius squared, to the capillary force	Ignores the effects of inertial force, viscous force, contact angle, strata dip, etc	Gravity drainage processes
29	Hove, et al.^12^	Bond number ($N_{{\text{B}}}$)	$N_{{\text{B}}} = \frac{\Delta \rho grh}{{2\sigma }}$	The ratio of buoyancy to capillary force	Ignores the effects of inertial force, viscous force, contact angle, strata dip, etc	Gravity segregation processes
30	Hove, et al.^12^, Grattoni, et al.^11^	Bond number ($N_{{\text{B}}}$)	$N_{{\text{B}}} = \frac{{\Delta \rho gr_{{{\text{av}}}} h_{{{\text{av}}}} }}{2\sigma }$	The ratio of buoyancy to capillary force $r_{{{\text{av}}}}$ refers to the average pore throat radius of the porous medium, and $h_{{{\text{av}}}}$ refers to the average height of the non-wetting phase ganglia	Ignores the effects of inertial force, viscous force, contact angle, strata dip, etc	Gravity drainage processes, a large number of non-wetting phase ganglia
31	Edwards, et al.^13^, Kulkarni^2^	Macroscopic Bond number ($N_{{\text{B}}}$)	$N_{{\text{B}}} = \frac{\Delta \rho gL}{{P_{{\text{c}}}^{*} }}$	The ratio of gravitational-to-capillary forces, or the product of the difference between gravity and buoyancy, and the ratio of the sample length to the capillary force (i.e. the characteristic capillary force)	Ignores the effects of inertial force, viscous force, contact angle, strata dip, etc	Gravity drainage processes
32	Grattoni, et al.^11^	Bond number ($N_{{\text{B}}}$)	$N_{{\text{B}}} = \frac{{\Delta \rho_{{{\text{og}}}} gZR_{{\text{a}}} }}{{2\sigma_{{_{og} }} }}$	The ratio of buoyancy to capillary force $R_{{\text{a}}}$ refers to the average pore throat radius, and $Z$ refers to average position of the gas interface	Ignores the effects of inertial force, viscous force, contact angle, strata dip, etc	Gravity drainage processes
33	Grattoni, et al.^11^	Three-phase Bond number ($N_{{\text{B}}}$)	$N_{{\text{B}}} = \frac{{\Delta \rho_{{\text{i}}} gZR_{{\text{a}}} }}{{2\sigma_{{\text{i}}} }}$	The ratio of buoyancy to capillary force. The specific density difference ($\Delta \rho_{{\text{i}}}$) in capillary force ($P{\text{c}}_{{\text{i}}}$) is defined for each distinct wettability and initial condition	Ignores the effects of inertial force , viscous force, contact angle, strata dip, etc	Three-phases gravity drainage processes
34	Grattoni, et al.^11^, Kulkarni^2^, Kulkarni and Rao^3^	Macroscopic Bond number ($N_{{\text{B}}}$)	$N_{{\text{B}}} = \frac{{\Delta \rho gl^{2} }}{\sigma }$	The ratio of gravity (or buoyancy in some cases) to capillary force The ratio of the product of the difference between gravity and buoyancy, and the characteristic length of the porous medium (often taken as the average grain radius) squared, to the capillary force	Ignores the effects of inertial force, viscous force, contact angle, strata dip, etc	Gravity drainage processes, For the trapping of the non-wetting phase
35	Grattoni, et al.^11^, Kulkarni^2^, Kulkarni and Rao^3^	Macroscopic Bond number ($N_{{\text{B}}}$)	$N_{{\text{B}}} = \frac{{\Delta \rho gl^{2} }}{{\sigma \sqrt {{\phi \mathord{\left/ {\vphantom {\phi k}} \right. \kern-0pt} k}} }}$	The ratio of the product of the difference between gravity and buoyancy, and the characteristic length (represented by the grain diameter) squared, to the product of the capillary force (i.e. characteristic capillary force) and 2 square root of the ratio of porosity to permeability	Ignores the effects of inertial force, viscous force, contact angle, strata dip, etc	Gravity drainage processes
36	Sharma and Rao^5^, Wu, et al.^6^	Bond number ($N_{{\text{B}}}$)	$N_{{\text{B}}} = \frac{{\Delta \rho g\left( {\frac{k}{\phi }} \right)}}{\sigma }$	The ratio of gravity to capillary force	Ignores the effects of inertial force , viscous force, contact angle, strata dip, etc	Gravity drainage processes, a large number of non-wetting phase ganglia
37	Edwards, et al.^13^, Kulkarni^2^	Dombrowsk–Brownell number or microscopic Bond number ($N_{{{\text{DB}}}}$)	$N_{{{\text{DB}}}} = \frac{\Delta \rho gk}{\sigma }$	The ratio of gravitational-to-capillary forces, or the ratio of the product of the difference between gravity and buoyancy, and phase permeability, to the capillary force	Ignores the effects of inertial force , viscous force, contact angle, strata dip, etc. The relative permeability is to be determined	Gravity drainage processes. Be difficult to use
38	Edwards, et al.^13^	Modified Dombrowsk– Brownell number ($\tilde{N}_{{{\text{DB}}}}$)	$\tilde{N}_{{{\text{DB}}}} = \frac{{\Delta \rho gkk_{r} }}{\sigma }$	The ratio of gravitational-to-capillary forces, or the ratio of the difference between gravity and buoyancy, to the capillary force. The model can be used to describe the phenomenon of increasing the effect of capillarity by capturing the effective permeability, particularly within in the range of low saturation	Ignores the effects of inertial force, viscous force, contact angle, strata dip, etc	Gravity drainage processes
39	Chen, et al.^14^	Chen Bond number ($N^{\prime}_{{\text{b}}}$)	$N^{\prime}_{{\text{b}}} = \frac{{kg\Delta \rho_{o,g} \sin \alpha }}{\sigma }$	The ratio of gravity to capillary force	Ignores the effects of buoyancy of gas	Gravity drainage processes
40	Grattoni, et al.^11^, Kulkarni and Rao^3^	Grattoni number ($N$)	$N = N_{{\text{B}}} + A\left( {\frac{{\mu_{{\text{d}}} }}{{\mu_{{\text{g}}} }}} \right)N_{{\text{C}}}$	A new combined dimensionless group contains Bond number and Capillary number, which measures the combined effect of gravity, buoyancy and the ratio of viscous force(i.e. the viscosity ratio between the displaced phase and the displacing phase) to capillary force. The scaling factor of A is employed to describe the varying strengths of all the forces acting at different stages during a displacement	Ignores the effects of inertial force, contact angle, strata dip, etc	Three-phase gravity drainage flow
41	Rostami, et al.^15^, Rostami, et al.^16^	Rostami number ($N_{{{\text{co}}}}$)	$N_{{{\text{co}}}} = \frac{{N_{{\text{B}}} \times \left( {\frac{{\mu_{{\text{g}}} }}{{\mu_{{\text{o}}} }}} \right)^{0.5} }}{{N_{{\text{C}}}^{0.5} }}$	The ratio of Bond to Capillary number	Ignores the effects of buoyancy, viscous forces and additional resistance of gas, additional resistance of crude oil	Forced gavity dinage processes
42	Yang, et al.^8^	Yang comprehensive number($N_{{\text{a}}}$)	$N_{{\text{a}}} = N_{{\text{G}}} + 17225\left( {\frac{{\rho_{{\text{g}}} }}{{\rho_{{\text{o}}} }}\frac{{\mu_{{\text{o}}} }}{{\mu_{{\text{g}}} }}} \right)N_{{{\text{Cam}}}}$	The sum of gravity number and Yang variation Capillary number	Ignores the effects of buoyancy and capillary force of gas	Crestal gas injection for stable flooding processes
43	Kelkar and Gupta^17^, Li and Liu^18^	Diffusion number/dispersion number ($N_{{{\text{D}}1}}$)	$N_{{{\text{D}}1}} = \frac{L}{H}\sqrt {\frac{{K_{{\text{T}}} }}{{K_{{\text{L}}} }}}$	The ratio of the transverse to the longitudinal physical diffusion coefficient, or the product of the spacing of injection-production Wells and the square root of the ratio of the transverse to the longitudinal physical diffusion coefficient	Ignores the effects of inertial force, capillary force, buoyancy, gravity, contact angle, strata dip, etc	Gas injection unsteady displacement process
44	Rostami, et al.^16^	Dissolution number ($N_{{{\text{dis}}}}$)	$N_{{{\text{dis}}}} = \frac{{N_{{\text{B}}} \times \left( {\frac{{\mu_{{\text{g}}} }}{{\mu_{{\text{o}}} }}} \right)^{2} }}{{N_{{\text{C}}}^{{}} }}$	The ratio of bond to Capillary number	Ignore the effects of buoyancy, viscous forces and additional resistance of gas, additional resistance of crude oil	Forced gavity dinage processes
45	Novakovic^19^	Peclet’s number ($N_{Pe}$)	$N_{Pe} = \frac{\mu l}{{\phi D}}$	The ratio of convective to dispersive transport is calculated under the assumption of stationary boundary conditions, whereby the velocity remains constant	Ignore the effects of inertial force, viscous force, capillary force, buoyancy, contact angle, dip angle, molecular diffusion, etc	Miscible displacement
46	Kelkar and Gupta^17^, Li and Liu^18^	Dispersion number ($N_{{{\text{D}}2}}$)	$N_{{{\text{D}}2}} = \frac{L}{H}\sqrt {\frac{{\alpha _{{\text{T}}} }}{{\alpha _{{\text{L}}} }}}$	The ratio of the transverse to the longitudinal dispersion coefficient, or the product of the spacing of injection-production Wells and the square root of the ratio of the transverse to longitudinal dispersion coefficient	Ignores the effects of inertial force, capillary force, buoyancy, gravity, contact angle, strata dip, molecular diffusion, etc	Gas injection unsteady displacement process
47	Dietz^20^	Dietz critical injection rate ($u_{c}$)	$u_{c} = \frac{{\Delta \rho_{{{\text{og}}}} g\sin \theta }}{{\frac{{\mu_{{\text{o}}} }}{{kk_{{{\text{ro}}}} }} - \frac{{\mu_{{\text{g}}} }}{{kk_{{{\text{rg}}}} }}}}$	The ratio of the product of the pressure difference between gravity, and capillary force, and the sine of the strata dip, to the reciprocal difference between oil and gas mobility	Ignores the effects of buoyancy force, contact angle, etc	Gravity drainage processes. Homogeneous, piston displacement, incompressible rock and fluid
48	Rutherford^21^	Rutherford critical injection rate ($\left( {{q \mathord{\left/ {\vphantom {q A}} \right. \kern-0pt} A}} \right)_{{{\text{st}}}}$)	$\left( {{q \mathord{\left/ {\vphantom {q A}} \right. \kern-0pt} A}} \right)_{{{\text{st}}}} = 0.0439\frac{{kg\left( {\rho_{{\text{o}}} - \rho_{{\text{s}}} } \right)}}{{\mu_{{\text{o}}} - \mu_{{\text{s}}} }}$	In light of the potential for a minor hypothetical protrusion of one of the fluids into the other, the model may be capable of determining the conditions that result in miscible displacement of oil by light hydrocarbon mixtures	Ignores the effects of buoyancy force, contact angle, strata dip, etc	Miscible vertical core displacement
49	Dumore^22^	Dumore critical injection rate ($u_{{{\text{st}}}}$)	$u_{{{\text{st}}}} = \left( {\frac{d\rho }{{d\mu }}} \right)_{\min } kg$	The model incorporates the transition zone that develops as a result of diffusion and mixing	Ignores the effects of buoyancy force, contact angle, strata dip, etc	Downward miscible displacements
50	Slobod and Howlett^23^, Dumore^22^	Slobod and Howlett critical rate ($u_{c}$)	$u_{c} = kg\frac{\Delta \rho }{{\Delta \mu }}$	The model incorporates a small hypothetical protrusion of one of the fluids into the other	Ignores the effects of the transition zone between the displacing and displaced fluids and buoyancy force, contact angle, strata dip, etc	Miscible displacement in vertical unconsolidated porous media
51	Hill^24^, Guo, et al.^25^	Hill critical injection rate ($V_{{\text{c}}}$)	$V_{{\text{c}}} = \frac{2.741\Delta \rho k\sin \theta }{{\varphi \Delta \mu }}$	The ratio of the product of the density difference, permeability and sine of the strata dip, to the product of the porosity and the viscosity difference	Ignores the effects of inertial force, capillary force, contact angle, strata dip, etc	Gravity drainage processes
52	Wang^26^	Wang critical injection rate ($v_{{{\text{tc}}}}$)	$v_{{{\text{tc}}}} = \frac{{k_{\angle } K_{{{\text{rog}}\angle }} \left( {2\rho_{{\text{o}}} - \rho_{{\text{g}}} } \right)g{\text{sin}}\alpha }}{{\left( {1 - \frac{{K_{{{\text{rog}}\angle }} \mu_{{\text{g}}} }}{{K_{{{\text{rg}}\angle }} \mu_{{\text{o}}} }}} \right)\mu_{{\text{o}}} }}$	The ratio of the sum of the difference between the injected gas buoyancy and gravity, and the crude oil gravity, to the difference of the driving pressure gradient of crude oil and injected gas	Ignores the effects of oil–water/gas–water capillary forces	Crestal gas injection for stable flooding processes

It is evident that the existing dimensionless index group and critical rate models have played a pivotal role in guiding the study of stable gas flooding development in oil and gas reservoirs and project practices. However, the existing model is not suitable for the study of artificial gas cap flooding, which is mainly developed by gas cap expansion, and there are still the following limitations: Firstly, the existing models are unable to accurately characterise the development methods of gas assisted gravity drainage and artificial gas cap flooding due to their incomplete description of the gas flooding mechanism and their failure to consider the full range of influencing factors. Secondly, the existing stable gas flooding models, conditions and evaluation methods are complicated and not convenient to promote the application, such as the Dombrowski–Brownell index and the new combined dimensionless group. Thirdly, the majority of extant stable gas flooding models, conditions and influencing factors are based on research on the mechanism of gas–assisted gravity flood, which is mainly driven by gravity differentiation, while there is little research on the model, conditions and influencing factors of artificial gas cap stable flooding, which is mainly driven by gas cap expansion. Fourthly, the existing gas drive mechanism is based on the theory of elastic gas drive and does not involve the theory of rigid gas drive. The latter has strong replacement energy, high oil displacement efficiency and a sweep coefficient close to 1.0. In-depth and systematic research into the theory and technology of rigid gas drive is lacking in current academic circles. Comparative studies have found that the dimensionless group model is easier to understand the nature of gas flooding mechanism than the critical rate model, easier to analyse and compare, simpler to apply, and more applicable to a wider range of scenarios, e.g., indoor experiments, field tests, etc.

However, the extant theory and technology of crestal gas injection elastic gas drive are inadequate in meeting the strategic demand for CO_2_ large-scale enhancement of oil recovery and storage (CCUS-EOR) on a global scale. This is due to the fact that it seriously restricts the solutions to the problems of efficient recovery of crude oil and permanent reduction in CO_2_ emissions in CO_2_-EOR projects. Consequently, in order to achieve ‘significantly CO_2_-EOR and large-scale CO_2_ storage’, there is an imperative for research and development of CO_2_ gas flooding and geological storage technology. The primary emphasis of this technology is the injection of CO_2_ into the crest of an oil reservoirs, with the objective of creating an artificial gas cap. This process involves the immiscible rigid and stable gas flooding of the reservoir, with the aim of enhancing oil recovery and facilitating geological storage. This technology is of the utmost importance to the theory and technology and projects of CCUS-EOR. In view of the above limitations, this paper takes CO_2_ injection as an example. The present study is founded upon a comprehensive understanding of the mechanisms of crestal gas injection flooding、gas assisted gravity drainage and artificial gas cap stable oil flooding, as well as the geometric relationship of Dietz mode^20^. It is also based on the theory of multiphase flow. The paper proposes a brand-new development technology of artificial CO_2_ cap immiscible rigid and stable gas flooding. A novel ‘dual-carbon’ bottoming development technology involving the use of artificial CO_2_ cap immiscible rigid and stable flooding for CO_2_ EOR and sequestration has been proposed. This technology has the potential to enhance significantly oil recovery and facilitate large-scale CO_2_ sequestration. In addition, the mechanism of the artificial CO_2_ gas cap immiscible rigid and stable gas flooding has been elucidated from the seepage mechanics perspective and a gas–oil interface stability model of the artificial CO_2_ cap immiscible gas flooding would be established, which would facilitate accurately characterise the gas flooding mechanism. The stabilisation conditions of the gas–oil interface and its primary influencing factors have also been clarified. The outcomes of this study elucidated the essence of various gas flooding oil mechanisms, including but not limited to crestal gas injection flooding, gas–assisted gravity drainage and gas cap elastic or rigid gas flooding under CO_2_ injection. Moreover, the findings of this study will furnish a theoretical foundation and technical guidance for the research and development of key technologies for artificial CO_2_ gas cap gas flooding. This has been found to greatly improve crude oil recovery and large-scale geological storage of CO_2_, as well as the efficient development of attic oil and bypass oil in tectonic and carbonate oil reservoirs or abandoned oil reservoirs, including but not limited to fault block reservoirs, especially oil reservoirs with dipping strata in the later stage of extremely high water cut development, etc. It is evident that they can also provide new ideas for the research and development, promotion and application of new technology of CCUS-EOR. Furthermore, they can provide theoretical and technological support for the reduction of CO_2_ emissions (carbon neutrality) and thus have great theoretical and practical significance.

## Mechanism of artificial CO_2_ gas cap immiscible rigid stable gas flooding

**Fig. 1:**
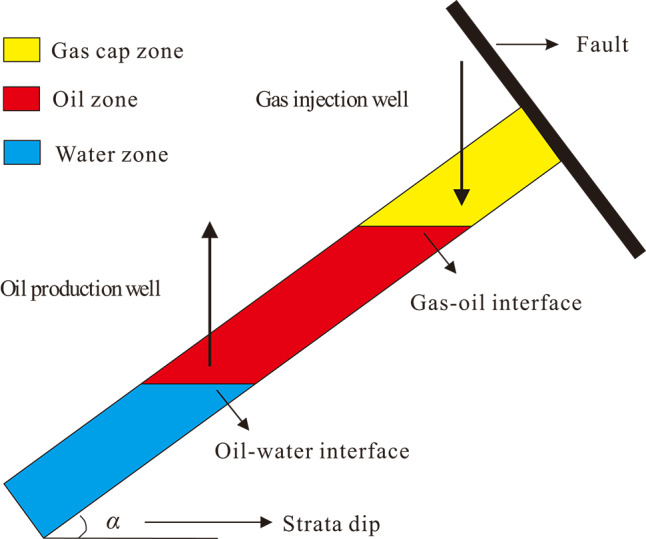
Schema illustrates the fundamental mechanism of artificial gas cap flooding in the fault block reservoir with a specific stratum dip.

Basically, artificial gas cap flooding is a secondary development method that forms a secondary artificial gas cap of a certain energy and size after injecting gas (such as N_2_ or CO_2_) at the top of inclined reservoirs. In terms of the process, gas–assisted gravity drainage for staggered injection and extraction offer a rough approximation of this development technique. With this gas cap flooding method, the producing wells rely mainly on the expansion energy of the artificial gas cap for hydrocarbon recovery (as shown in Fig. 1).

However, studies on the injection of CO_2_ to form an artificial CO_2_ gas cap have shown that, in this case, the gas injection does not significantly increase the formation energy, but relies mainly on the expansion of the CO_2_ gas to drive off the crude oil. Consequently, from the perspective of the flooding method, the artificial N_2_ gas cap flooding method is classified as elastic gas pressure driving, which is suitable for single-well throughput extraction in small-scale, complex, small-fracture reservoirs. The defining characteristic of the elastic gas drive is that when the volume of the gas cap is minimal and there is no gas injection, with the continual increase of the cumulative oil production, the crestal gas cap continues to expand while the bottom water body persists in intruding. This results in the expanding volume of the gas cap and the water body being equal to the volume of the cumulative oil production. Accordingly, the change in oil reservoir pressure under gas–driven conditions is dependent on the ratio of the volume of the gas cap to the oil zone. As formation pressure declines, a portion of the dissolved gas is separated from the oil, either replenishing the gas cap or forming solution gas drive. Additionally, the elastic energy of the rock, bound water, and the water body, including the region of water intrusion, contributes to the oil displacement. However, these displacement mechanisms have a limited impact on the effectiveness of elastic gas drive. Therefore, the formation energy will persist in being consumed, even in the event of a reduction in the transient liquid production rate or cessation of production. In such instances, the formation pressure will not revert to its original state. As the formation pressure declines, the transient oil production rate also declines, while the gas saturation, relative permeability, and gas–oil ratio increase. The aforementioned analysis demonstrates that while a reduction in formation pressure may facilitate an increase in oil displacement, the elastic energy of the rock, along with bound water and water bodies (including water invasion regions), contribute to oil displacement. Consequently, a portion of the oil reservoir pore volume is inevitably lost, rendering it challenging to restore the formation pressure to its initial level prior to the commencement of the current injection-production cycle at each gas injection cycle. Consequently, as the number of injection-production cycles increases, the formation pressure may continue to decrease, the oil reservoir pore space will also gradually decrease, and as the formation pressure decreases, so does the geological storage of CO_2_ (relative to the original formation pressure conditions). Thus, it is impossible to achieve large-scale geological storage of CO_2_ using artificial gas cap flooding, and it cannot greatly improve crude oil recovery (i.e., lower crude oil recovery in the development processes of rock, bound water, water elastic drive and dissolved gas drive). For this reason, the development of new, immiscible, stable gas drive technology has become inevitable.

**Fig. 2:**
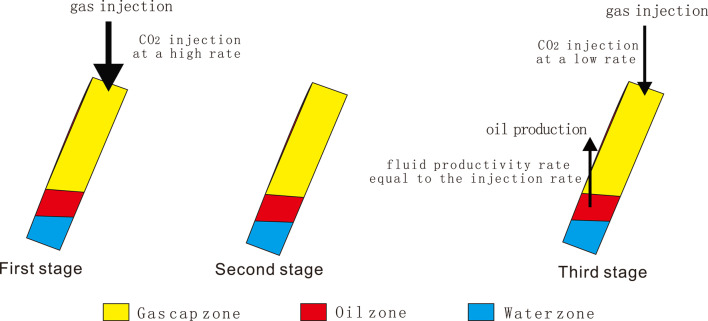
Schema illustrates injection-production process of artificial gas cap flooding under CO_2_ injection in the fault block reservoir with a specific stratum dip.

It has been demonstrated through research that the formation of a rigid gas drive is contingent upon three key factors: the volume of the artificial CO_2_ gas cap of the oil reservoirs with stratigraphic dip is large, the gas cap or oil formation is artificially injected with gas and the injection volume is large enough to maintain the formation pressure unchanged throughout the development process, and the oil reservoir does not release elastic energy and the volume of the reservoir space remains constant. In practice, given that maintaining a constant formation pressure solely through the gas cap is uncommon, it is imperative that CO_2_ be continuously injected into the gas cap throughout the entire production cycle to genuinely attain the rigid gas drive. In comparison to the elastic gas drive, the formation pressure, production rate and production gas–oil ratio in the rigid gas drive remain largely unaltered. At this juncture, the driving force of the oil reservoir is solely the expansion energy of the gas cap. The implementation of stable gas flooding allows the oil reservoir to achieve not only large-scale CO_2_ geological storage, but also a high oil recovery percentage. In view of the aforementioned points, the CO_2_ gas cap immiscible rigid stable gas flooding process can be delineated as a driving mode that not only promotes geological sequestration of CO_2_, but also significantly improves crude oil recovery. The process of artificially inducing a gas cap immiscible rigid gas pressure flooding involves the creation of an artificial gas cap that can provide sufficient energy and possesses a substantial volume. This is achieved by injecting a substantial quantity of CO_2_ into the upper region of oil reservoirs with stratigraphic dip. The production wells depend on the expansion energy of the gas cap for recovery, while the gas injection wells continue to inject CO_2_ into the gas cap (as illustrated in Fig. 2). In this flooding method, the instantaneous or cumulative injection/extraction ratio is maintained at value of 1, thereby ensuring the stability of the extraction process, as sufficient gas compensates for the extraction volume the process of generating an artificial CO_2_ gas cap, the gas–oil and oil-water interfaces decrease in level as the gas cap undergoes continuous expansion. This results in the attic oil, which is situated in the elevated region of the structure, and the flow-around oil, which is located in the central portion, being flooded to the lower section of the structure. The elevation of the oil-water interface can reach a level at the original oil-water interface or below the original oil-water interface of the reservoir, facilitating the enrichment of attic oil and bypass oil in oil reservoirs with stratigraphic dip. Concurrently, under the temperature and pressure conditions of the reservoir, part of the CO_2_ will dissolve and diffuse in the crude oil, thereby reducing its viscosity and further promoting the flow of crude oil. Following the injection of CO_2_, the tension at the gas–oil interface is found to be considerably lower than that at the oil-water interface. This disparity in gas and liquid densities is also conducive to reducing the effect of capillary pressure, thereby enhancing the crude oil recovery. In the actual production process, with the continuous expansion of the artificial CO_2_ gas cap, the decline of the gas–oil interface and the stabilization of the oil-water interface make the gas injection completely compensate for the liquid extraction, maintain the injection and extraction ratio of 1, and reach the goal of extracting the attic oil and bypass oil from the complex fault block reservoir.

The overall extraction process can be divided into three stages (Fig. 2): the first stage is large-scale gas injection to increase energy. In this stage, the rapid injection of a large amount of CO_2_ and the subsequent shutdown of the oil extraction wells results in the gathered gas forming a substantial artificial CO_2_ gas cap at the top of the reservoir. This persists until the reservoir’s stratum pressure is restored to its original state. In the second stage, the gas well is smothered for the purpose of enrichment and oil reservoir formation. In this phase, the gas and oil production wells are closed to enable the accumulation of the injected CO_2_, leading to the formation of a substantial artificial CO_2_ gas cap with considerable energy and volume. Concurrently, this phase enables the residual oil to be more thoroughly and completely re-enriched into oil reservoirs. The third stage involves stable injection and recovery. In this phase, the focus is on continuous small-displacement CO_2_ injection, accompanied by the opening of production wells. This is done to maintain the fluid production level at the formation conditions to be equal to the amount of CO_2_ injected. This process continues until the crude oil in the reservoir is fully recovered. The employment of effective gas flooding plays a pivotal role in optimising CO_2_ utilisation, sequestration and achieving enhanced crude oil recovery.

## Modelling of artificial CO_2_ gas cap immiscible rigid stable gas flooding

### Critical rate modelling

The following assumptions underpin the flow model of the fault block reservoir with a specific stratum dip: (1) The oil reservoir exhibits water-wet wettability. (2) The reservoir is a homogeneous porous medium, saturated with oil, gas and bound water. (3) The formation rock, oil and water are incompressible. (4) The reservoir temperature remains constant. (5) The artificial gas cap procedure under CO_2_ injection is an immiscible flooding process. (6) The gas–oil and oil–water interfaces exhibit uniform migration throughout the artificial gas cap process under CO_2_ injection. (7) The flow of oil, gas and water is in accordance with Darcy’s law. (8) There is no physical or chemical reaction between the injected CO_2_ and the formation fluid. (9) The dissolution of CO_2_ in crude oil is rapid. (10) Given that CO_2_ is only a slightly soluble solvent in water within the temperature and pressure range suitable for common EOR methods, it can only slightly increase the viscosity of formation water and reduce its density. However, its density change is smaller than that predicted by the ideal dissolution theory^27^. In light of the aforementioned evidence, the dissolution of CO_2_ in formation water is disregarded in this paper.

**Fig. 3:**
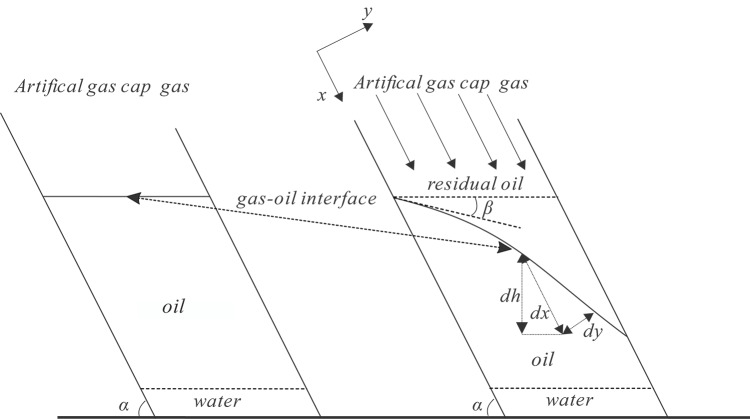
The schema of gas oil interface of artificial gas cap flooding in the fault block reservoir with a specific stratum dip and Dietz geometrical model (Modified after Dietz^20^, Ren, et al.^9^).

In accordance with the aforementioned assumptions, the injection of gas into the upper portion of the oil layer with a stratigraphic dip of $\alpha$ will result in the formation of an immiscible gas oil interface, as depicted in Fig. 3. If the artificial gas cap flooding under CO_2_ injection is an instability displacement processes, the gas and oil flow rate at the gas oil interface will be unequal, resulting in an unstable interface and the occurrence of channeling. This, in turn, leads to a significant reduction in sweep efficiency, which negatively impacts the development effect of the flooding. Otherwise, if the artificial gas cap flooding under CO_2_ injection is a stable displacement, the gas and oil flow rate at the gas oil interface will be equal, the interface will be stable and uniform, and the gas will theoretically be able to spread to the entire oil region. This results in a sweep efficiency of close to 1.0, thereby indicating a favourable development effect for the artificial gas cap flooding process. It is evident that the rigid stable gas cap flooding under CO_2_ injection exhibits the optimal development effect. As illustrated in Fig. 3, it is postulated that there is an interface angle of $\beta$ between the gas oil interface and the horizontal plane during the gas cap flooding under CO_2_ injection in the fault block reservoir with a specific stratum dip of $\alpha$. In the context of the fault block reservoir, if the water saturation present within the oil and gas zones is defined as the irreducible water saturation, it can be deduced that the flow phases situated above the oil water interface of the reservoirs consist exclusively of crude oil and gas from the cap. Consequently, the volume of crude oil displaced by CO_2_ from the gas cap in pores per unit volume above the gas oil interface can be expressed as follows:

1 $$S_{{{\text{gp}}}} = 1 - S_{{{\text{wi}}}} - S_{{{\text{or}}}}$$

$\alpha$ is a stratum dip of the fault block reservoir, (°); $\beta$ is an interface angle between the gas oil interface and the horizontal plane in the process of artificial gas cap flooding under CO_2_ injection, (°); $dh$, $dx$, $dy$ are the differential forms of migration distance of gas from cap and crude oil in the vertical direction, strata direction (i.e. x-axis direction), and perpendicular to strata direction (i.e. y-axis direction) respectively, m.

The buoyancy of gas in an artificial CO_2_ gas cap in pores per unit volume can be expressed as follows:

2 $$f_{{\text{g}}} = \rho_{{\text{o}}} S_{{{\text{wp}}}} g$$

Upon substituting Eq. (1) into Eq. (2), the following equation is obtained:

3 $$f_{{\text{g}}} = \rho_{{\text{o}}} \left( {1 - S_{{{\text{wi}}}} - S_{{{\text{or}}}} } \right)g$$

At this point, the gravitational force on the artificial CO_2_ gas cap per unit volume of pore space can be expressed as follows:

4 $$G_{{\text{g}}} = \rho_{{\text{g}}} \left( {1 - S_{{{\text{wi}}}} - S_{{{\text{or}}}} } \right)g$$

Subsequently, the disparity between the buoyancy and gravitational forces exerted on the artificial CO_2_ gas cap per unit volume of pore space can be articulated as follows:

5 $$F_{{\text{g}}} = \left( {\rho_{{\text{o}}} - \rho_{{\text{g}}} } \right)\left( {1 - S_{{{\text{wi}}}} - S_{{\text{o}}} } \right)g$$

Therefore, the gradient of the difference between the buoyancy force and the gravitational force per unit volume of the artificial CO_2_ gas cap in the vertical direction is as follows:

6 $$\frac{{\partial F_{{\text{g}}} }}{\partial h} = \left( {\rho_{{\text{o}}} - \rho_{{\text{g}}} } \right)g$$

As illustrated in Fig. 3, the gradient of the difference between buoyancy and gravity per unit volume of the artificial CO_2_ gas cap in the direction of the gas strata can be expressed by the following equation:

7 $$\frac{{\partial F_{{\text{g}}} }}{\partial x} = \left( {\rho_{{\text{o}}} - \rho_{{\text{g}}} } \right)g\sin \alpha$$

Similarly, the gravity gradient per unit pore volume of crude oil can be expressed in terms of both the vertical and the stratigraphic directions at the gas–oil interface.

8 $$\frac{{\partial G_{{\text{o}}} }}{\partial h} = \rho_{{\text{o}}} g$$

9 $$\frac{{\partial G_{{\text{o}}} }}{\partial x} = \rho_{{\text{o}}} g\sin \alpha$$

In accordance with the multiphase flow theory^28,29^, the flow velocities of two-phase flows (i.e. oil–water, gas–water, and oil–gas) can be calculated. The velocities of the artificial CO_2_ gas cap gas and crude oil flowing in the direction of the stratum per unit of pore volume on the gas–oil interface during the stable flooding of the artificial CO_2_ gas cap in the fault block reservoirs can be expressed as follows, respectively.

10 $$v_{{{\text{g}}\angle }} = - \frac{{k_{\angle } K_{{{\text{rg}}\angle }} }}{{\mu_{{\text{g}}} }}\left[ {\frac{{\partial p_{{\text{g}}} }}{\partial x} + \frac{{\partial F_{{\text{g}}} }}{\partial x} - \frac{{\partial p_{{{\text{c}}\left( {\text{g - w}} \right)S_{{{\text{wi}}}} }} }}{\partial x}} \right]$$

11 $$v_{{{\text{o}}\angle }} = - \frac{{k_{\angle } K_{{{\text{rog}}\angle }} }}{{\mu_{{\text{o}}} }}\left[ {\frac{{\partial p_{{\text{o}}} }}{\partial x} - \frac{{\partial G_{{\text{o}}} }}{\partial x} - \frac{{\partial p_{{{\text{c}}\left( {\text{o - w}} \right)S_{{{\text{wi}}}} }} }}{\partial x}} \right]$$

In accordance with the definition of capillary, the capillary of gas–water and oil–water under conditions of irreducible water saturation are equivalent to their maximum values. Consequently, the capillary gradients can be calculated as follows:

12 $$\frac{{\partial p_{{{\text{c}}\left( {\text{g - w}} \right)S_{{{\text{wi}}}} }} }}{\partial x} = 0$$

13 $$\frac{{\partial p_{{{\text{c}}\left( {\text{o - w}} \right)S_{{{\text{wi}}}} }} }}{\partial x} = 0$$

Then, Eqs. (10) and (11) can be transformed into the following Eqs.:

14 $$v_{{{\text{g}}\angle }} = - \frac{{k_{\angle } K_{{{\text{rg}}\angle }} }}{{\mu_{{\text{g}}} }}\left[ {\frac{{\partial p_{{\text{g}}} }}{\partial x} + \frac{{\partial F_{{\text{g}}} }}{\partial x}} \right]$$

15 $$v_{{{\text{o}}\angle }} = - \frac{{k_{\angle } K_{{{\text{rog}}\angle }} }}{{\mu_{{\text{o}}} }}\left[ {\frac{{\partial p_{{\text{o}}} }}{\partial x} - \frac{{\partial G_{{\text{o}}} }}{\partial x}} \right]$$

By substituting Eqs. (7) and (9) into Eqs. (14) and (15), the following result is obtained:

16 $$v_{{{\text{g}}\angle }} = - \frac{{k_{\angle } K_{{{\text{rg}}\angle }} }}{{\mu_{{\text{g}}} }}\left[ {\frac{{\partial p_{{\text{g}}} }}{\partial x} + \left( {\rho_{{\text{o}}} - \rho_{{\text{g}}} } \right)g\sin \alpha } \right]$$

17 $$v_{{{\text{o}}\angle }} = - \frac{{k_{\angle } K_{{{\text{ro}}\angle }} }}{{\mu_{{\text{o}}} }}\left[ {\frac{{\partial p_{{\text{o}}} }}{\partial x} - \rho_{{\text{o}}} g\sin \alpha } \right]$$

In the context of the immiscible rigid stable gas flooding, the following characteristics are exhibited by artificial gas cap flooding:

18 $$v_{{{\text{g}}\angle }} = v_{{{\text{o}}\angle }} = v_{{\text{t}}}$$

By combining Eq. (16) to Eq. (18), we can derive the following Eqs.:

19 $$\left( {\frac{{\mu_{{\text{o}}} }}{{k_{\angle } K_{{{\text{rog}}\angle }} }} - \frac{{\mu_{{\text{g}}} }}{{k_{\angle } K_{{{\text{rg}}\angle }} }}} \right)v_{{\text{t}}} = \frac{{\partial \left( {p_{{\text{g}}} - p_{{\text{o}}} } \right)}}{\partial x} + \left( {2\rho_{{\text{o}}} - \rho_{{\text{g}}} } \right)g\sin \alpha$$

From the definition of the capillary, we can express the following equation:

20 $$\frac{{\partial p_{{{\text{c}}\left( {\text{g - o}} \right)}} }}{\partial x} = \frac{{\partial \left( {p_{{\text{o}}} - p_{{\text{g}}} } \right)}}{\partial x}$$

In the context of the vertical mechanical equilibrium, the capillary between the gas of the artificial gas cap and the formation crude oil, can be expressed as follows:

21 $$p_{{{\text{c}}\left( {\text{g - o}} \right)}} = \left( {\rho_{{\text{o}}} - \rho_{{\text{g}}} } \right)gh$$

22 $$\frac{{\partial p_{{{\text{c}}\left( {\text{g - o}} \right)}} }}{\partial x} = \left( {\rho_{{\text{o}}} - \rho_{{\text{g}}} } \right)g\frac{\partial h}{{\partial x}}$$

In accordance with the geometric relationship illustrated in Fig. 3, the following can be derived:

23 $$dh = - dy\cos \alpha$$

24 $$\tan \left( {\alpha - \beta } \right) = \frac{dy}{{dx}}$$

It can thus be demonstrated that the capillary in the dipping formation of the fault block reservoir can be expressed as follows:

25 $$\frac{{\partial p_{{{\text{c}}\left( {\text{g - o}} \right)}} }}{\partial x} = - \left( {\rho_{{\text{o}}} - \rho_{{\text{g}}} } \right)g\cos \theta \tan \left( {\alpha - \beta } \right)$$

By substituting Eq. (25) into Eq. (19), the interface angle ($\beta$) may be expressed as follows:

26 $$\tan \left( {\alpha - \beta } \right) = \frac{{\left( {1 - \frac{{K_{{{\text{rog}}\angle }} \mu_{{\text{g}}} }}{{K_{{{\text{rg}}\angle }} \mu_{{\text{o}}} }}} \right)\mu_{{\text{o}}} v_{{\text{t}}} - k_{\angle } K_{{{\text{rog}}\angle }} \left( {2\rho_{{\text{o}}} - \rho_{{\text{g}}} } \right)g\sin \alpha }}{{k_{\angle } K_{{{\text{rog}}\angle }} \left( {\rho_{{\text{o}}} - \rho_{{\text{g}}} } \right)g\cos \alpha }}$$

From Eq. (26), we can gain insight into the factors affecting the stability of the gas oil interface in the artificial CO_2_ gas cap flooding process, specifically the formation gas density and formation crude oil density under reservoir conditions. in addition, the following factors must be considered: air permeability in the stratigraphic direction, relative permeability of the gas phase from artificial CO_2_ gas cap, relative permeability of the liquid phase, flow rate of the gas phase from the artificial CO_2_ gas cap or the formation crude oil, viscosity of the gas phase from the artificial CO_2_ gas cap, viscosity of the formation crude oil, strata dip, and so on. As illustrated in Fig. 3, when $\beta = \alpha$, the gas oil interface is parallel to the strata, the interface is extremely unstable, and therefore there is an inevitable of gas channeling or fingering phenomenon; when $\beta < \alpha$, the gas oil interface is stable, and therefore there is only a likelihood of gas tonguing phenomenon and an impossibility of gas breakthrough phenomenon. It is evident that a decrease in the gas oil interface angle ($\beta$) will enhanced the stability of the gas oil interface during the artificial CO_2_ gas cap flooding process, resulting in a sweep efficiency approaching 1.0. This will lead to optimal development of the artificial CO_2_ gas cap flooding processes. Furthermore, as demonstrated in Eq. (26), as the interface angle ($\beta$) approaches the strata dip ($\alpha$), the gas invasion rate ($v_{{\text{t}}}$) in the strata direction during the artificial CO_2_ gas cap flooding process reaches a maximum value ($v_{{{\text{tc}}}}$), which can be defined as the critical rate at which gas displacing oil front becomes unstable (i.e. fingering occurs). Therefore, the $v_{{{\text{tc}}}}$ can be expressed as follows:

27 $$v_{{{\text{tc}}}} = \frac{{k_{\angle } K_{{{\text{rog}}\angle }} \left( {2\rho_{{\text{o}}} - \rho_{{\text{g}}} } \right)g{\text{sin}}\alpha }}{{\left( {1 - \frac{{K_{{{\text{rog}}\angle }} \mu_{{\text{g}}} }}{{K_{{{\text{rg}}\angle }} \mu_{{\text{o}}} }}} \right)\mu_{{\text{o}}} }}$$

As demonstrated in Eqs. (26–27), the reduction of the interface angle ($\beta$) enables the implementation of artificial gas cap stable flooding within the fault block reservoir, contingent upon the satisfaction of specific stratum dip conditions. That is to say, there exist conditions that must be met in order to ensure the stability of the gas displacing oil front for artificial gas cap flooding, as outlined below:

28 $$v_{{\text{t}}} { = }v_{{{\text{tc}}}}$$

From Eqs. (27) and (28), it is possible to deduce the factors that influence the critical rate or conditions required to maintain the stability of the gas oil interface during the artificial gas cap flooding process, particularly in reservoir conditions. These factors include the formation gas density, formation crude oil density, air permeability in the strata direction, relative permeability of the gas phase in artificial gas cap, relative permeability of the liquid phase, formation gas viscosity, formation crude oil viscosity, strata dip, and other relevant parameters. It can be observed that the factors which affect the conditions required to maintain the stability of gas oil interface are the formation gas density, formation crude oil density, air permeability in the strata direction, relative permeability of the gas phase of artificial gas cap, relative permeability of the liquid phase, formation gas viscosity, formation crude oil viscosity, and the strata dip. Additionally, it has been observed that the critical rate values associated with the artificial gas cap stable flooding processes demonstrate variability across various experimental scales, encompassing laboratory experiments, pilot field trials, and field tests. Consequently, the critical rate model experiences constraints in its applicability when employed at different scales or in varied scenarios.

### Dimensionless number group modelling

In comparison to the critical rate models, the dimensionless group models offer a more straightforward understanding of the underlying gas drive mechanism. Furthermore, they provide a convenient framework for analyzing and comparing data from laboratory experiments, pilot field trials, field tests, and numerical reservoir simulations, as well as other scenarios. Their simplicity and versatility make them a more suitable choice for scientific discourse. Consequently, Eq. (27) can be transformed into the following equation:

29 $$\frac{{k_{\angle } K_{{{\text{rog}}\angle }} \left( {2\rho_{{\text{o}}} - \rho_{{\text{g}}} } \right)g{\text{sin}}\alpha }}{{\left( {1 - \frac{{K_{{{\text{rog}}\angle }} \mu_{{\text{g}}} }}{{K_{{{\text{rg}}\angle }} \mu_{{\text{o}}} }}} \right)\mu_{{\text{o}}} v_{{{\text{tc}}}} }} = 1$$

And if set:

30 $$N_{{{\text{GOI}}}} = \frac{{k_{\angle } K_{{{\text{rog}}\angle }} \left( {2\rho_{{\text{o}}} - \rho_{{\text{g}}} } \right)g{\text{sin}}\alpha }}{{\left( {1 - \frac{{K_{{{\text{rog}}\angle }} \mu_{{\text{g}}} }}{{K_{{{\text{rg}}\angle }} \mu_{{\text{o}}} }}} \right)\mu_{{\text{o}}} v_{{\text{t}}} }}$$

As demonstrated in Eq. 30, the newly proposed comprehensive dimensionless group ($N_{{{\text{GOI}}}}$) is instrumental in quantifying the stability of the gas oil interface during the process of artificial gas cap gas flooding under CO_2_ injection. This group can be regarded as a multifaceted metric, serving not only as an assessment model for artificial CO_2_ gas cap stable gas flooding but also as a means to evaluate the gas oil interface during the process of artificial gas cap immiscible stable gas flooding or artificial gas cap immiscible rigid stable gas flooding under CO_2_ injection. Furthermore, Eq. (30) can be transformed as follows:

31 $$N_{{{\text{GOI}}}} = \frac{{\left( {k_{\angle } \rho_{{\text{o}}} g + \varphi \mu_{{\text{o}}} v_{{\text{t}}} N_{{\text{G}}} } \right)K_{{{\text{rog}}\angle }} {\text{sin}}\alpha }}{{\left( {1 - \frac{{K_{{{\text{rog}}\angle }} \mu_{{\text{g}}} }}{{K_{{{\text{rg}}\angle }} \mu_{{\text{o}}} }}} \right)\mu_{{\text{o}}} v_{{\text{t}}} }}$$

In order to facilitate comparison with previous studies, it is proposed that, based on the nomenclature principle of the dimensionless groups used by predecessors to describe gas cap flooding, thus, $N_{{{\text{GOI}}}}$ should be temporarily designated a Comprehensive Gravity number of the artificial gas cap immiscible stable gas flooding under CO_2_ injection, or the artificial gas cap immiscible stable gas flooding number.

In conjunction with the aforementioned Eqs. (28), (29), (30) and (31), the conditions necessary to maintain the stability of artificial gas cap stable flooding can be determined as follows:

32 $$N_{{{\text{GOI}}}} > 1$$

As demonstrated in Eq. (32), as the interface angle ($\beta$) is reduced, there is a possibility that the gas oil interface will become stable, thereby allowing the development mode of artificial gas cap stable flooding to be realized. It is evident from the associative Eqs. (30), (31) and (31) that, provided Eq. (31) is satisfied, $N_{{{\text{GOI}}}}$ is independent of the type of injected gas. This finding indicates that the assessment model of the dimensionless group ($N_{{{\text{GOI}}}}$) of artificial gas cap immiscible rigid stable gas under CO_2_ injection is applicable to reservoirs where the gas cap is formed by CO_2_, N_2_, natural gas, air, or flue gas, and so on. It was also found that this equation is applicable to artificial CO_2_ gas cap immiscible stable gas flooding, and its validity is not contingent on the nature of the gas flooding process, whether rigid or elastic. The applicability of the equation is determined by the specific characteristics of the gas flooding process, as defined. To elaborate, the categorisation of gas flooding as either rigid or elastic is contingent upon the precise definition of these gas displacement processes.

## Model validation

### Field test validation

#### Parameters

A review of the extant literature reveals a paucity of laboratory experimental studies on gas cap flooding that test or consider the permeability of each phase. Consequently, the new model ($N_{{{\text{GOI}}}}$) proposed in this paper would verify by field tests. The current state of research on the lean gas injection project in the Handil Main Zone^2,30^, the air injection project in the West Hackberry Oilfield^31–33^, the gas/inert gas (i.e. CO_2_ and N_2_) injection project of Hawkins (Woodbine) Dexter Sand^34,35^, and the N_2_ injection pilot of Yanling Oilfield^36–41^ have been proved that the process of artificial gas cap flooding had been realised, and the remarkable development results had been achieved. Of these, the geological and development data of West Hackberry Field, where an artificial flue gas cap (AFGC) was formed by air injection (following the injection of air into the reservoir, the high-temperature oxidation (HTO) and low-temperature oxidation (LTO) reactions will ensue, resulting in the generation of flue gas) for the purpose of stable flooding development, and the pilot region of Yanling Oilfield, where an artificial gas cap was formed by N_2_ injection for the purpose of stable flooding development, are the most complete. This is particularly true of the gas–liquid relative permeability curves, which can meet the needs of the modified model ($N_{{{\text{GOI}}}}$) that was built in this paper for the purpose of carrying out verification and comparative study. The reservoir characteristics of the West Hackberry Oilfield and the pilot region of the Yanling Oilfield are presented in Table 2, the fluid parameters of the injected gas in the West Hackberry Oilfield and the pilot region of the Yanling Oilfield are provided in Table 3, and the oil and injected gas components and their molar content of the West Hackberry Oilfield and the pilot region of the Yanling Oilfield are presented in Table 4.

**Table 2:** Reservoir characteristics in the West Hackberry field and the Yanling Oilfield^31–33,39–41^.

Parameter type	West Hackberry		Yanling	
	Range of value	Av. value	Range of value	Av. value
*Reservoir characteristics*				
Rock type	Sand stone	–	Carbonatite	–
Strata dip (°)	23–35	29	20–25	22.5
Porosity (%)	23.9–27.6	25.8	3.95–5.74	4.01
Permeability (mD)	300–1000	650	452–4735	2000
Mean pore throat radius (μm)	3.54–6.94	5.39	7.94–30.97	24.46
Kv/Kh ratio	1.0	–	0.6–33	9.3
Pay(m)	9.14–9.45	9.30	48.39–237	86
S_wc_	19–23	21	12.52–33.22	27.89
Reservoir temperature (℃)	90.56–96.11	93.34	118	–
Initial formation pressure (MPa)	24.13–29.64	26.89	30.12	–
*Processes data*				
Project scope	Fieldwide	–	North hill head	–
Start date	11/1994	–	10/1994	–
Project area (km^2^)	1.54	–	2.904	–
Injected gas	Air	–	N_2_	–
Injection mode	Secondary	–	Secondary	–
Injection strategy	Immiscible	–	Immiscible	–
Displacement rate (m/d)	0.029–0.060	0.0445	0.0177–0.1162	0.0948
Descent distance of gas–liquid interface (m)	8.82–18.24	13.53	4.1–30.3	17.7
*Phase behavior data*				
Oil density (g/cm^3^)	0.8529–0.8633	0.8602	0.8148–0.8590	0.8369
Oil viscosity (mPa s)	0.8382–1.0964	0.9	8.21–15.9	11.3
Oil compressibility(10^−4^ MPa^−1^)	11	–	9.1	–
Oil FVF at Pb	1.285	–	1.051	–
GOR (m^3^/m^3^)	500	–	2.6–3.13	2.85
MMP (MPa)	Not Avbl	–	Not Avbl	–
Saturation pressure (MPa)	28.35	–	1.35	
Water density (g/cm^3^)	1.002–1.006	1.004	1.005	–
Water viscosity (mPa s)	0.34–0.36	0.35	0.24	–
Water compressibility (10^−4^ MPa^−1^)	2.89–2.93	2.91	4	–
Water salinity (ppm)	50,000.0	–	2,943,000	–

**Table 3:** Fluid properties of the injected gas in the West Hackberry field and the Yanling Oilfield^31,32,38–40^.

West Hackberry			Yanling		
Property	Value range	Av. value	Property	Value range	Av. value
Injection gas type	Air	–	Injection gas type	N_2_	–
Flue gas–liquid interfacial tension (mN/m)	–	4.4869	N_2_-liquid interfacial tension (mN/m)	–	8.85
Contact angle (°)	–	55.25	–		
Flue gas density at Pb (g/cm^3^)	0.6432–0.6523	0.6482	N_2_ density at Pb (g/cm^3^)	0.0191–0.2485	0.1338
Flue gas density (g/cm^3^)	0.6304–0.6432	0.6368	N_2_ density (g/cm^3^)	0.2163–0.2214	0.2189
Flue gas viscosity at Pb (mPa s)	0.0195–0.0211	0.0203	N_2_ viscosity at Pb (mPa s)	0.02112–0.02803	0.02458
Flue gas viscosity (mPa s)	0.0165–0.0195	0.0185	N_2_ viscosity (mPa s)	0.02715–0.02733	0.02724
Saturation pressure (MPa)	20.13–23.05	21.59	Saturation pressure (MPa)	1.37–34.08	17.72
Flue gas FVF at Pb	0.918–1.0420	0.980	N_2_ FVF at Pb	1.041–1.083	1.061
Flue gas FVF	0.9198–1.5118	1.1859	N_2_ FVF	1.075–1.077	1.076
Flue gas compressibility factor at Pb (MPa^−1^)	0.9145–0.9241	0.9193	N_2_ compressibility factor at Pb	–	–
Flue gas compressibility factor (MPa^−1^)	0.9059–0.9241	0.9110	N_2_ compressibility factor	–	–

**Table 4:** Oil and gas phase components and their molar content^31–33,36–41^.

Components (%)	West Hackberry		Yanling	
	Oil	Flue gas	Oil	N_2_
O_2_	–	–	–	0.002
Ar	–	–	–	0.487
S	–	–	0.50	–
N_2_	1.03	85.00	–	99.5
CO_2_	0.48	15.00	–	0.01
C_1_	42.75	–	5.92	0.001
C_2_ ~ C_4_	5.39	–		
C_5_ ~ C_6_	1.95	–		
C_7_ ~ C_11_	15.92	–	59.63	
C_12_ ~ C_17_	14.89	–	18.52	
C_18+_	17.58	–	15.41	
–	At the temperature of 204.44 °C and the pressure of 24.13 MPa		At the temperature of 118 °C and the pressure of 30.1 MPa	

**Fig. 4:**
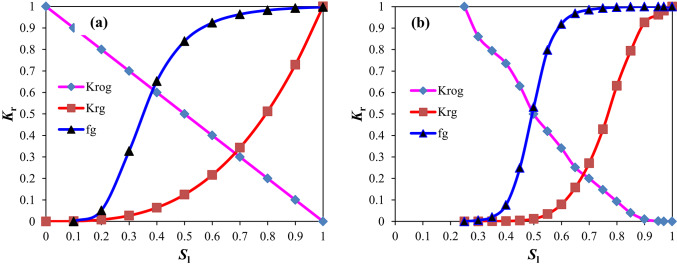
The gas–liquid relative permeability (Kr) curves from combustion tube tests (**a**) and used in flue gas displacement simulations (**b**) for the West Hackberry field^31,32^.

**Fig. 5:**
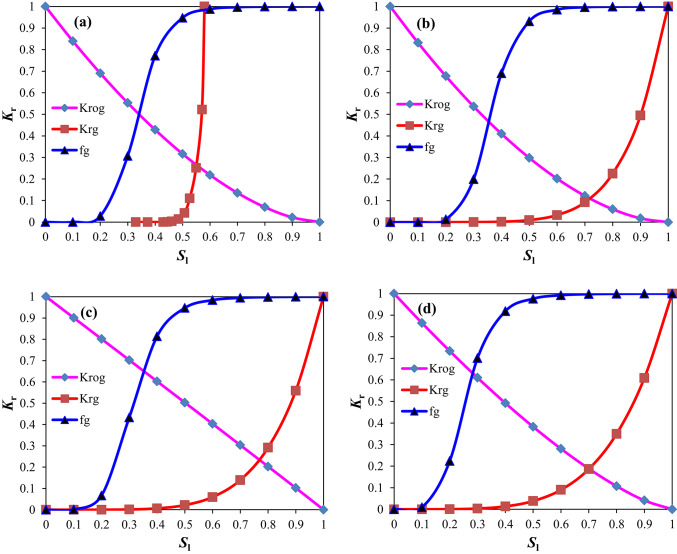
The gas–liquid relative permeability (Kr) curves (**a**), (**b**), and (**c**) for bound water–gas mixture phases and oil–gas mixture phases based on drilling matrix core tests in pilot region of the Yanling Oilfield, and the gas–liquid relative permeability (Kr) curves (**d**) for bound water–gas mixture phases and oil–gas mixture phases based on drilling fracture core tests in the pilot region of the Yanling Oilfield.

The gas–liquid relative permeability curves are presented in Figs. 4 and 5. The value of liquid saturation at the gas displacing oil front (i.e., gas–oil interface) can be obtained using the graphical solution proposed by Welge^42^ to solve the Buckley-Leverett fractional flow equation^43,44^. Upon solving the curves in the West Hackberry Oilfield, the liquid saturation value of the gas flooding front was found to be 0.42–0.54, with an average of 0.48. The corresponding liquid phase relative permeability was observed to be 0.43–0.58, with an average of 0.51, while the gas phase relative permeability was determined to be 0.032–0.071, with an average of 0.052. Similarly, the liquid saturation value of the gas flooding front was determined to be 0.3–0.41, with an average of 0.38 in the pilot region of the Yanling Oilfield. The corresponding liquid phase relative permeability was found to be 0.39–0.61, with an average of 0.51. The gas phase relative permeability was 0.002–0.01, with an average of 0.0041.

Krg is the relative permeability of gas, Krog is the oil–gas relative permeability, f_g_ is the fraction of gas in flowing stream, and *S*_l_ is the bound water–gas mixture phases saturation.

Krg is the relative permeability of gas, Krog is the oil–gas mixture phases permeability relative to bound water–gas mixture phases, f_g_ is the fraction of gas in flowing stream, and* S*_l_ is the bound water–gas mixture phases saturation.

#### Test results

The test results using reservoir parameters from the West Hackberry Oilfield and the pilot region in the Yanling Oilfield are shown in Table 5. As can be seen in Table 5, the artificial gas cap immiscible stable gas flooding number ($N_{{{\text{GOI}}}}$) in the West Hackberry Oilfield and the pilot region in the Yanling Oilfield calculated in this paper are 0.9491–14.6972, respectively. The average value for the West Hackberry Oilfield is 4.6141, while for the pilot region in the Yanling Oilfield is 0.1100–10.7918, with an average of 0.9792. These results show that the immiscible stable gas flooding has not been fully realized in both oilfields. Furthermore, the majority of the artificial gas cap flooding numbers in the former are greater than 1.0, indicating that the artificial gas cap flooding has been achieved in most regions. In contrast, the majority of the artificial gas cap flooding numbers in the latter are less than 1.0, indicating that the artificial gas cap flooding has only been achieved in a limited region and that the gas breakthrough has occurred prematurely, resulting in a failure to achieve the anticipated development effect. These findings are consistent with the results of indoor analysis and oilfield pilot tests (Kulkarni 2005, Kulkarni and Rao 2006, Dong, Jiao et al. 2016, Wang, Zeng et al. 2020). For example, in the West Hackberry Oilfield, the artificial gas cap flooding enhanced oil recovery by up to 30–40% to 90%. The impact of enhanced oil recovery was pronounced. In contrast, in the pilot region of the Yanling Oilfield, artificial gas cap flooding enhanced oil recovery by only 2.0%. The efficacy of EOR differs by approximately 20 times between the two oilfields. However, in addition to the artificial gas cap flooding number proposed in this paper, only a limited number of gravity numbers could be identified and reflected unambiguously. These include the Gravity Number ($N_{{\text{G}}} = \frac{\Delta \rho gk}{{\Delta \mu v}}$ ) proposed by Kulkarni (2005, 2006).

**Table 5:** Calculation results of different model of artifical gas cap flooding for reservoirs in the West Hackberry field and the Yanling Oilfield.

Dimensionless number models	Formula	West Hackberry		Pilot region of Yanling		Comment
		Range of value	Av. value	Range of value	Av. value	
Gravity number^2,3^	$N_{{\text{G}}} = \frac{\Delta \rho gk}{{\Delta \mu v}}$	7.589–90.140	31.344	0.856–256.067	9.793	
Gravity drainage number^4,6^	$N_{{{\text{GD}}}} = N_{{\text{G}}} + \left( {\frac{{\rho_{{\text{g}}} }}{{\rho_{{\text{o}}} }}\left( {N_{{\text{C}}} + N_{{\text{B}}} } \right)} \right)$	1750.234–16,295.963	5788.751	21.636–4468.206	244.217	
Modified gravity drainage number^6^	$N^{\prime}_{{{\text{GD}}}} = N^{\prime}_{{\text{G}}} \left( {1 + \frac{{\mu_{{\text{g}}} }}{{\mu_{{\text{o}}} }}} \right) + \frac{{\rho_{{\text{g}}} }}{{\rho_{{\text{o}}} }}\left( {N_{{\text{C,1}}} + N_{{\text{B}}} } \right)$	141.088–3012.692	739.980	1.306–419.611	18.616	
Yang Capillary number^8^	$N_{{\text{C}}} = \frac{{v\mu_{{\text{o}}} H}}{{K_{{{\text{ro}}}} \sqrt {\phi K} \sigma \cos \theta \sin \alpha }}$	0.182–0.877	0.529	–	-	
Yang variation Capillary number^8^	$N_{{{\text{Cam}}}} = \frac{{v\mu_{{\text{o}}} H}}{{\left( {S_{{\text{o}}} - S_{{{\text{or}}}} } \right)K_{{{\text{ro}}}} \sqrt {\phi K} \sigma \cos \theta \sin \alpha }}$	1.009–4.872	2.940	-	-	
Macroscopic Bond number^2,13^	$N_{{\text{B}}} = \frac{\Delta \rho gL}{{P_{{\text{c}}}^{*} }}$	16.743–19.240	18.001	233.570–1263.529	435.958	
New comprehensive number of this paper	$N_{{{\text{GOI}}}} = \frac{{k_{\angle } K_{{{\text{rog}}\angle }} \left( {2\rho_{{\text{o}}} - \rho_{{\text{g}}} } \right)g{\text{sin}}\alpha }}{{\left( {1 - \frac{{K_{{{\text{rog}}\angle }} \mu_{{\text{g}}} }}{{K_{{{\text{rg}}\angle }} \mu_{{\text{o}}} }}} \right)\mu_{{\text{o}}} v_{{\text{t}}} }}$	0.9491–14.6972	4.6141	0.1100–10.7918	0.9792	
Dumore number	$N_{{u_{{{\text{st}}}} }} = \frac{{kg\left( {\frac{d\rho }{{d\mu }}} \right)_{\min } }}{{u_{{{\text{st}}}} }}$	0.8756–5.9735	2.5441	0.1243–9.1081	0.6963	Obtained by the nondimensionalized Dumore Critical Rate^22^ (i.e. the criterion for stable displacement)

In comparison with the results of the Capillary number, Macroscopic Bond numbers, and Grattoni numbers (see Table 5), the Capillary number ($N_{{\text{C,1}}}$) in the West Hackberry Oilfield is 1.234 × 10^–9^–3.018 × 10^–9^, with an average of 2.124 × 10^–9^. The pilot region of the Yanling Oilfield exhibits the Capillary number of the pilot region of the Yanling Oilfield is (0.629 × 10^–9^–4.153 × 10^–9^, with an average of 3.377 × 10^–9^. Conversely, the calculations of the Macroscopic Bond numbers are all greater than 1.The Grattoni numbers ($N$) of the two oilfields were found to be 1.915–21.368, with an average of 8.985, and 1.905–256.630, with an average of 33.773, respectively. The observed values exceed 1, suggesting that the fluid morphology and displacement mechanisms during gas flooding are predominantly governed by density difference, i.e. gravity exerts a dominant influence. Consequently, it can be deduced that both oilfields have undergone the immiscible stable gas flooding process, thereby validating the efficacy of the the immiscible stable gas flooding as a viable technique in these specific oilfields. This finding suggests that the current set of dimensionless groups is inadequate for fully and comprehensively characterising the fluid morphology and displacement mechanisms in the context of gas flooding. Consequently, the artificial gas cap immiscible stable gas flooding number ($N_{{{\text{GOI}}}}$) may prove to be a more efficacious solution.

### Validation against the criterion for stable displacement

It is noteworthy that many scholars^20,21,23–26^ have studied the critical rate (i.e. the maximum gas injection rate at the gas displacing oil front of stable displacement), and Dumore (1964) had established the well-known and widely used Dumore criterion for immiscible stable displacement^22^.

As demonstrated in Table 5, the Dumore number ($N_{{u_{{{\text{st}}}} }}$) can be obtained by nondimensionalized the Critical Rate of Dumore criterion. The Dumore number ($N_{{u_{{{\text{st}}}} }}$) of the West Hackberry field and pilot area of Yanling Oilfield are 0.8756–5.9735, with an average of 2.5441, and 0.1243–9.1081, with an average of 0.6963, respectively. The analyses concluded that only part of the West Hackberry field and pilot area of Yanling Oilfield had achieved stable gas flooding. Furthermore, the Dumore number ($N_{{u_{{{\text{st}}}} }}$) of the pilot area of Yanling Oilfield was mostly distributed in the interval of less than 1.0, indicating that the majority of the area had not achieved stable gas flooding. In contrast, The minimum value of Dumore number ($N_{{u_{{{\text{st}}}} }}$) in West Hackberry field is closer to 1.0, and the Dumore number ($N_{{u_{{{\text{st}}}} }}$) of the West Hackberry field was predominantly distributed in the interval of larger than 1.0, suggesting that the majority of the area had achieved stable gas flooding.

It is evident that the calculation results and the research understanding of the Dumore number ($N_{{u_{{{\text{st}}}} }}$) , as well as the new model, are basically consistent. Nevertheless, in the initial stages of the Dumore number ($N_{{u_{{{\text{st}}}} }}$) establishment, the transition zone between the displacing and displaced fluids was neglected. Thus, the artificial gas cap immiscible stable gas flooding number ($N_{{{\text{GOI}}}}$), as a new estimation model, has solid theoretical foundation, wide application range and reliable calculation results.

### Experimental validation

#### Experimental section

1. Experimental equipment

**Fig. 6:**
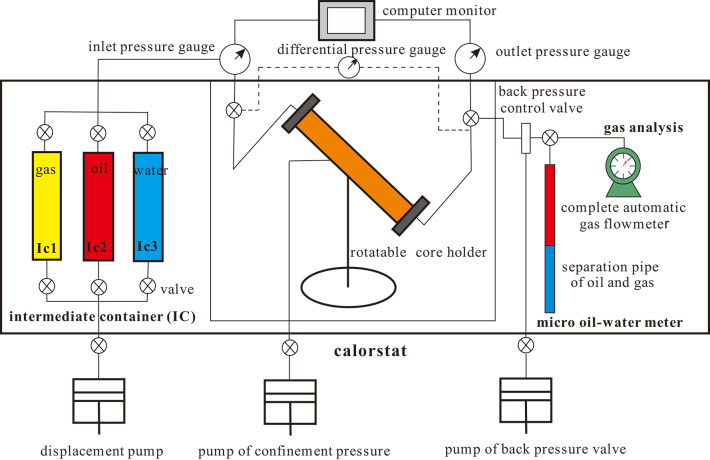
Sketch map of set-up of artificial CO_2_ gas cap immiscible rigid stable gas flooding.

As demonstrated in Fig. 6, the test equipment has been adapted from the formation damage evaluation system (CLDR-II) which was manufactured by Hubei Chuanglian Petroleum Technology Co., Ltd.. The equipments is equipped with a 1-inch rotatable core holder with an inner diameter of 25 mm and an internal length of 100 mm, which is capable of meeting the test conditions of different inclinations. In addition, the equipment is comprised of three high-temperature and high-pressure intermediate containers with a capacity of 1000 ml, which were respectively filled with oil, gas, and water prior to the commencement of the tests. The displacement pump, the confinement pressure pump, and the back pressure pump are Teledyne Isco 65 × piston pumps, which have a test precision of up to ± 0.00001 ml/min. It is evident that the flow rate of 00001 ml/min fulfils the requirements of the displacement, and moreover, it is able to satisfy the test conditions. The working pressure range of the test equipment is from 0 to 70 MPa, and the working temperature range is from 0 to 220 °C, which can meet the test temperature and pressure requirements. Throughout the test process, the following devices were employed: ① The utilisation of a constant temperature box is instrumental in establishing a constant temperature environment. ② The utilisation of a computer monitor the variations in displacement pressure and the alterations in oil and water production. ③ The utilisation of an complete automatic gas flowmeter is imperative for the precise measurement of output gas, which boasts a measurement accuracy of ± 0.01 ml and a working flow rate range of 0–150 ml/min, ensures precise and reliable measurement of output gas. ④ The utilisation of a micro oil–water meter tube constitutes a process that facilitates the measurement of oil and water production. The meter tube is fabricated from Pasmo carbon polymer, a special transparent material belonging to the ultra-high polymer amorphous polymer category. The meter tube boasts a temperature range of − 60 ℃ to 125 ℃, a transparency level of up to 90%, and a high level of mechanical strength. The meter tube is affixed with a transparent scale, and the posterior aspect of the meter tube is illuminated by an integrated light source. The front side of the meter tube is equipped with an electronic camera that is utilised for the observation of the minute movements of the gas–oil interface and of the oil–water interface within the meter tube. The metrological precision of the device has been demonstrated to be as precise as ± 0.0001 ml. ⑤ The utilisation of a high-pressure differential pressure sensor measure the pressure, with a testing accuracy of ± 0.0002 MPa. ⑥ The working pressure during the test is 0–69 MPa, while the working differential pressure is 0–1.5 MPa. It is evident that the aforementioned devices and their testing accuracies align with the experimental design requirements.

(2) Materials

The experiment utilised three plunger artificial cores, with a diameter of approximately 25 mm and a length of nearly 80 mm. The water employed for the experiments was standard brine, with the formula [NaCl:CaCL_2_:MgCl_2_·6H_2_O = 7:0.6:0.4 (mass ratio)] and a total mineralisation of 13,000 mg/l. The experimental oils were refined kerosene with a viscosity of 0.695 mPa s and a density of 0.7696 g/cm^3^, and 5# white Oil with a viscosity of 1.277 mPa s and a density of 0.7814 g/cm^3^ at 100 ℃ and 10 MPa. CO_2_ with a purity of 99.99% was sourced from Maoming Huayue Huayuan Gas Co., Ltd.. According to the NIST Chemistry WebBook, the CO_2_ viscosity was measured to be 0.0218 mPa s, while its density was determined to be 0.1886 g/cm^3^ at a temperature of 100 °C and a pressure of 10 MPa.

(3) Experimental design

In accordance with the assessment model ($N_{{{\text{GOI}}}}$) of the artificial CO_2_ gas cap immiscible rigid stable gas flooding, the experiments of strata dip, injection rate, permeability, oil density and viscosity were designed to analyse the influence of each factor on the artificial CO_2_ gas cap immiscible rigid stable gas flooding. The overarching objectives of this tests are twofold: firstly, to improve crude oil recovery, and secondly, to enhance CO_2_ sequestration. Additionally, the mechanism by which the displacement efficiency of the oil is improved will be investigated.

As illustrated in Table 6, the test conditions and schemes of the factors affecting the artificial CO_2_ gas cap immiscible rigid stable gas flooding are presented. Experiments #1 and #2 are designed to evaluate the influence of oil density and viscosity. Experiments #2 and #5 are intended to assess the impact of injection rate. Experiments #3 and #5 are focused on investigating the effect of air permeability. Experiments #4, #5 and #6 have been designed to evaluate the influence of strata dip.

**Table 6:** Experimental conditions and schemes of influencing factors on artificial gas cap immiscible rigid stable gas flooding under CO_2_ injection.

Test	#1	#2	#3	#4	#5	#6
Core	C1		C2	C3		
Diameter (cm)	2.475		2.482	2.478		
Length (cm)	7.764		7.842	7.911		
Porosity (%)	12.8		12.9	14.1		
Air permeability (mD)	0.609		5.25	1.53		
Oil saturation (%)	69.2	73.2	74.5	72.9	72.4	72.0
Strata dip(°)	60			30	60	90
Injection/displacement rate (m/d)	0.02408	0.02630	0.01107	0.01056		
Saturated media	5# White Oil	Refined Kerosene				
Oil density (g/cm^3^)	0.7814	0.7696				
Oil viscosity (mPa s)	1.277	0.695				
Experimental temperature (℃)	100					
Experimental pressure (MPa)	10					
Confinement pressure (MPa)	15					
Back pressure (MPa)	10

(4) Experimental procedure

The specific experimental steps are as follows: ① Wash the oil and salt of the rock core. ② Dry the core, followed by testing the porosity and air permeability of the core. ③ Vacuum the core, then apply pressure to saturate it with water. ④ Insert the core into the holder and connect the holder to the experimental system. ⑤ Increase the temperature and pressure to 100℃ and 10 MPa, respectively. ⑥ Saturate the core with the water and oil in the horizontal position of the holder, successively. Then, age the core for 24 h. It is imperative to pay close attention to the measurement of the produced liquit and the pressure during this period. ⑦ Adjust the dip of the core holder to the designed value of the strata dip, and test the permeability of the oil phase, initial water saturation and oil saturation under the condition of bound water, in accordance with the injection rate designed for the experiment. ⑧ Conduct the tests of the artificial CO_2_ gas cap immiscible rigid stable gas flooding. During these processes, the data and its variations of the inlet pressure, outlet pressure, duration, oil production, and gas production must be meticulously documented. The process is to be continued until the core exhibits no signs of oil production, at which point the experiment is to be brought to a close. ⑧ Change the variables of the experimental schemes in steps ① to ⑧ to conduct the next set of experiments.

#### Experimental results

As shown in Fig. 6 and Table 7, the experimental pressure, confining pressure and back pressure remained constant throughout the tests. Furthermore, under the set high-temperature and high-pressure conditions, the ratio of the initial CO_2_ volume in the intermediate container to the saturated oil volume in the core pores was as high as 254.5–302.1. This far exceeds the lower limit value of the gas cap index of ≥ 1.5 for reservoirs with sufficient gas cap driving energy^56–59^. Based on the definition of rigid gas drive, the development mode of these tests is considered to be artificial CO_2_ gas cap immiscible rigid gas flooding.

**Table 7:** Results of the strata dip, injection rate (or displacement rate), air permeability, oil density and oil viscosity experiments.

Test	#1	#2	#3	#4	#5	#6
Length (cm)	7.764		7.842	7.911		
Air permeability (mD)	0.609		5.25	1.53		
Oil saturation (%)	69.2	73.2	74.5	72.9	72.4	72.0
Strata dip (°)	60			30	60	90
Injection/displacement rate (m/d)	0.02408	0.02630	0.01107	0.01056		
Liquid phase relative permeability at the gas displacing oil front	0.5157	0.4666	0.5801	0.2025	0.4176	0.5902
Gas phase relative permeability at the gas displacing oil front	0.0197	0.0241	0.0171	0.0343	0.0175	0.0183
Oil density (g/cm^3^)	0.7814	0.7696				
Oil viscosity (mPa s)	1.277	0.695				
Displacement efficiency before gas breakthrough occurs (%)	17.57	22.69	42.39	27.67	32.91	50.58
displacement multiple before gas breakthrough occurs (PV)	0.122	0.166	0.318	0.202	0.237	0.388
CO_2_ storage volume before gas breakthrough occurs (PV)	0.122	0.166	0.318	0.202	0.237	0.388
The proportion of the cumulative oil production before gas breakthrough occurs to the total cumulative oil production(%)	32.70	39.82	60.97	45.55	48.26	70.22
Displacement efficiency at the end of the experiment (%)	53.72	56.98	70.01	60.75	67.69	72.03
displacement multiple at the end of the experiment (PV)	16.083	15.936	54.781	68.351	55.531	42.410
CO_2_ storage volume at the end of the experiment (PV)	0.372	0.417	0.522	0.443	0.490	0.519
The proportion of CO_2_ storage volume before gas breakthrough occurs to the total storage volume (%)	32.80	39.80	60.92	45.60	48.37	74.76
$N_{{u_{{{\text{st}}}} }}$	0.122	0.122	4.99	1.52		
$N_{{{\text{GOI}}}}$	0.020	0.040	1.042	0.005	0.058	2.044

The determination of the gas breakthrough point is achieved through the analysis of the alterations in the displacement efficiency and the gas–oil ratio with the displacement multiple, which can also reflect the size of the injected gas volume. As demonstrated in Fig. 8 (#1), upon increasing the volume of the injected fluid (displacement multiple) to 0.122 PV, there is a marked rise in the gas–oil ratio, accompanied by a shift in the oil displacement efficiency from a rapid to a slow increase. This phenomenon can be attributed to the instability of the gas displacing oil front (i.e., gas–oil interface) and the gas breakthrough. It is possible to obtain the gas breakthrough point of the experiments by observing the changes in the displacement efficiency or the gas–oil ratio with the gas injection section plug during the test processes.

Krg is the relative permeability of CO_2_ gas, Krog is the oil-CO_2_ gas mixture phases permeability relative to bound water-CO_2_ gas mixture phases, f_g_ is the fraction of CO_2_ gas in flowing stream, and* S*_l_ is the bound water-CO_2_ gas mixture phases saturation.

**Fig. 7:**
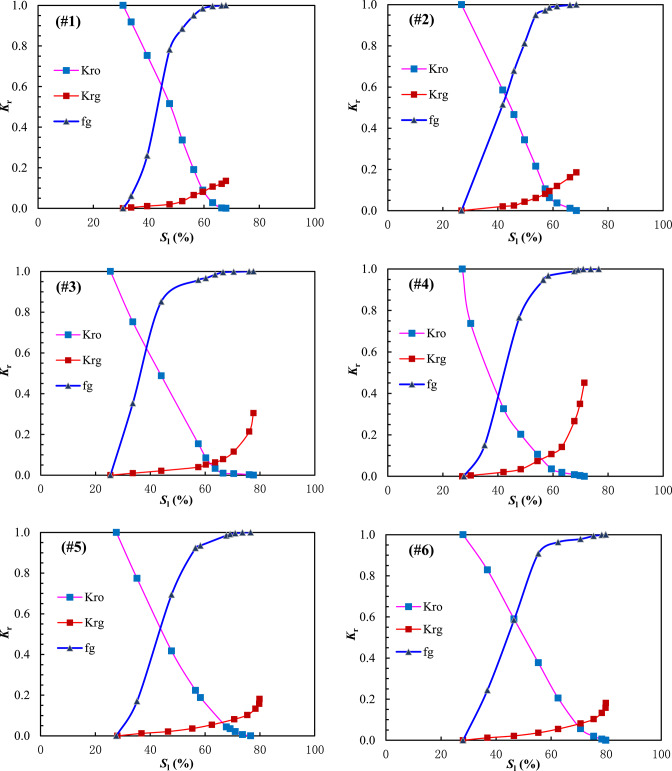
The gas–liquid relative permeability (Kr) curves of the experiments #1, #2, #3, #4, #5, and #6.

**Fig. 8:**
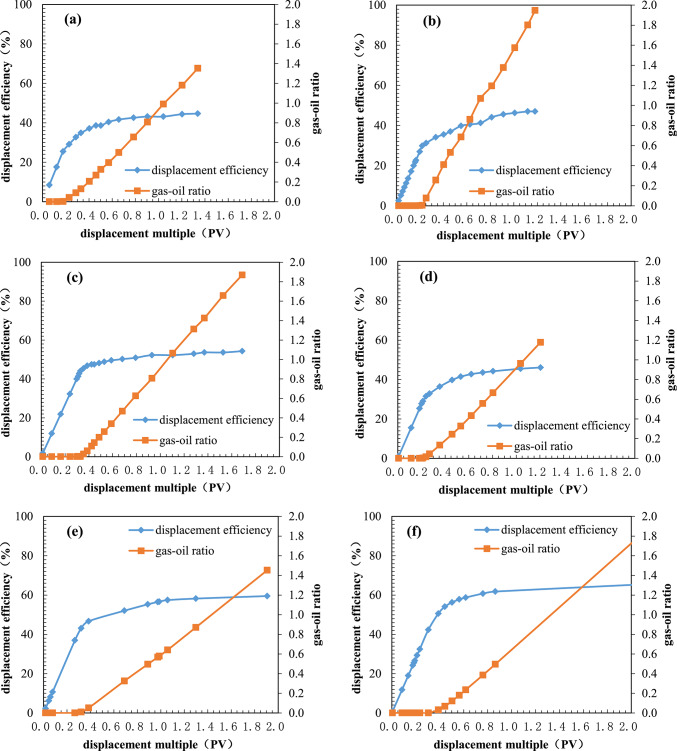
Variation of displacement efficiency and gas–oilratio with displacement multiple (i.e., the ratio of the volume of injected CO_2_ to the volume of the core pores) in the experiments #1, #2, #3, #4, #5, and #6. Figures (**a**), (**b**), (**c**), (**d**), (**e**) and (**f**) respectively show the variation curves of the displacement efficiency and the gas–oil ratio with the displacement multiple in experiments #1, #2, #3, #4, #5, and #6.

As shown in Figs. 7, 8 and Table 7, From the initiation of gas flooding until the gas breakthrough, there is a sharp increase in the displacement efficiency, the gas–oil ratio remains stable, and CO_2_ is efficiently and stably displaced for oil and stored with high quality. Subsequent to the gas breakthrough, there is a marked increase in the displacement efficiency, a sharp rise in the gas–oil ratio, and the effect of the oil displacement and storage of CO_2_ is relatively poor. The gas breakthrough points of Experiments #1, #2, #3, #4 , #5 and #6 were 0.122 PV, 0.166 PV, 0.318 PV, 0.202 PV, 0.237 PV and 0.388 PV respectively. The proportion of the cumulative oil production before gas breakthrough occurs to the total cumulative oil production accounted for approximately 32.70%, 39.82%, 60.97%, 45.55%, 48.26%, and 70.22%, reflecting that the stable production stage (i.e., the production stage before gas breakthrough occurs) has a significant impact on improving the oil displacement efficiency. Experiments #1, #2, #4 and #5 of artificial CO_2_ gas cap immiscible rigid gas flooding broke through prematurely. The duration of the stable production stage was brief, and only partial stable gas flooding was achieved. The proportion of the cumulative oil production before gas breakthrough occurs to the total cumulative oil production were low, with all values falling below 50%. Furthermore, the overall displacement efficiency were also found to be relatively low, with all values falling below 33%. The majority of oil production took place during the unstable production stage (i.e., the production stage during the gas breakthrough exists). The development of a gas drive for artificial CO_2_ gas tops that is both non-mixed-phase and rigid has not yet been achieved. So Experiments #1, #2, #4 and #5 failed to achieve stable gas flooding due to premature gas breakthrough. Before the gas breakthrough occurred, the displacement multiple, displacement efficiency, and CO_2_ storage volume were found to range from 0.122 to 0.237 PV, with an average of 0.182 PV; from 17.57% to 32.91%, with an average of 25.21%; and from 0.122 to 0.237 PV, with an average of 0.182 PV, respectively. The earlier the gas breakthrough occurs, the lower the displacement efficiency, the cumulative oil production, and the CO_2_ storage volume, resulting in a poorer oil recovery and CO_2_ storage effect. Conversely, in Experiments #3 and #6, the gas breakthrough times of the artificial CO_2_ gas cap immiscible rigid gas flooding were observed to be delayed, the stable production stage were found to be prolonged, and the proportion of the cumulative oil production prior to gas breakthrough to the total cumulative oil production were found to exceed 60%. The displacement efficiency levels were found to be notably high, with values exceeding 40%. It is evident that a viable and reliable production stage has been established within the stable gas flooding phase. The majority of oil production was successfully extracted during these stable period. thereby facilitating the development of artificial CO_2_ gas cap non-miscible rigid stable gas flooding. It is obvious that Experiments #3 and #6 achieved the development of artificial CO_2_ gas cap immiscible rigid stable gas flooding. Prior to gas breakthrough, the displacement multiple, displacement efficiency, and CO_2_ storage volume were found to range from 0.318 to 0.388 PV, with an average of 0.353 PV; from 42.39% to 50.58%, with an average of 46.49%; and from 0.318 to 0.388 PV, with an average of 0.353 PV, respectively. The later the gas breakthrough occurs, the greater the displacement efficiency, and CO_2_ storage volume, the more oil cumulated. This improves the oil recovery and CO_2_ storage effect.

As shown in Table 7, the calculation results of Dumore number ($N_{{u_{{{\text{st}}}} }}$) derived from the Dumore criterion rate indicate that Experiments #1 and #2 failed to achieve stable gas flooding. In contrast, Experiments #3 to #6 all achieved stable gas flooding. However, of these, only the calculation results of Dumore number in Experiments #1, #2, #3 and #6 were consistent with the experimental results. The application results suggest that Dumore number is not very adaptable. $N_{{{\text{GOI}}}}$ values of Experiments #1, #2, #4 and #5 are 0.020, 0.040, 0.005 and 0.058, respectively. All of these values are less than 1.0, meaning that stable gas flooding cannot be achieved. In contrast, $N_{{{\text{GOI}}}}$ values of Experiments #3 and #6 are 1.045 and 2.044, respectively, and can therefore achieve stable gas flooding. The calculation values are consistent with the experimental results and understanding. As $N_{{{\text{GOI}}}}$ increases, an artificial CO_2_ gas cap immiscible rigid gas flooding can either fully or partially achieve stable gas flooding. This delays the gas breakthrough time, improves the oil recovery effect and strengthens the CO_2_ sequestration capacity.

Through comparative analysis, it is believed that $N_{{{\text{GOI}}}}$ can effectively and simply evaluate the degree of stability of the gas displacing oil front of the crestal gas injection for immiscible stable gas flooding, and that the judgement results are accurate and reasonable. This provides a theoretical model for studying the mechanism of crestal gas injection for immiscible gas flooding and also provides a certain decision-making basis for the selection of applicable reservoirs and on-site construction of the crestal gas injection for immiscible stable gas flooding, especially artificial CO_2_ gas cap immiscible rigid gas flooding.

## Disscussion

### Influencing factors of $N_{{{\text{GOI}}}}$

#### Analysis of influencing factors

As demonstrated by Eq. (31) and the process by which it was derived, it can be discerned that the factors affecting the artificial gas cap immiscible rigid stable gas flooding number (or artificial gas cap immiscible stable gas flooding number) ($N_{{{\text{GOI}}}}$) (or artificial gas cap immiscible rigid stable gas flooding, or artificial gas cap immiscible stable gas flooding) can be categorized as follows: crude oil density, crude oil viscosity, gas density, gas viscosity, gas injection rate (or displacement rate, or gas cutting rate) under the formation conditions, strata dip, relative permeability and air permeability in the stratigraphic direction. As shown in Tables 2, 3 and 8, in order to elucidate the principal factors influencing $N_{{{\text{GOI}}}}$ and the artificial gas cap immiscible stable gas flooding, the average geological and development data values related to the West Hackberry Oilfield、the pilot area of Yanling Oilfield and the fault block reservoirs in central and eastern China following water flooding development were used as the basic parameters to analyse the impact of each factor on $N_{{{\text{GOI}}}}$ and the process of the artificial gas cap immiscible stable gas flooding under gas injection, as well as the EOR and CO_2_ storage mechanism of the artificial gas cap immiscible stable gas flooding under gas injection.

**Table 8:** Parameters of the complex fault block reservoirs in central and eastern China following water flooding development^8,26,53–59^.

Property	Range of value	AV. value	Descr
Injection gas type	CO_2_	–	
Oil density (g/cm^3^)	0.75–0.94	0.86	
Oil viscosity (mPa s)	1.3–150.0	28	
Gas density (g/cm^3^)	0.5–0.95	0.75	Data from the CO_2_ injection projects that have been implemented. No artificial gas cap flooding project operation
Gas viscosity (mPa s)	0.03–0.12	0.045	
Displacement rate (m/d)	0.005–0.3	0.12	
Strata dip (°)	5–70	30	
Air permeability at the stratigraphic direction	0.1–5000	500	
Liquid phase relative permeability	0.3–0.72	0.5	At the gas displacing oil front (i.e., gas–oil interface)
Gas phase relative permeability	0.001–0.052	0.018

1. Influence of crude oil density on the effectiveness of oil displacement and CO_2_ storage

Figure 8a, b, and Table 7 illustrate that the results of Experiments #1 and #2 are significantly influenced by numerous factors, including gas injection rate, liquid phase relative permeability, gas phase relative permeability, and oil viscosity. It is evident that the variation relationship of crude oil density and $N_{{{\text{GOI}}}}$ does not align with conventional expectations.

**Fig. 9:**
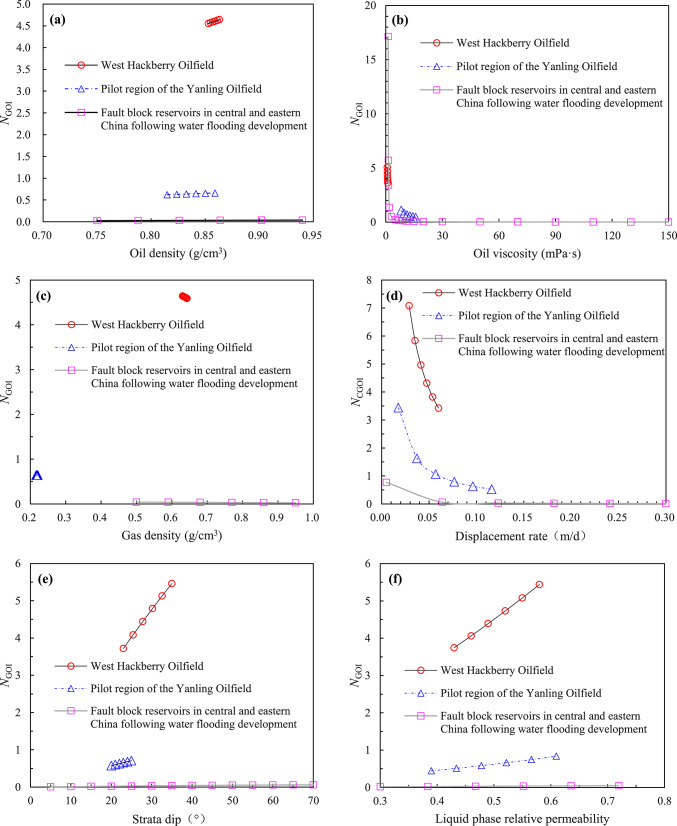

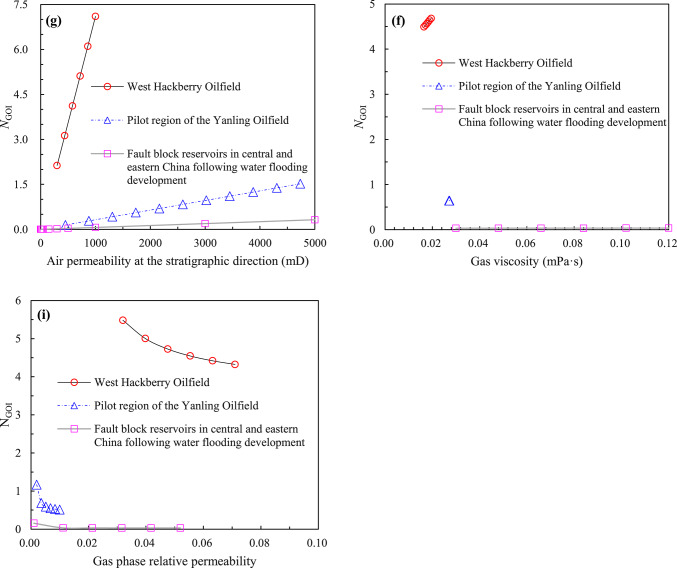
Relationship curves of $N_{{{\text{GOI}}}}$ versus the oil density (**a**), the oil viscosity (**b**), the gas density (**c**), the displacement rate i.e. the gas injection rate, or the gas cutting rate (**d**, the strata dip (**e**), the liquid phase relative permeability (**f**, the air permeability at the stratigraphic direction (**g**), the gas viscosity (**h**), the gas phase relative permeability (**i**).

Figure 9(a) and Table 9 shows under conditions of constant other influencing factors, a linear relationship exists between the crude oil density and $N_{{{\text{GOI}}}}$. Furthermore, an increase in the crude oil density is accompanied by a concomitant rise in the value of $N_{{{\text{GOI}}}}$, thus leading to a more stable front of gas displacing oil and the effect of oil displacement and CO_2_ storage will also be better. Due to differences in the geological and development parameters of the different reservoirs, the magnitude of the change in $N_{{{\text{GOI}}}}$ varies despite the uniformity of the crude oil density and its fluctuations. The $N_{{{\text{GOI}}}}$ values of the pilot region of the Yanling Oilfield and the fault block reservoirs in central and eastern China following water flooding development are both less than 1.0, indicating that the gas displacing oil front is unstable and gas breakthrough occurs, as they are severely affected by the other geological and development parameters of the reservoir. However, as the crude oil density increases, the stability of the gas displacing front gradually improves. All the $N_{{{\text{GOI}}}}$ values of the West Hackberry Oilfield are greater than 4.5, indicating a stable gas displacing front. As the density of the crude oil increases, the stability of the gas displacing front also improves.

**Table 9:** Functional relationship model of $N_{{{\text{GOI}}}}$ versus the influencing factors including the oil density, the oil viscosity, the gas Density, the displacement rate (i.e. the gas injection rate, or the gas cutting rate), the strata dip, the liquid phase relative permeability, the air permeability at the stratigraphic direction, the flue gas Viscosity, the gas phase relative permeability.

Influencing factors	Functional relationship model		
	West Hackberry Oilfield	Pilot region of the Yanling Oilfield	Fault block reservoirs in central and eastern China following water flooding development
Oil density	$$N_{{{\text{GOI}}}} = 8.5201\rho_{{\text{o}}} - 2.7128\begin{array}{*{20}c} {} & {} \\ \end{array} R^{2} { = }1$$	$$N_{{{\text{GOI}}}} = 0.8822\rho_{{\text{o}}} - 0.0966\begin{array}{*{20}c} {} & {} \\ \end{array} R^{2} { = }1$$	$$N_{{{\text{GOI}}}} = 0.066\rho_{{\text{o}}} - 0.0248\begin{array}{*{20}c} {} & {} \\ \end{array} R^{2} { = }1$$
Oil viscosity	$$N_{{{\text{GOI}}}} = 4.5273\mu_{{\text{o}}}^{ - 1.424} \begin{array}{*{20}c} {} & {} \\ \end{array} R^{2} { = 0}.937$$	$$N_{{{\text{GOI}}}} = 4.0563\mu_{{\text{o}}}^{ - 1.234} \begin{array}{*{20}c} {} & {} \\ \end{array} R^{2} { = }0.9999$$	$$N_{{{\text{GOI}}}} = 21.335\mu_{{\text{o}}}^{ - 1.438} \begin{array}{*{20}c} {} & {} \\ \end{array} R^{2} { = }0.9982$$
Gas density	$$N_{{{\text{GOI}}}} = - 4.2601\rho_{{\text{g}}} + 7.329\begin{array}{*{20}c} {} & {} \\ \end{array} R^{2} { = }1$$	$$N_{{{\text{GOI}}}} = - 0.4411\rho_{{\text{g}}} + 0.7383\begin{array}{*{20}c} {} & {} \\ \end{array} R^{2} { = }1$$	$$N_{{{\text{GOI}}}} = - 0.033\rho_{{\text{g}}} + 0.0568\begin{array}{*{20}c} {} & {} \\ \end{array} R^{2} { = }1$$
Gas viscosity	$$N_{{{\text{GOI}}}} = 62.177\mu_{{\text{g}}} + 3.4666\begin{array}{*{20}c} {} & {} \\ \end{array} R^{2} { = }0.9999$$	$$N_{{{\text{GOI}}}} = 10.093\mu_{{\text{g}}} + 0.3668\begin{array}{*{20}c} {} & {} \\ \end{array} R^{2} { = }1$$	$$N_{{{\text{GOI}}}} = 0.0355\mu_{{\text{g}}} + 0.0304\begin{array}{*{20}c} {} & {} \\ \end{array} R^{2} { = }9992$$
Displacement rate	$$N_{{{\text{GOI}}}} = 0.2054v_{{\text{t}}}^{ - 1} \begin{array}{*{20}c} {} & {} \\ \end{array} R^{2} { = }1$$	$$N_{{{\text{GOI}}}} = 0.0608v_{{\text{t}}}^{ - 1} \begin{array}{*{20}c} {} & {} \\ \end{array} R^{2} { = }1$$	$$N_{{{\text{GOI}}}} = 0.0038v_{{\text{t}}}^{ - 1} \begin{array}{*{20}c} {} & {} \\ \end{array} R^{2} { = }1$$
Strata dip	$$N_{{{\text{GOI}}}} = 0.1451\alpha + 0.3955\begin{array}{*{20}c} {} & {} \\ \end{array} R^{2} { = }1$$	$$N_{{{\text{GOI}}}} = 0.027\alpha + 0.0332\begin{array}{*{20}c} {} & {} \\ \end{array} R^{2} { = }1$$	$$N_{{{\text{GOI}}}} = 0.0009\alpha + 0.0046\begin{array}{*{20}c} {} & {} \\ \end{array} R^{2} { = }1$$
Air permeability at the stratigraphic direction	$$N_{{{\text{GOI}}}} = 0.0071k_{\angle } \begin{array}{*{20}c} {} & {} \\ \end{array} R^{2} { = }1$$	$$N_{{{\text{GOI}}}} = 0.0003k_{\angle } \begin{array}{*{20}c} {} & {} \\ \end{array} R^{2} { = }1$$	$$N_{{{\text{GOI}}}} = 0.00006k_{\angle } \begin{array}{*{20}c} {} & {} \\ \end{array} R^{2} { = }1$$
Liquid phase relative permeability	$$N_{{{\text{GOI}}}} = 11.294K_{{{\text{rog}}\angle }} - 1.1289\begin{array}{*{20}c} {} & {} \\ \end{array} R^{2} { = }0.9995$$	$$N_{{{\text{GOI}}}} = 1.7796K_{{{\text{rog}}\angle }} - 0.2575\begin{array}{*{20}c} {} & {} \\ \end{array} R^{2} { = }0.9971$$	$$N_{{{\text{GOI}}}} = 0.0672K_{{{\text{rog}}\angle }} - 0.0014\begin{array}{*{20}c} {} & {} \\ \end{array} R^{2} { = }0.9999$$
Gas phase relative permeability	$$N_{{{\text{GOI}}}} = 1.9566K_{{{\text{rg}}\angle }}^{ - 0.294} \begin{array}{*{20}c} {} & {} \\ \end{array} R^{2} { = }0.9776$$	$$N_{{{\text{GOI}}}} = 0.048K_{{{\text{rg}}\angle }}^{ - 0.494} \begin{array}{*{20}c} {} & {} \\ \end{array} R^{2} { = }8991$$	$$N_{{{\text{GOI}}}} = 0.0073K_{{{\text{rg}}\angle }}^{ - 0.419} \begin{array}{*{20}c} {} & {} \\ \end{array} R^{2} { = 0}.8835$$

The research suggests that as the crude oil density increases, the buoyancy gradient of the injected gas, gravity gradient of the crude oil density and capillary force gradient also increase. The effect of gravitational differentiation and capillary force becomes more pronounced, causing the crude oil to sink while the injected gas floats up. It is probable that the injected gas will move to the top region of the reservoir or the upper part of the inclined structure, thus forming an gas cap. This will, in turn, facilitate the effective replacement of the attic oil in the top region of the reservoir or the upper part of the inclined structure. The primary benefits of this approach include the suppression of viscous fingering, the maintenance of the stability of the gas displacing oil front, the effective replacement of the bypass oil in the water-flooded area, the increase in efficient and stable production time, the improvement of displacement efficiency and sweep efficiency, and the enhancement of the potential for CO_2_ sequestration. As demonstrated in the assessment model ($N_{{{\text{GOI}}}}$) of the artificial gas cap immiscible rigid stable gas flooding, an increase in the crude oil density is accompanied by an increase in the $N_{{{\text{GOI}}}}$ value. The efficient and stable production development time is prolonged, and the effect of oil displacement and CO_2_ storage is enhanced. It can thus be concluded that an increase in the crude oil density is beneficial for the development of the artificial CO_2_ gas cap immiscible stable gas flooding.

(2) Influence of crude oil viscosity on the effectiveness of oil displacement and CO_2_ storage

As demonstrated in Fig. 8a, b and Table 7, it is evident that that although the results of Experiments #1 and #2 are affected by various factors such as gas injection rate, liquid phase relative permeability, gas phase relative permeability, and crude oil oil density, the relationship between crude oil viscosity and $N_{{{\text{GOI}}}}$ is discernible. Moreover, the greater the crude oil viscosity, the smaller $N_{{{\text{GOI}}}}$ is, and the worse the stability of the gas displacing oil front. The experiment with a higher viscosity whose oil viscosity is 1.277 mPa s resulted in an injected gas volume of 0.122 PV before the gas breakthrough occurs, and the final displacement efficiency was 53.72%, with the final CO_2_ sequestration volume being 0.372 PV. The displacement efficiency and CO_2_ sequestration volume of the experiment with the higher oil viscosity were increased by 3.26% and 0.045 PV respectively compared to those of the experiment with the lower oil viscosity whose oil viscosity is 0.695 mPa s.

Figure 9(b) and Table 9 shows a power function relationship exists between crude oil viscosity and $N_{{{\text{GOI}}}}$_._ It can be deduced that an increase in the crude oil viscosity results in a decrease in $N_{{{\text{GOI}}}}$, thereby leading to a deterioration in the stability of the gas displacing oil front. The $N_{{{\text{GOI}}}}$ values of the West Hackberry Oilfield, the pilot region of the Yanling Oilfield and the fault block reservoirs in central and eastern China following water flooding development are all distributed within the slow-declining regions of the power function curves. The crude oil viscosity of the West Hackberry Oilfield ranges from 0.8382 to 1.0964 mPa s and the corresponding $N_{{{\text{GOI}}}}$ values are all greater than 3.6, which indicates a stable gas displacing oil front. However, as the crude oil viscosity increases, the stability of the gas displacing oil front gradually deteriorates. When the crude oil viscosity of the pilot region of the Yanling Oilfield and the fault block reservoirs in central and eastern China following water flooding development is less than approximately 8.5 mPa s and 2.1 mPa s, respectively, the corresponding $N_{{{\text{GOI}}}}$ values are greater than 1.0, which indicate a stable gas displacing oil front. As the crude oil viscosity decreases, the stability of the gas displacing oil front improves. When the crude oil viscosity of the pilot region of the Yanling Oilfield and the fault block reservoirs in central and eastern China following water flooding development is greater than 8.5 and 2.1 mPa s, respectively, the corresponding $N_{{{\text{GOI}}}}$ values are less than 1.0, indicating an unstable gas displacing oil front with gas breakthrough. It can be demonstrated that the utilisation of an artificial CO_2_ gas cap immiscible stable gas flooding is more appropriate for low-viscosity reservoirs. In circumstances where the crude oil viscosity is minimal, the gas breakthrough time is postponed and the stable displacement time is lengthened. Consequently, the recovery and CO_2_ sequestration effects are enhanced.

If the viscosity difference between injected gas and crude oil is significant, gas flooding processes are susceptible to form viscous fingering phenomena, which can result in instability of the gas displacing oil front and a consequent reduction in sweep volume and displacement efficiency. It has been demonstrated that this is not conducive to enhancing the crude oil recovery or facilitating large-scale CO_2_ storage. A reduction in the crude oil viscosity has been shown to decrease the mobility ratio, thereby effectively inhibiting viscous fingering and stabilizing the gas displacing oil front. Furthermore, this reduction in viscosity has been demonstrated to reduce the time for gas breakthrough, extend the period of gas flooding with high efficiency and stability, and exploit the attic oil and the bypass oil in waterflooded area. Consequently, these factors contribute to an improvement in the oil recovery and the potential for CO_2_ storage.

(3) Influence of injected gas density on the effectiveness of oil displacement and CO_2_ storage

As demonstrated in Fig. 9c and Table 9, the injected gas density exhibits a direct correlation with $N_{{{\text{GOI}}}}$ , and as the injected gas density increases, the value of $N_{{{\text{GOI}}}}$ experiences a continuous decrease, thereby leading to a deterioration in the stability of the gas displacing oil front. Due to differences in the geological and development parameters of the different reservoirs, the magnitude of the change in $N_{{{\text{GOI}}}}$ varies despite the uniformity of the injected gas density and its fluctuations. The influence of other geological and developmental parameters of the reservoirs is also worthy of note. Despite the low N_2_ density in the pilot region of the Yanling Oilfield and the wide distribution of the CO_2_ density in the fault block reservoirs in central and eastern China following water flooding development, their corresponding $N_{{{\text{GOI}}}}$ values are all less than 1.0. This results in an unstable gas displacing oil front and a gas breakthrough. However, as the injected gas density decreases, the stability of the gas displacing oil front gradually improves. For the West Hackberry Oilfield, the range of flue gas density is from 0.6304 to 0.6432 g/cm^3^, with the $N_{{{\text{GOI}}}}$ values all greater than 4.5, indicating a stable gas displacing oil front. However, as the flue gas density increases, the stability of the gas displacing oil front gradually deteriorates. It can be demonstrated that the artificial CO_2_ gas cap immiscible stable gas flooding is appropriate for low-density gases. In circumstances where the injected gas density is minimal, the gas is able to be displaced in a stable manner. This, in turn, serves to reduce the likelihood of the phenomenon known as gas breakthrough. Consequently, the effect of gas displacing oil and CO_2_ sequestration are deemed to be beneficial.

The injected gas exhibits a low density, and there is a substantial difference in density between the injected gas and the crude oil. The gravitational gradient acting on the injected gas is negligible, while the capillary force gradient increases. The gravitational differentiation effect is prominent, which is conducive to the upward movement of the injected gas towards the top of the reservoir or the upper part of the inclined structure, generating a secondary gas cap, allowing the attic oil to be exploited. The primary benefits of this approach include the suppression of viscous fingering, the maintenance of the stability of the gas displacing oil front, the augmentation of the time for efficient and stable production, the exploitation of the bypass oil, and the improvement of the displacement efficiency, sweep efficiency, oil recovery of gas flooding, and the potential for CO_2_ sequestration.

(4) Influence of gas injection rate on the effectiveness of oil displacement and CO_2_ storage

As illustrated in Fig. 8b, e and Table 7, it can be observed that the air permeability and gas injection rate of Experiments #5 are approximately 2.512 times and $\frac{1}{2.491}$ times those of Experiments #2, respectively. The calculation indicates that the discrepancy in $N_{{{\text{GOI}}}}$ value between Experiments #2 and Experiments #5 at this juncture is solely attributable to the liquid phase relative permeability, gas phase relative permeability and gas injection rate. Despite the results of Experiments #2 and #5 influenced by the liquid phase and gas phase relative permeability, the variation law between gas injection rate and $N_{{{\text{GOI}}}}$ is clear. It is evident that a decrease in the gas injection rate corresponds to an increase in $N_{{{\text{GOI}}}}$, thereby enhancing the stability of the gas displacing oil front. In the instance of a slow gas injection rate of 0.01056 m/d, the injected gas volume before the gas breakthrough occurs is 0.237 PV, the ultimate displacement efficiency is 67.59%, and the ultimate CO_2_ sequestration volume is 0.490 PV. Conversely, in the event of a fast gas injection rate of 0.02630 m/d, the injected gas volume before the gas breakthrough occurs is 0.166 PV, the ultimate displacement efficiency is 56.98%, and the ultimate CO_2_ sequestration volume is 0.417 PV. When the gas injection rate is low, the time of gas breakthrough is delayed, and the stable production period is prolonged, with improved development effect and CO_2_ sequestration.

Figure 9d and Table 9 shows that there is a power function relationship between gas injection rate and $N_{{{\text{GOI}}}}$. Moreover, as the injection rate increases, the $N_{{{\text{GOI}}}}$ value decreases, thereby leading to a deterioration in the stability of the gas displacing oil front. The distribution of $N_{{{\text{GOI}}}}$ in the West Hackberry Oilfield is observed to occur within the steeply declining segment of the power function curve. In contrast, the distribution of $N_{{{\text{GOI}}}}$ in the pilot region of the Yanling Oilfield is seen to extend across both the steeply and slowly declining segments of the power function curve. Finally, the distribution of $N_{{{\text{GOI}}}}$ in the fault block reservoirs in central and eastern China following water flooding development is confined to the slowly declining section of the power function curve. Among these, the $N_{{{\text{GOI}}}}$ values in the West Hackberry Oilfield are all greater than 3.4, the gas displacing oil front is stable, and as the gas injection rate decreases, the gas displacing oil front becomes more stable. The $N_{{{\text{GOI}}}}$ values in the fault block reservoirs in central and eastern China following water flooding development are all less than 1.0 due to the significant influence of other geological and development parameters. The gas displacing oil front is unstable, the gas breakthrough occurs, and as the gas injection rate decreases, the stability of the gas displacing oil front gradually improves. It has been demonstrated that when the gas injection rate in the pilot region of the Yanling Oilfield is approximately less than 0.0608 m/d, the $N_{{{\text{GOI}}}}$ value is greater than 1.0. In such cases, the gas displacing oil front is stable. Furthermore, as the gas injection rate decreases, the stability of the gas displacing oil front is further improved. Conversely, when the injection rate in the pilot region of the Yanling Oilfield exceeds 0.0608 m/d, the $N_{{{\text{GOI}}}}$ value is less than 1.0. This results in an unstable gas displacing oil front and a gas breakthrough. Finally, as the injection rate increases, the stability of the gas displacing oil front deteriorates.

The effectiveness of artificial gas cap immiscible stable gas flooding is contingent upon the stability of the gas displacing oil front. It is evident that an elevated gas injection rate will yield a reduced $N_{{{\text{GOI}}}}$ value, concomitant with an augmentation in the gas injection rate at the gas displacing oil front. This phenomenon is accompanied by the instability of the gas displacing oil front (i.e., the gas–oil interface), in addition to an intensification of the viscous fingering phenomenon. The consequence of these phenomena is premature gas breakthrough. Conversely, immiscible stable gas flooding is predicated on the gas–oil capillary force and the gravitational differentiation effect engendered by the density difference of oil and gas, culminating in a distribution state of gas–oil–water from top to bottom in the reservoir, thereby suppressing the viscous fingering. In the event of the injection rate being excessively rapid, the gravitational differentiation effect proves to be inadequate and overdue, thus exerting an adverse effect on the oil displacement and CO_2_ sequestration during the stable gas flooding. Conversely, if the gas injection rate is low, the $N_{{{\text{GOI}}}}$ value is large, and the gas injection rate at the gas displacing oil front decreases. This is beneficial for maintaining the stability of the gas displacing oil front (i.e., the gas–oil interface), and inhibiting viscous fingering and exploiting the bypass oil in the water-flooded area. Consequently, the ultimate oil recovery and CO_2_ storage capacity is enhanced. This observation does not infer that a reduced gas injection rate will necessarily result in enhanced oil recovery and CO_2_ storage. In the event of an insufficient gas injection rate, there will be a substantial prolongation of the production period and an increase in operational costs, thus failing to meet the requirements of the oilfield production plan. When considering both economic benefits and developmental effects in their totality, it can be posited that there exists an optimal gas injection rate.

(5) Influence of strata dip on the effectiveness of oil displacement and CO_2_ storage

As demonstrated in Fig. 8d, e, f and Table 7, although the results of Experiments #4, #5 and #6 are influenced by the relative permeability of the liquid and gas phases, the correlation between strata dip angle and $N_{{{\text{GOI}}}}$ is evident. However, the variation in the strata dip and $N_{{{\text{GOI}}}}$ is discernible. It has been illustrated that, in the case of larger strata dips, greater values of $N_{{{\text{GOI}}}}$ are observed, and the stability of the gas displacing oil front is enhanced. As the strata dip increases, the gas breakthrough time is delayed, and the gas breakthrough points at the strata dips of 30°, 60°, and 90° are 0.202 PV, 0.237 PV, and 0.388 PV, respectively. The efficient and stable production time is prolonged, and the ultimate displacement efficiency is increased. The sample with a strata dip of 90° has an increase of 11.28% compared to 30°, and the ultimate CO_2_ sequestration volume increases by 0.076 PV compared to 30°. From the start of gas flooding to the point of gas breakthrough, the displacement efficiency rises sharply, and efficient and stable production occurs. After the gas breakthrough, the increase in displacement efficiency slows down, and production enters a stable period. This reflects the existence of an efficient and stable production stage before gas breakthrough during artificial gas cap immiscible stable gas flooding. In Experiments #4, #5 and #6, the proportions of the cumulative oil production before gas breakthrough to the total cumulative oil production were found to be approximately 45.55%, 48.26%, and 70.22%, respectively. Similarly, the proportions of CO_2_ storage volume before gas breakthrough to the total storage volume accounted for 45.60%, 48.37%, and 74.76%, respectively. This reveals that the stable production stage has a significant impact on improving oil recovery and the potential of CO_2_ sequestration.

As illustrated in Fig. 9e and Table 9, the strata dip has been shown to be linearly related to A; as the strata dip increases, the value of $N_{{{\text{GOI}}}}$ increases monotonically, and the gas displacing oil front (i.e., the gas–oil interface) becomes increasingly stable. It is evident that disparities in geological and developmental parameters among diverse reservoirs give rise to variations in $N_{{{\text{GOI}}}}$, despite the uniformity of the strata dip and its fluctuations. The gas displacing oil front is unstable due to the significant influence of other geological and development parameters of the reservoir, despite the fact that the $N_{{{\text{GOI}}}}$ values in the pilot region of the Yanling Oilfield and the fault block reservoirs in central and eastern China following water flooding development are both less than 1.0. Consequently, the gas breakthrough occurs. However, as the strata dip increases, the stability of the gas displacing oil front gradually improves. The $N_{{{\text{GOI}}}}$ values in the West Hackberry Oilfield are all greater than 3.7, and the gas displacing oil front is stable. As the strata dip increases, the stability of the gas displacing oil front is known to improve further.

An increase in the strata dip has been shown to be beneficial for the development of the artificial gas cap immiscible stable gas flooding. It has been demonstrated that an increase in the strata dip results in enhanced the stability of the gas displacing oil front, prolonged stable production time, elevated oil recovery, and augmented CO_2_ storage volume. The following reasons have been posited for these phenomena: Firstly, it is evident that the effect of capillary force and gravitational differentiation is pronounced in the presence of a strata dip in reservoirs. The process of gas injection development of oil reservoirs is accompanied by the sinking of the crude oil, while the injected gas floats up, readily attaining the top position within the reservoir or the upper segment of the inclined structure, thereby generating a second gas cap. This facilitates the exploitation of the attic oil. Second, the presence of a density difference between oil and gas gives rise to the inhibition of viscous fingering, ensuring the stability of the gas displacing oil front. Concurrently, the residual oil accumulates at the gas displacing oil front, giving rise to the formation of an oil wall. It continuously advances towards the bottom in the reservoirs, promoting the efficient exploitation of the bypass oil in waterflooded area. Thirdly, when the oil wall (i.e., residual oil enrichment area) advances to the bottom outlet of the physical simulation model or the bottom of the oil production well, the rate of the oil production is high, and the stable displacement time is long. The swept volume, displacement efficiency, CO_2_ storage volume have been significantly improved in comparison with gas injection development in low strata dip reservoirs. In the end, As the strata dip is increased, the gravitational gradient of crude oil, the buoyancy gradient of injected gas, and the capillary force gradient also increase. This will enhance the effect of capillary force and gravitational differentiation, facilitate the exertion of capillary force effect, inhibit viscous fingering, and achieve stable gas flooding. According to the assessment model ($N_{{{\text{GOI}}}}$) for the artificial CO_2_ gas cap immiscible stable gas flooding, an increase in the $N_{{{\text{GOI}}}}$ value is associated with a prolongation of the efficient stable development time, as well as an enhancement of the oil displacement and CO_2_ storage effect. For instance, in Experiments #4 and #5, where $N_{{{\text{GOI}}}}$ is less than 1, only a partial degree of stable gas flooding is achieved, and the overall displacement efficiency and CO_2_ storage volume are low. Conversely, in experiment #6, where $N_{{{\text{GOI}}}}$ is greater than 1, stable gas flooding is achieved, and the overall displacement efficiency and CO_2_ storage volume are high.

(6) Influence of liquid phase relative permeability at the gas displacing oil front on the effectiveness of oil displacement and CO_2_ storage

As demonstrated in Fig. 9f and Table 9, the liquid phase relative permeability at the gas displacing oil front exhibits a linear relationship with $N_{{{\text{GOI}}}}$. Moreover, as the liquid phase relative permeability increases, the $N_{{{\text{GOI}}}}$ value increases monotonically, and the gas displacing oil front is becoming more stable. It is evident that disparities in geological and developmental parameters among diverse reservoirs give rise to variations in $N_{{{\text{GOI}}}}$, despite the uniformity of the liquid phase relative permeability at the gas displacing oil front and its fluctuations. The $N_{{{\text{GOI}}}}$ values for the pilot region of the Yanling Oilfield and the fault block reservoirs in central and eastern China following water flooding development are all less than 1.0. This indicates that the gas displacing oil front is unstable and that gas breakthrough is occurring. However, as the liquid phase relative permeability at the gas displacing oil front increases, the front’s stability gradually improves. In the West Hackberry Oilfield, the $N_{{{\text{GOI}}}}$ values are all greater than 3.7, indicating a stable gas displacing oil front. As the liquid phase relative permeability at the gas displacing oil front increases, the stability of the gas displacing oil front improves further.

The liquid phase relative permeability at the gas displacing oil front is indicative the proportion of the liquid phase’s competitive flow resistance in the multiphase fluid flow in the porous medium at the gas displacing oil front. When the liquid phase relative permeability increases, the flow resistance of the liquid phase at the gas displacing oil front becomes smaller, and the fluidity of the liquid phase becomes better. Consequently, the utilisation of the artificial CO_2_ gas cap immiscible stable gas flooding is deemed appropriate for oil reservoirs characterised by a high liquid phase relative permeability at the gas displacing oil front. It is evident that when the mobility ratio and the gas phase relative permeability at the gas displacing oil front remains constant, and the liquid phase relative permeability at the gas displacing oil front is high, there is a stable gas flooding. This suggests that the possibility of gas breakthrough is low, and the effect of the gas displacing oil and CO_2_ sequestration are optimal.

In essence, comprehensive analysises are required to ascertain whether the higher liquid phase relative permeability at the gas displacing oil front is beneficial for oil displacement and CO_2_ storage in the process of gas flooding. These analysises must consider the physical mechanisms of gas flooding and the characteristics of the relative permeability curves. The high liquid phase relative permeability at the gas displacing oil front merely indicates that the liquid phase exhibits adequate fluidity at the saturation of the gas displacing oil front. However, the displacement efficiency is contingent on the competitive relationship between the gas–liquid two-phase flow capacity ( $\frac{{K_{{{\text{rog}}\angle }} }}{{K_{{{\text{rg}}\angle }} }}$ ). It is evident that the high fluidity of the gas phase at the gas displacing oil front will counteract the advantage of the oil phase (or liquid phase) permeability. Therefore, by engineering means to suppress gas breakthrough, the potential of the high liquid phase relative permeability at the gas displacing oil front can be realised. The key evaluation index is the liquid–gas mobility ratio at the gas displacing oil front ( $M_{{\text{o - g}}} = \frac{{K_{{{\text{rog}}\angle }} \mu_{{\text{g}}} }}{{K_{{{\text{rg}}\angle }} \mu_{{\text{o}}} }}$ ). When $M_{{\text{o - g}}} > 1$ (liquid mobility > gas mobility), the gas displacing oil front is stable, the sweep efficiency is high, which is conducive to the exploitation of the bypass oil and is beneficial to improving the oil recovery and the potential of CO_2_ sequestration. When $M_{{\text{o - g}}} < 1$ (gas mobility > liquid mobility), the injected gas is prone to form viscous fingering, resulting in gas breakthrough, reducing the sweep efficiency, and is not conducive to the exploitation of the bypass oil and is not beneficial to to improving the oil recovery and the potential of CO_2_ sequestration.

(7) Influence of air permeability in the stratigraphic direction on the effectiveness of oil displacement and CO_2_ storage

As demonstrated in Fig. 8c, e, and Table 7, it is evident that the strata dip, oil and gas density, oil and gas viscosity are the same, and the gas injection rates are comparable in Experiments #3 and #5. In comparison, the variation relationship between air permeability in the stratigraphic direction and $N_{{{\text{GOI}}}}$ is evident, despite the relative permeability of the liquid phase and gas phase having an effect. Furthermore, an increase in air permeability in the stratigraphic direction is associated with a rise in the $N_{{{\text{GOI}}}}$ value, thereby enhancing the stability of the gas displacing oil front. It has been demonstrated that an increase in air permeability in the stratigraphic direction results in a delay in the gas breakthrough time, an extension in the efficient and stable production time, and an enhancement in the final displacement efficiency, as well as an increase in the final CO_2_ sequestration volume. The experiment with a higher air permeability whose air permeability is 5.25 mD resulted in an injected gas volume of 0.318 PV before the gas breakthrough occurs, and the final displacement efficiency was 70.01%, with the final CO_2_ sequestration volume being 0.522 PV. On the contrary the experiment with a lower air permeability whose air permeability is 1.53 mD resulted in an injected gas volume of 0.237 PV before the gas breakthrough occurs, and the final displacement efficiency was 67.69%, with the final CO_2_ sequestration volume being 0.490 PV. The displacement efficiency and CO_2_ sequestration volume of the experiment with the higher air permeability were increased by 2.32% and 0.032 PV respectively compared to those of the experiment with the lower air permeability.

Figure 9g and Table 9 shows that the air permeability in the stratigraphic direction exhibits a linear relationship with $N_{{{\text{GOI}}}}$, and the $N_{{{\text{GOI}}}}$ value increases monotonically. Similarly, it is evident that disparities in geological and developmental parameters among diverse reservoirs give rise to variations in $N_{{{\text{GOI}}}}$, despite the uniformity of the air permeability in the stratigraphic direction and its fluctuations. The $N_{{{\text{GOI}}}}$ values for the West Hackberry Oilfield are all greater than 2.1, indicating a stable gas displacing oil front. It has been demonstrated that as the air permeability in the stratigraphic direction increases, the gas displacing oil front becomes even more stable. The $N_{{{\text{GOI}}}}$ value of fault block reservoirs in central and eastern China following water flooding development is less than 1.0 due to the significant influence of other geological and development parameters. This results in an unstable gas displacing oil front, a gas breakthrough, and as the air permeability in the stratigraphic direction increases, the stability of the gas displacing oil front gradually improves. When the air permeability in the stratigraphic direction for the Pilot region of the Yanling Oilfield exceeds approximately 3150 mD, the corresponding $N_{{{\text{GOI}}}}$ value exceeds 1.0. Furthermore, as the air permeability in the stratigraphic direction increases, the gas displacing oil front becomes increasingly stable. It has been demonstrated that when the air permeability in the stratigraphic direction is high, the gas breakthrough time is later, the stable displacement time is longer, and the recovery effect that has been achieved, as well as the effect of CO_2_ sequestration, are superior.

The increase in air permeability in the stratigraphic direction is beneficial for the artificial gas cap immiscible stable gas flooding. The greater the air permeability in the stratigraphic direction, the easier the injected gas can flow, which can quickly transmit pressure, maintain formation energy, delay the drop of reservoir pressure, and is conducive to improving the displacement efficiency. The gas sweep range is wider, which is beneficial for forming a stable gas displacing oil front. High permeability allows for a higher gas injection rate, reduces gas injection pressure, and reduces energy consumption. In reservoirs with strata dip, gas tends to flow upwards due to its low density and forms a secondary gas cap. In high-permeability oil layers, this phenomenon may be exacerbated, which is conducive to achieving stable gas flooding, exploiting the attic oil, and enhancing the potential for CO_2_ sequestration. However, in scenarios where the air permeability in the stratigraphic direction is excessively high and heterogeneity is pronounced (e.g., the presence of fractures or high-permeability bands), the gas may prematurely break through, thereby diminishing the displacement efficiency and the potential for CO_2_ sequestration.

(8) Influence of injected gas viscosity on the effectiveness of oil displacement and CO_2_ storage

Figure 9h and Table 9 shows that there is a linear relationship between the injected gas viscosity and $N_{{{\text{GOI}}}}$. Moreover, as the injected gas viscosity increases, the $N_{{{\text{GOI}}}}$ value decreases monotonically and the stability of the gas displacing oil front gradually strengthens. In the same way, it is evident that disparities in geological and developmental parameters among diverse reservoirs give rise to variations in $N_{{{\text{GOI}}}}$, despite the uniformity of the injected gas viscosity and its fluctuations. Due to the significant influence of other geological and development parameters of the oil reservoirs, the $N_{{{\text{GOI}}}}$ values in the Pilot region of the Yanling Oilfield and the fault block reservoirs in central and eastern China following water flooding development are all less than 1.0 Consequently the gas displacing oil front is unstable and the gas breakthrough occurs. However, as the injected gas viscosity increases, the stability of the gas displacing oil front gradually improves. In the West Hackberry Oilfield, the $N_{{{\text{GOI}}}}$ values are all greater than 4.4, and the gas displacing oil front is stable. It has been demonstrated that an increase in the injected gas viscosity the results in a corresponding enhancement of the stability of the gas displacing oil front.

It can thus be posited that the utilisation of an artificial gas cap immiscible stable gas flooding technique is appropriate for use with high-viscosity injected gases. In circumstances where the injected gas viscosity is elevated, and the viscosity difference between the injected gas and the crude oil is minimal, the inhibition of viscous fingering can be effective. This inhibition can stabilise the gas displacing oil front, minimise the likelihood of gas breakthrough, reduce the gas breakthrough time, and extend the stable gas flooding time. Furthermore, it can facilitate the exploitation of the attic oil and the bypass oil. Consequently, these factors can enhance the oil recovery and the potential of CO_2_ sequestration.

(9) Influence of gas phase relative permeability at the gas displacing oil front on the effectiveness of oil displacement and CO_2_ storage

As demonstrated in Fig. 9i and Table 9, the gas phase relative permeability at the gas displacing oil front exhibits a power function relationship with $N_{{{\text{GOI}}}}$. Moreover, as the gas phase relative permeability at the gas displacing oil front increases, the $N_{{{\text{GOI}}}}$ value increases monotonically. In addition, the gas displacing oil front is stable, but the stability gradually deteriorates. The $N_{{{\text{GOI}}}}$ values for the West Hackberry Oilfield, the pilot region of the Yanling Oilfield, and the fault block reservoirs in central and eastern China following water flooding development all fall within the steeply declining intervals of the power function curves. The $N_{{{\text{GOI}}}}$ values for the West Hackberry Oilfield are all greater than 4.3, indicating that the gas displacing oil front is stable. However, as the relative permeability at the gas displacing oil front increases, the stability of the gas displacing oil front gradually deteriorates. In the pilot region of theYanling Oilfield, it has been determined that when the gas phase relative permeability at the gas displacing oil front is approximately less than 0.0022, the corresponding $N_{{{\text{GOI}}}}$ value is greater than 1. This indicates that the gas displacing oil front is stable. As the gas phase relative permeability at the gas displacing oil front decreases, the stability of the gas displacing oil front increases. When the rgas phase relative permeability at the gas displacing oil front exceeds 0.0022, the corresponding $N_{{{\text{GOI}}}}$ value is less than 1.0, indicating that the gas displacing oil front is unstable and gas breakthrough occurs. It is evident that the $N_{{{\text{GOI}}}}$ value in the fault block reservoirs in central and eastern China following water flooding development is less than 1.0, due to the significant influence of other geological and development parameters. This instability of the gas displacing oil front, resulting in gas breakthrough, is indicative of an improvement in the stability of the gas displacing oil front as the gas phase relative permeability at the gas displacing oil front decreases.

The gas phase relative permeability at the gas displacing oil front is indicative the proportion of the gas phase’s competitive flow resistance in the multiphase fluid flow in the porous medium at the gas displacing oil front. An increase in the gas phase relative permeability at the gas displacing oil front(i.e., at the gas–oil interface) results in a reduction in the flow resistance at the gas–oil interface, Concurrently, the fluidity of the gas phase is increased. It can thus be concluded that the artificial gas cap immiscible stable gas flooding is particularly effective in oil reservoirs characterised by a low gas phase relative permeability at the gas displacing oil front. In the absence of alterations in both the relative permeability of the gas phase relative permeability at the gas displacing oil front and the mobility ratio, when the gas phase relative permeability at the gas displacing oil front is lower, the probability of gas breakthrough is reduced, thus facilitating a more stable gas flooding. Consequently, the recovery effect that has been achieved, and the capacity for CO_2_ sequestration, are superior.

In essence, comprehensive analysises are required to ascertain whether the lower gas phase relative permeability at the gas displacing oil front is beneficial for oil displacement and CO_2_ storage in the process of gas flooding. These analysises must consider the physical mechanisms of gas flooding and the characteristics of the relative permeability curves. The low gas phase relative permeability at the gas displacing oil front merely indicates that the gas phase exhibits poor fluidity at the saturation of the gas displacing oil front. However, the displacement efficiency is contingent on the competitive relationship between the gas–liquid two-phase flow capacity ( $\frac{{K_{{{\text{rog}}\angle }} }}{{K_{{{\text{rg}}\angle }} }}$ ). It is evident that the low fluidity of the gas phase at the gas displacing oil front will enhance the advantage of the oil phase (or liquid phase) permeability, facilitating the release of the potential of the iquid phase relative permeability at the gas displacing oil front. The key evaluation index is the liquid–gas mobility ratio at the gas displacing oil front ( $M_{{\text{o - g}}} = \frac{{K_{{{\text{rog}}\angle }} \mu_{{\text{g}}} }}{{K_{{{\text{rg}}\angle }} \mu_{{\text{o}}} }}$ ). When $M_{{\text{o - g}}} > 1$ (liquid mobility > gas mobility), the gas displacing oil front is stable, the sweep efficiency is high, which is conducive to the exploitation of the bypass oil and is beneficial to improving the oil recovery and the potential of CO_2_ sequestration. When $M_{{\text{o - g}}} < 1$ (gas mobility > liquid mobility), the injected gas is prone to form viscous fingering, resulting in gas breakthrough, reducing the sweep efficiency, and is not conducive to the exploitation of the bypass oil and is not beneficial to to improving the oil recovery and the potential of CO_2_ sequestration.

In summary, although the oil reservoirs differ and their geological and development parameters vary, the functional relationship between the same influencing factors and $N_{{{\text{GOI}}}}$ is clear and the patterns of influence are consistent (regardless of whether the gas displacing oil fronts in the oil reservoirs are stable). The distinction between the laws of the influence of the same factors on $N_{{{\text{GOI}}}}$ is determined by two factors. Firstly, the gradient of the linear relationship, and secondly, the exponent of the power function relationship. Specifically, when the same influencing factors and their changes are observed, the corresponding $N_{{{\text{GOI}}}}$ values and their change magnitudes are found to differ. When utilising the average geological and developmental data in the West Hackberry Oilfield as the fundamental parameters, it is evident that the $N_{{{\text{GOI}}}}$ values within the range of changes for each influencing factor are all greater than 1.0. This finding indicates that, by employing the average geological and developmental parameters as the baseline, the reservoir can achieve a flue gas cap immiscible stable gas flooding process. When the reservoir parameters are based on the average values in the pilot area of the Yanling Oilfield, only under conditions where the crude oil viscosity is approximately less than 8.5 mPa s, the gas injection rate is approximately less than 0.0608 m/d, the air permeability in the stratigraphic direction is approximately greater than 3150 mD, and the gas phase relative permeability at the gas displacing oil front is approximately less than 0.0022, will the corresponding $N_{{{\text{GOI}}}}$ values be greater than 1.0, and this results the N_2_ gas cap immiscible stable flooding process. Similarly, the present study employs the average geological and developmental data in the fault block reservoirs in central and eastern China following water flooding development as their basic parameters. The resultant reservoir exhibits CO_2_ gas cap immiscible stable gas flooding process when only the crude oil viscosity is approximately less than 2.1 mPa s, with the corresponding $N_{{{\text{GOI}}}}$ value greater than 1.0. Therefore, it is both theoretically and practically feasible to conduct a sensitivity analysis of the influencing factors affecting the artificial gas cap immiscible stable gas flooding process, and to analysis the mechanical mechanism during this process, using the average geological and development parameters of the West Hackberry Oilfield as the base parameters.

#### Sensitivity analysis of influencing factors

Given that the functional relationship between the influencing factors affecting the artificial gas cap immiscible stable gas flooding process and $N_{{{\text{GOI}}}}$ are all monotonic models, the sensitivity coefficient^45–47^ is employed to represent the sensitivity of each influencing factor in this paper, which represents the ratio of the percentage change in project analysis indicators to the percentage change in uncertain factors and is derived from the Engineering Economics. The computational formula for the sensitivity coefficient ($S_{{{\text{AF}}}}$) is provided in Eq. (33) as follows.

33 $$S_{{{\text{AF}}}} = \frac{{\frac{\Delta A}{A}}}{{\frac{\Delta F}{F}}}$$

As demonstrated in Eq. (33), $S_{{{\text{AF}}}}$ is the ratio of the percentage change in the project analysis indicator to the percentage change in the uncertain factor, reflecting the sensitivity of evaluation indicator $A$ to the uncertain influencing factor $F$; $\frac{\Delta F}{F}$ is the rate of change of influencing factor $F$; When the influencing factor $F$ changes $\Delta F$, $\frac{\Delta A}{A}$ represents the corresponding change rate of the evaluation indicator $A$. In the event of a change in the artificial gas cap immiscible stable gas flooding number ($N_{{{\text{GOI}}}}$) in the same direction as the influencing factors, the sensitivity coefficient ($S_{{{\text{AF}}}}$) will be greater than 0 (i.e. $S_{{{\text{AF}}}} > 0$). Conversely, the sensitivity coefficient ($S_{{{\text{AF}}}}$ ) will be less than 0 (i.e. $S_{{{\text{AF}}}} < 0$). The greater the absolute value of the sensitivity coefficient ($S_{{{\text{AF}}}}$), the stronger the sensitivity of the influencing factors. Otherwise, the sensitivity coefficient will be negligible.

**Fig. 10:**
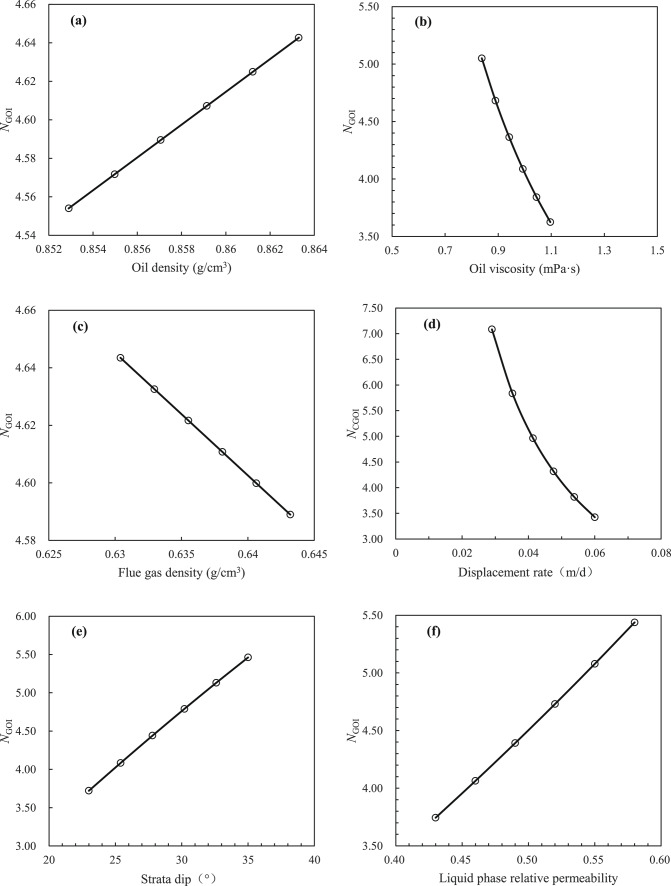

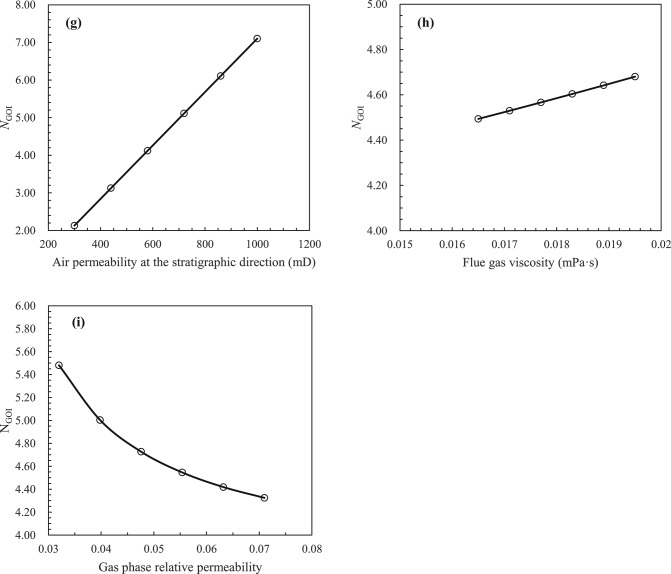
Relationship curves of $N_{{{\text{GOI}}}}$ versus the oil density (**a**), the oil viscosity (**b**), the flue gas density (**c**), the displacement rate i.e. the gas injection rate, or the gas cutting rate (**d**), the strata dip (**e**), the liquid phase relative permeability (**f**), the air permeability at the stratigraphic direction (**g**), the flue gas viscosity (**h**), the gas phase relative permeability (**i**) in the West Hackberry Oilfield.

As demonstrated in Fig. 10 and Table 10, the factors affecting the assessment model ($N_{{{\text{GOI}}}}$) and the development effect of the artificial gas cap immiscible stable gas flooding process, in descending order of importance, are as follows: crude oil density, liquid phase relative permeability at the gas displacing oil front, air permeability in the stratigraphic direction, crude oil viscosity, strata dip, gas cap gas density (injected gas density), gas injection rate (gas–cut rate), gas cap gas viscosity (injected gas viscosity), and gas phase relative permeability at the gas displacing oil front. Among these, the crude oil properties (i.e. its density, liquid phase relative permeability, and viscosity) are the key influencing factors, and their cumulative contribution to the development effect of the artificial gas cap immiscible stable gas flooding is as high as 53.13%. Secondary factors, including the reservoir and rock properties (e.g. air permeability, strata dip), injected gas properties (e.g. gas density, gas viscosity, gas phase relative permeability), and gas injection rate, contribute cumulatively to the development effect of the artificial gas cap immiscible stable gas flooding, with respective contributions of 26.46%, 13.67%, and 6.74%.

**Table 10:** Calculation results of the sensitivity coefficient during the process of artificial gas cap stable flooding in the West Hackberry field.

Influencing factors	Rate of change		$S_{{{\text{AF}}}}$	Sort by value size	Rate of contribution
	${{\Delta F} \mathord{\left/ {\vphantom {{\Delta F} F}} \right. \kern-0pt} F}$	${{\Delta A} \mathord{\left/ {\vphantom {{\Delta A} A}} \right. \kern-0pt} A}$			
Oil density	0.0122	0.0195	1.5957	1	22.26%
Oil viscosity	0.3080	− 0.2822	− 0.9161	4	12.78%
Flue gas density	0.0203	− 0.0117	− 0.5783	6	8.07%
Flue gas viscosity	0.1818	0.04515	0.2283	8	3.18%
Displacement rate	1.0670	− 0.5167	− 0.4833	7	6.74%
Strata dip	0.5217	0.4680	0.8969	5	12.51%
Air permeability at the stratigraphic direction	2.3333	2.3333	1	3	13.95%
Liquid phase relative permeability	0.3488	0.4526	1.2975	2	18.10%
Gas phase relative permeability	1.2188	− 0.2111	− 0.1732	9	2.42%

### Mechanical mechanisms of the artificial gas cap stable flooding

Actually, the Eq. (31) can be transformed into the following form:

34 $$N_{{{\text{GOI}}}} = \frac{{\rho _{{\text{o}}} g{\text{sin}}\alpha + \left( {\rho _{{\text{o}}} - \rho _{{\text{g}}} } \right)g{\text{sin}}\alpha }}{{\frac{{\mu _{{\text{o}}} v_{{\text{t}}} }}{{k_{\angle } K_{{{\text{rog}}\angle }} }} - \frac{{\mu _{{\text{g}}} v_{{\text{t}}} }}{{k_{\angle } K_{{{\text{rg}}\angle }} }}}}$$

As demonstrated in Eq. (34), the values of $\rho _{{\text{o}}} g{\text{sin}}\alpha$, $\left( {\rho_{{\text{o}}} - \rho_{{\text{g}}} } \right)g{\text{sin}}\alpha$, $\frac{{\mu_{{\text{o}}} v_{{\text{t}}} }}{{k_{\angle } K_{{{\text{rog}}\angle }} }}$, and $\frac{{\mu_{{\text{g}}} v_{{\text{t}}} }}{{k_{\angle } K_{{{\text{rg}}\angle }} }}$ represent the gravity gradient per unit volume of crude oil at stratigraphic direction, the gradient of difference between buoyancy and gravity per unit volume of the gas at stratigraphic direction, the driving pressure gradient per unit volume of crude oil at stratigraphic direction, and the driving pressure gradient per unit volume of the gas at stratigraphic direction, respectively. In summary, the value of ${N}_{\text{GOI}}$ is contingent on the gravity gradient, the buoyancy gradient, and the driving pressure gradient. The differing behaviors of the gas oil interface in the process of the artificial gas cap flooding are contingent upon the equilibrium between the aforementioned gradients. In this system, the gravity gradient functions as the driving force for gas displacing oil. The magnitude of this gradient is contingent upon the disparity in the density difference between oil and gas. The buoyancy gradient exerts a resistance to gas invasion, with its magnitude determined by the crude oil density. The driving pressure gradient serves as the driving force for gas displacing oil. The magnitude of this gradient is dependent on the size of the expansion energy of the artificial gas cap and the liquid phase (i.e. crude oil–gas mixture phases).

**Fig. 11:**
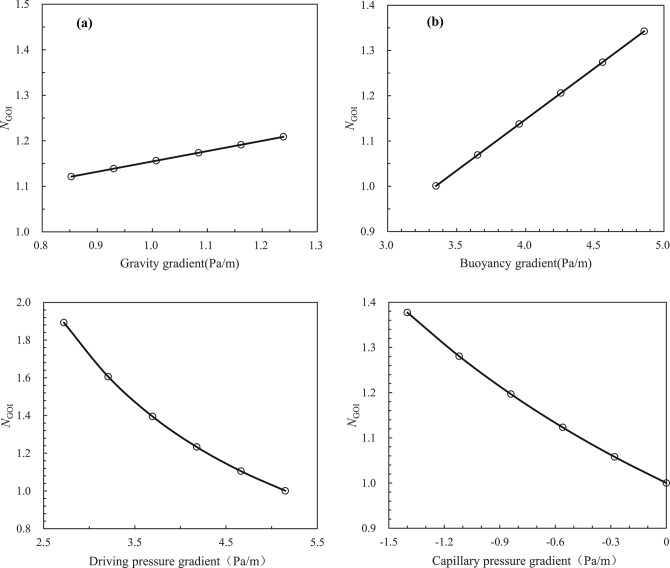
Relationship curves of $N_{{{\text{GOI}}}}$ versus gravity gradient (**a**), Buoyancy gradient (**b**), driving pressure gradient (**c**), capillary pressure gradient (**d**) .

As demonstrated in Fig. 11, Tables 11 and 12, the influence of the buoyancy gradient, the driving pressure gradient, and the gravity gradient on the assessment model (i.e. the artificial gas cap immiscible stable gas flooding number) ($N_{{{\text{GOI}}}}$) or development effect of the artificial gas cap stable flooding process decreases successively. Among these acting forces, the buoyancy and the driving pressure are of particular significance, contributing 51.99% and 36.20% respectively to the development effect during the artificial gas cap immiscible stable gas flooding process. It is evident that the gravity is a secondary factor, contributing 11.81% to the development effect during the artificial gas cap immiscible stable gas flooding process. A linear relationship exists between the gravity gradient, the buoyancy gradient and $N_{{{\text{GOI}}}}$, whereby $N_{{{\text{GOI}}}}$ monotonically increases as the gas displacing oil front (i.e. gas oil interface) becomes increasingly stable as the artificial gas cap immiscible stable gas flooding progresses, with the increase of the gravity gradient and the buoyancy gradient. Meanwhile, a power function relationship exists between the driving pressure gradient and $N_{{{\text{GOI}}}}$, whereby the driving pressure gradient increases, the gas displacing oil front (i.e. gas oil interface) of the artificial gas cap immiscible stable gas flooding process becomes increasingly unstable. It has been demonstrated that when the driving pressure gradient increases to approximately 5.5136 Pa.m, $N_{{{\text{GOI}}}}$ decreases to around 1.0 and then begins to decrease slowly. The onset of gas breakthrough is concomitant with this decline in $N_{{{\text{GOI}}}}$, and the development effectiveness deteriorates accordingly.

**Table 11:** Functional relationship model of $N_{{{\text{GOI}}}}$ versus the gravity gradient, tthe buoyancy gradient, the driving pressure gradient, the capillary pressure gradient.

Influencing factors	Functional relationship model
Gravity gradient	$$N_{{{\text{GOI}}}} = 0.2268\left( {\rho_{{\text{o}}} - \rho_{{\text{g}}} } \right)g\sin \alpha \begin{array}{*{20}c} { + 0.9278} & {} \\ \end{array} R^{2} { = }1$$
Buoyancy gradient	$$N_{{{\text{GOI}}}} = 0.2268\rho_{{\text{o}}} g\sin \alpha \begin{array}{*{20}c} { + 0.241} & {} \\ \end{array} R^{2} { = }1$$
Driving pressure gradient	$$N_{{{\text{GOI}}}} = 5.1536 \times \left( {\frac{{\mu_{{\text{o}}} v_{{\text{t}}} }}{{k_{\angle } K_{{{\text{rog}}\angle }} }} - \frac{{\mu_{{\text{g}}} v_{{\text{t}}} }}{{k_{\angle } K_{{{\text{rg}}\angle }} }}} \right)^{ - 1} \begin{array}{*{20}c} {} & {} \\ \end{array} R^{2} { = }1$$
Capillary pressure gradient	$$N_{{{\text{GOI}}}} = 0.9935e^{{ - 0.228 \times \left( {\frac{{\mu_{{\text{o}}} v_{{\text{t}}} }}{{k_{\angle } K_{{{\text{rog}}\angle }} }} - \frac{{\mu_{{\text{g}}} v_{{\text{t}}} }}{{k_{\angle } K_{{{\text{rg}}\angle }} }} - \left( {2\rho_{{\text{o}}} - \rho_{{\text{g}}} } \right)g\sin \alpha } \right)}} \begin{array}{*{20}c} {} & {} \\ \end{array} R^{2} { = }0.9978$$

**Table 12:** Contribution of the gravity gradient, the buoyancy gradient, and the driving pressure gradient during the process of artificial gas cap stable flooding in the West Hackberry Oilfield.

Force	Rate of change		$S_{{{\text{AF}}}}$	Sort by value size	Rate of contribution
	$\frac{\Delta F}{F}$	$\frac{\Delta A}{A}$			
Gravity gradient	0.4521	0.0780	0.1725	3	11.81%
Buoyancy gradient	0.4500	0.3417	0.7592	1	51.99%
Driving pressure gradient	0.8914	− 0.4713	− 0.5287	2	36.20%

Furthermore, Eq. (19) can also be transformed as follows:

35 $$\frac{{\mu_{{\text{o}}} v_{{\text{t}}} }}{{k_{\angle } K_{{{\text{rog}}\angle }} }} - \frac{{\mu_{{\text{g}}} v_{{\text{t}}} }}{{k_{\angle } K_{{{\text{rg}}\angle }} }} = \frac{{\partial p_{{{\text{c}}\left( {\text{g - o}} \right)}} }}{\partial x} + \rho_{{\text{o}}} g\sin \alpha + \left( {\rho_{{\text{o}}} - \rho_{{\text{g}}} } \right)g\sin \alpha$$

The combination of Eqs. (34) and (35) yields the following result:

36 $$N_{{{\text{GOI}}}} = \frac{1}{{1 + \frac{{\frac{{\partial p_{{{\text{c}}\left( {\text{g - o}} \right)}} }}{\partial x}}}{{\rho_{{\text{o}}} g{\text{sin}}\alpha + \left( {\rho_{{\text{o}}} - \rho_{{\text{g}}} } \right)g{\text{sin}}\alpha }}}}$$

It can thus be concluded that the left-hand side of Eq. (35) represents the difference between the driving pressure gradient of the liquid phase (i.e. oil–gas mixture phases) per unit volume of crude oil and the driving pressure gradient per unit volume of the gas at stratigraphic direction. The left-hand side of Eq. (35) is comprised of the gas–crude oil capillary force gradient, the gravity gradient per unit volume of crude oil at stratigraphic direction, and the gradient of the difference between buoyancy and gravity per unit volume of the gas at stratigraphic direction. It can thus be concluded that the driving pressure gradient of the artificial gas cap stable flooding reservoir is determined by the capillary force gradient, the gravity gradient and the buoyancy gradient. Furthermore, from Eqs. (34) and (36), it can be observed that ${N}_{\text{GOI}}$ is determined by the capillary force gradient, the gravity gradient and the buoyancy gradient. Therefore, the morphology of the gas oil interface in the process of the artificial gas cap flooding is contingent upon the equilibrium between the capillary force gradient, the gravity gradient and the buoyancy gradient. Among them, the gravity gradient is identified as the primary driving force for gas–driven oil, while the capillary pressure gradient and the buoyancy gradient are recognised as the resistance forces for gas intrusion.

As demonstrated in Fig. 11, Tables 11 and 13, the impact of acting forces such as buoyancy, capillary and gravity on the assessment model ($N_{{{\text{GOI}}}}$) or the development effect of the artificial gas cap immiscible stable gas flooding process diminishes in succession. It is imperative to acknowledge the pivotal role of the buoyancy, which has been demonstrated to contribute significantly to the development effect of the artificial gas cap immiscible stable gas flooding process, accounting for up to 62.98% of the observed effectiveness. Evidently, the capillary force and the gravity are secondary factors, contributing 22.71% and 14.31% respectively to the development effectiveness of the artificial gas cap immiscible stable gas flooding process. Furthermore, a linear relationship is observed between the gravity gradient, the buoyancy gradient and ${N}_{\text{GOI}}$. With an increase in the gravity gradient and the buoyancy gradient, ${N}_{\text{GOI}}$ exhibits a monotonically increase. Concurrently, an exponential function or an approximate linear relationship is observed between the capillary gradient and $N_{{{\text{GOI}}}}$. As the capillary gradient increases, $N_{{{\text{GOI}}}}$ decreases monotonically until it reaches 1.0, at which point the capillary gradient is 0, and the immiscible gas flooding process transitions to the miscible gas flooding process.

**Table 13:** Contribution of the gravity gradient, the buoyancy gradient and the capillary gradient during the process of arcitifical gas cap stable flooding in the West Hackberry Oilfield.

Force	Rate of change		$S_{{{\text{AF}}}}$	Sort by value size	Rate of contribution
	$\frac{\Delta F}{F}$	$\frac{\Delta A}{A}$			
Gravity gradient	0.4521	0.0780	0.1725	3	14.31%
Buoyancy gradient	0.4500	0.3417	0.7592	1	62.98%
Capillary force gradient	− 1.0000	− 0.2738	0.2738	2	22.71%

From Eq. (34), we can obtain the following:

37 $$\frac{\partial p}{{\partial x}} = \frac{{\mu_{{\text{o}}} v_{{\text{t}}} }}{{k_{\angle } K_{{{\text{rog}}\angle }} }} - \frac{{\mu_{{\text{g}}} v_{{\text{t}}} }}{{k_{\angle } K_{{{\text{rg}}\angle }} }}$$

As can be observed from Eq. (37), the driving pressure gradient represents the difference between the driving pressure gradient of the liquid phase (i.e. the oil–gas mixture phases) and the driving pressure gradient of the gas phase. Its magnitude is determined by the viscous force and the additional resistance, which is reflected in the value of the phase permeability of each phase.

**Fig. 12:**
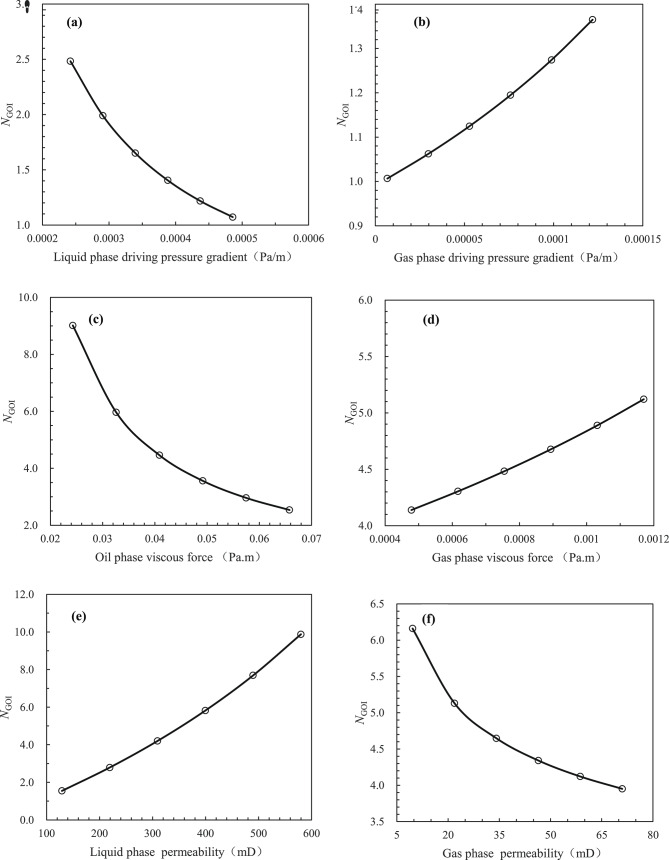
Relationship curves of $N_{{{\text{GOI}}}}$ versus the liquid phase driving pressure gradient (**a**), the gas phase driving pressure gradient (**b**), the oil phase viscous force (**c**), the gas phase viscous force (**d**), the liquid phase permeability (**e**), the gas phase permeability (**f**).

As illustrated in Figs. 11, 12, Tables 14 and 15, the influence of forces such as the buoyancy, the liquid phase driving pressure, the gravity and the gas phase driving pressure on the assessment model ($N_{{{\text{GOI}}}}$) or the development effect of the artificial gas cap immiscible stable gas flooding process decreases successively. Among these acting forces, the buoyancy and the liquid phase driving pressure were found to be the key contributors to the development effectiveness of the artificial gas cap immiscible stable gas flooding process, accounting for 50.07% and 37.18%, respectively. The gravity of the subject had been identified as a secondary factor, contributing 11.38% to the development effectiveness of the artificial gas cap immiscible stable gas flooding process. The gas phase driving pressure was found to be a minor factor, contributing only 1.37% to the development effectiveness of the artificial gas cap immiscible stable gas flooding process. Moreover, the correlation between the gravity gradient, the buoyancy gradient and $N_{{{\text{GOI}}}}$ is linear, whereby $N_{{{\text{GOI}}}}$ increases in tandem with the gravity gradient and the buoyancy gradient. The liquid phase driving pressure gradient had been shown to exhibit a power-law relationship with $N_{{{\text{GOI}}}}$, and $N_{{{\text{GOI}}}}$ has been observed to decrease sharply with the increase in the liquid phase driving pressure gradient, leading to a gradual weakening of the stability of the gas displacing oil front. When the liquid phase (oil phase) driving pressure gradient increases to 0.00048 Pa m, $N_{{{\text{GOI}}}}$ decreases to around 1.0, subsequently it begins to decrease slowly, and gas breakthrough begins to occur. The gas phase driving pressure gradient exhibits an exponential function (approximately linear) relationship with $N_{{{\text{GOI}}}}$, and as the gas phase driving pressure gradient increases, $N_{{{\text{GOI}}}}$ increase sharply, meanwhile the gas displacing oil front becomes increasingly stable during the artificial gas cap immiscible stable gas flooding process.

**Table 14:** Functional relationship model of $N_{{{\text{GOI}}}}$ versus the liquid phase driving pressure gradient, the gas phase driving pressure gradient, the oil phase viscous force gradient, the gas phase viscous force gradient, the liquid phase permeability, the gas phase permeability.

Influencing factors	Functional relationship model
Liquid phase driving pressure gradient	$$N_{{{\text{GOI}}}} = 0.0001 \times \left( {\frac{{\mu_{{\text{o}}} v_{{\text{t}}} }}{{k_{\angle } K_{{{\text{rog}}\angle }} }}} \right)^{ - 1.205} \begin{array}{*{20}c} {} & {} \\ \end{array} R^{2} { = }1$$
Gas phase driving pressure gradient	$$N_{{{\text{GOI}}}} = 0.9829e^{{2641.2\frac{{\mu_{{\text{g}}} v_{{\text{t}}} }}{{k_{\angle } K_{{{\text{rg}}\angle }} }}}} \begin{array}{*{20}c} {} & {} \\ \end{array} R^{2} { = }1$$
Oil phase viscous force	$$N_{{{\text{GOI}}}} = 0.0078 \times \left( {\mu_{{\text{o}}} v_{{\text{t}}} } \right)^{ - 1.27} \begin{array}{*{20}c} {} & {} \\ \end{array} R^{2} { = }0.9985$$
Liquid phase permeability	$$N_{{{\text{GOI}}}} = 3.5615e^{{307.75\mu_{{\text{g}}} v_{{\text{t}}} }} \begin{array}{*{20}c} {} & {} \\ \end{array} R^{2} { = }0.999$$
Gas phase permeability	$$N_{{{\text{GOI}}}} = 0.0038 \times \left( {k_{\angle } K_{{{\text{rog}}\angle }} } \right)^{1.2287} \begin{array}{*{20}c} {} & {} \\ \end{array} R^{2} { = }0.9974$$
Gas phase permeability	$$N_{{{\text{GOI}}}} = 10.178 \times \left( {k_{\angle } K_{{{\text{rg}}\angle }} } \right)^{ - 0.222} \begin{array}{*{20}c} {} & {} \\ \end{array} R^{2} { = }0.9575$$

**Table 15:** Contribution of the gravity gradient, the buoyancy gradient, liquid phase driving force gradient, and the gas phase driving force gradient during the process of arcitifical gas cap stable flooding in the West Hackberry field.

Force	Rate of change		$S_{{{\text{AF}}}}$	Sort by value size	Rate of contribution
	$\frac{\Delta F}{F}$	$\frac{\Delta A}{A}$			
Gravity gradient	0.4521	0.0780	0.1725	3	11.38%
Buoyancy gradient	0.4500	0.3417	0.7592	1	50.07%
Liquid phase driving force gradient	1.008	− 0.5683	− 0.5638	2	37.18%
Gas phase driving force gradient	17.0839	0.3560	0.0208	4	1.37%

As demonstrated in Figs. 11, 12, Tables 14 and 16, the impact of various factors, including the liquid phase permeability, the buoyancy, the oil viscous force, the gravity, the oil gas viscous force, and the gas phase permeability on the assessment model ($N_{{{\text{GOI}}}}$) or the oil reservoir development effect of the artificial gas cap immiscible stable gas flooding process exhibits a decrease in order of importance. It is noteworthy that the liquid phase permeability and the gas phase permeability reflect the additional resistance of the liquid and gas phases, respectively, in multiphase flow, which characterises the strength of fluid seepage; the viscosity is the resistance to oil and gas phase migration, and characterises the ease or difficulty of fluid flow. However, during the artificial gas cap immiscible stable gas flooding process, the adhesion force exerts influence on the viscous force, the capillary force, the additional resistance, and other factors. The coupling mechanism is more intricate, and thus, further elaboration is unnecessary here. The liquid phase permeability (i.e. the liquid phase additional resistance) is a pivotal factor, contributing up to 48.76% to the development effectiveness of the artificial gas cap immiscible stable gas flooding process. This reflects the significant influence of the liquid phase flow capacity on artificial gas cap immiscible stable gas flooding process. The buoyancy, the oil phase viscous force, the gravity, and the gas phase viscous force are secondary factors, contributing 23%, 13.29%, 5.44%, and 5.18%, respectively, to the development effectiveness of the artificial gas cap immiscible stable gas flooding process. The gas phase permeability (i.e. the gas phase additional resistance) was found to be a minor factor, with a contribution of only 3.35% to the development effectiveness of the artificial gas cap immiscible stable gas flooding process. The research findings indicate that there is a power function relationship between the liquid phase permeability (i.e. the liquid phase additional resistance), the gas phase permeability (i.e. the gas phase additional resistance), the oil viscous force and $N_{{{\text{GOI}}}}$. Among these acting forces, an increase in the liquid phase permeability (i.e. the liquid phase additional resistance) results in a sharp rise in $N_{{{\text{GOI}}}}$. Concurrently, the gas displacing oil front exhibits enhanced stability during the artificial gas cap immiscible stable gas flooding process. Conversely, the value of decrease slowly in $N_{{{\text{GOI}}}}$ was observed to be in accordance with an increase in the he gas phase permeability (i.e. the gas phase additional resistance). The gas displacing oil front of the artificial gas cap immiscible stable gas flooding process becomes increasingly unstable. The decrease in $N_{{{\text{GOI}}}}$ is gradual, coinciding with an increase in the oil phase viscous force. Concurrently, the stability of the artificial gas cap immiscible stable gas flooding process undergoes a gradual deterioration. The gas phase viscous force and $N_{{{\text{GOI}}}}$ is related by an exponential function (or approximate straight line), and $N_{{{\text{GOI}}}}$ increases sharply as the gas phase viscous force increases. The stability of the artificial gas cap immiscible stable gas flooding process improves gradually.

**Table 16:** Contribution of the oil viscous force, the gas viscous force, the liquid phase permeability, the gas phase permeability during the process of arcitifical gas cap stable flooding in the West Hackberry field.

Force	Rate of change		$S_{{{\text{AF}}}}$	Sort by value size	Rate of contribution	Descr
	${{\Delta F} \mathord{\left/ {\vphantom {{\Delta F} F}} \right. \kern-0pt} F}$	${{\Delta A} \mathord{\left/ {\vphantom {{\Delta A} A}} \right. \kern-0pt} A}$				
Gravity gradient	0.4521	0.0780	0.1725	4	5.44%	
Buoyancy gradient	0.4500	0.3417	0.7592	2	23.96%	
Oil viscous force	1.7063	− 0.7187	− 0.4212	3	13.29%	
Gas viscous force	1.4451	0.2373	0.1642	5	5.18%	
Liquid phase permeability	3.4961	5.4013	1.5449	1	48.76%	Liquid phase additional resistance
Gas phase permeability	2.2450	− 0.2383	− 0.1061	6	3.35%	Gas phase additional resistance

### Feasibility of the artificial gas cap immiscible stable gas flooding

From Fig. 3, it can be seen that the artificial gas cap stable flooding proposed in this paper is different from other forms of continuous gas injection flooding that was proposed and perfected by Rao, et al.^48^ , Pang^49^, Liang, et al.^50^, Guo, et al.^25^, Saikia and Rao^51^, Zhou, et al.^52^, and Ren, et al.^9^, and artificial N_2_ gas cap flooding that was proposed and perfected by Diwu, et al.^53^, Chang, et al.^54^, Liu, et al.^55^, Xu^56^, Zhao^57^, and Liu^58^, which have the following characteristics: (1) The gas cap in an oil reservoir with certain strata dip, has a large volume. Gas is artificially injected into the gas cap (or strata), and the cumulative injection volume is large enough to maintain a constant formation pressure throughout the oil and gas production process. This scenario shows that the secondary artificial CO_2_ gas cap has a significant volume and is capable of providing significant energy enhancement. It also shows that the large-scale sequestration of CO_2_ in the form of gas caps can be achieved before or during the immiscible stable gas flooding. (2) As a consequence of sufficient formation pressure, the reservoir transient production begins to remain constant and then increases sharply as the GOI moves down and gas cutting occurs. (3) As the formation pressure exceeds the saturation pressure, the production gas–oil ratio initially remains constant but then increases after gas cutting. (4) Due to the abundant supply of production energy in the oil reservoir, the volume of CO_2_ gas invasion or CO_2_ gas injection completely compensates the volume of liquid production, the sweep efficiency is equal to 1.0, and the injection-production ratio is equal to 1.0, indicating that the CO_2_ gas to oil replacement ratio is 1:1.

It has been demonstrated that the development practice of a large gas cap reservoir will only exhibit the above exploitation characteristics or some of them when the gas cap index is greater than 1.5 or even higher than 4.8^58–63^. The reason may be that these reservoirs, which have a small oil ring and a large gas cap, require both oil and gas production. In the absence of natural gas production and the presence of only oil production, analogous characteristics of artificial gas cap stable flooding may manifest^61–63^. Consequently, the artificial gas cap stable flooding in the fault block oil reservoirs with specific stratigraphic dips is a potential outcome.

In addition, 0.707825 × 10^8^ m^3^ of air has been injected into the West Hackberry tertiary project over the past 6 years. The ultimate recovery of the OOIP is approaching 90% while its waterflood recoveriesis 50%-60%^2,3,33,64^. It is evident that the feasibility of the theory and technology of artificial stable flooding, together with its remarkable progress, has been fully validated. The authors intend to prioritise research on the artificial gas cap stable flooding theory and technology in their subsequent studies.

### CO_2_ storage potential during the artificial gas cap immiscible stable gas flooding

From the above analysis, it can be concluded that in the process of the artificial gas cap flooding, if no gas cutting occurs or if the gas drive is always immiscible and stable, the geological CO_2_ storage efficiency can reach 100%. This means the realisation of industrial large-scale CO_2_ structural storage. in addition, the ratio of underground gas to oil volume formed in the process of the artificial gas cap flooding is greater than 1.5, or even up to 4.8 or more. At this time, the rigid CO_2_ gas cap can be sequestered in the CO_2_ volume as high as 4.8 times the volume of crude oil in the form of structural storage, and the potential for CO_2_ storage is significant. Moreover, the viscosity of the CO_2_ gas–oil system is lower, and the gas solubility, EOR potential, dissolution and storage potential of CO_2_ are all significantly higher than those of N_2_ and other hydrocarbon gases. Therefore, the development technology of the artificial gas cap flooding has a perfect potential and application prospect for greatly improving oil recovery and large-scale CO_2_ geological storage in the fault block reservoir with certain strata dip. The theoretical and practical implications of the study are also significant.

### Suitability of the artificial gas cap immiscible stable gas flooding

Many reservoir suitability screening criteria have been proposed in the literature for the gas cap flooding process, e.g. Wang^65^, Rivas, et al.^66^, Diaz, et al.^67^, Lepski, et al.^68^, Pang, et al.^69^. It can be observed that the most of these criteria have a significant influence on the development effect, as evidenced by empirical evidence from studies employing gas cap flooding. These criteria have been shown to be effective in guiding decision-making processes, and the aforementioned criteria encompass a comprehensive array of factors, including structural form, reservoir physical properties, crude oil properties, injection production technology, and related parameters. The primary influence parameters primarily comprise the strata dip, the fluid density, the reservoir pressure, the air permeability, the variation coefficient, the heterogeneity, encompassing the sedimentary rhythm, the degree of interlayer development, and the high-angle fracture, the reservoir thickness or the bandwidth, the crude oil viscosity, the wettability index, the interfacial tension, the oil saturation, the cumulative gas injection volume, the gas injection rate, and so forth. Typically, these parameters are initially selected as screening indicators for evaluation criteria, and subsequently, the key factors are selected using a grey-related method, response-surface method, orthogonal test method, range analysis and analog analysis method. The optimal variation ranges of these key factors are then determined, and the screening and evaluation criteria of the gas cap flooding process in the oil reservoir are set. The analyses concluded that the aforementioned criteria and their indicators lack comprehensiveness, are too numerous and complex, and are difficult to obtain relevant parameters for. As a result, it is not possible to quickly and accurately screen and evaluate suitable oil reservoirs for the gas cap flooding process in practical applications. Furthermore, it is challenging to measure the influences of the parameters and determine the reasonable ranges of the parameters. Consequently, the universalities of the existing screening and evaluation criteria are limited.

In contrast, the factors influencing the artificial gas cap number ($N_{{{\text{GOI}}}}$) are clear, primary and secondary distinct, easily obtainable. In order to calculate $N_{{{\text{GOI}}}}$, it is necessary to input the following parameters into the model formula: the density of the crude oil, the viscosity of the crude oil, the density of the gas, the rate of the gas injection (i.e. the rate of the gas cutting), the strata dip, the relative permeability of the liquid phase, the permeability of the air in the stratigraphic direction, the viscosity of the gas, the relative permeability of gas phase and other parameters.

It can be concluded that an oil reservoir is suitable for the artificial gas cap flooding development if the calculated value of the artificial gas cap flooding number ($N_{{{\text{GOI}}}}$) is greater than 1.0. The assessment model ($N_{{{\text{GOI}}}}$) is evidently straightforward and practical. As $N_{{{\text{GOI}}}}$ is a dimensionless group model, it can be readily applied in multi-scale scenarios, including the flow physical simulation, the theoretical research or calculation, the numerical simulation, the pilot tests and the field applications. This makes it a highly promising and valuable tool for further promotion and application.

## Conclusion

The mechanism and technology of artificial CO_2_ gas cap immiscible rigid stable gas flooding has the feasibility of greatly EOR and large-scale CO_2_ geological storage. According to the research, the artificial CO_2_ gas cap immiscible rigid stable gas flooding process should be defined as a gas flooding mode in which an artificial CO_2_ gas cap with sufficient energy and large volume is formed after a large amount of CO_2_ is injected into the crest of the inclined reservoir, the production well uses the expansion energy of the artificial CO_2_ gas cap as the flooding force for production, while the gas injection well continues to artificially inject CO_2_ into the gas cap. Under this flooding mode, the energy supply of the reservoir is sufficient, the gas cut volume (or gas injection volume) fully compensates the fluid production, the injection-production ratio is equal to 1, and the gas displacing oil front remains stable until the oil in the reservoir is completely produced.

On the basis of depriving the critical rate model of artificial CO_2_ gas cap immiscible rigid stable gas flooding, a bran-new dimensionless group model or assessment model of artificial CO_2_ gas cap immiscible stable gas flooding number ($N_{{{\text{GOI}}}}$) was established, a new method for quickly estimating artificial CO_2_ gas cap immiscible stable gas flooding suitability in the oilfield with strata dip has been proposed, which give a theoretical foundations and technical guidance for Efficient CO_2_ EOR (Oil recovery could be as high as 90% or more), Large-scale CO_2_ Sequestration (CO_2_ storage volume may even be 5.8 times the volume of crude oil in the ground), evaluating the stability of gas flooding front, suitable reservoir screening and field construction. Compared with previous achievements and understandings, the assessment model ($N_{{{\text{GOI}}}}$) is a more comprehensive solution in the crestal immiscible stable gas flooding process so far. When $N_{{{\text{GOI}}}}$ is greater than 1, the artificial CO_2_ gas cap immiscible stable gas flooding mode can be achieved, and the effect of EOR and CO_2_ Sequestration is obvious, when $N_{{{\text{GOI}}}}$ is less than 1, the crestal gas injection is easy to gas breakthrough, which does not achieve the expected effect.

The mechanics behavior influencing the new dimensionless group model (i.e., the assessment model) include crude oil density, liquid (oil–gas mixture) phases relative permeability, air permeability in formation direction, crude oil viscosity, strata dip, injected gas density, gas injection rate (gas cut rate), injected gas viscosity, gas phase relative permeability, etc. According to the order of the above influencing factors, their influence degree on the stability of the gas displacing oil front and the development effect during the artificial CO_2_ gas cap immiscible rigid stable gas flooding process decreases successively. The research established a correlation between $N_{{{\text{GOI}}}}$ and several parameters, including crude oil density, liquid phases (oil–gas mixture), relative permeability at the gas–displacing oil front, air permeability in the formation direction, strata dip and injected gas viscosity. The findings indicated a positive linear relationship between these parameters and $N_{{{\text{GOI}}}}$, emphasising the significance of $N_{{{\text{GOI}}}}$ in understanding and interpreting the characteristics of the gas displacing oil under crestal gas injection. The injected gas density, crude oil viscosity, gas injection rate and gas phase relative permeability at the gas displacing oil front are all negatively correlated with $N_{{{\text{GOI}}}}$. Of these, the injected gas density has a linear relationship with $N_{{{\text{GOI}}}}$, while the crude oil viscosity, the gas injection rate and gas phase relative permeability have a power function relationship with $N_{{{\text{GOI}}}}$. The research argued that the buoyancy, the capillary pressure (the result of the combined action of liquid phase and gas phase driving pressure) make the largest contribution to the artificial CO_2_ gas cap immiscible rigid stable gas flooding process and play a leading role, while the gravity makes a small contribution to the artificial CO_2_ gas cap immiscible rigid stable gas flooding process and plays a secondary role. Among them, the contribution of driving pressure( the ratio of the oil phase viscosity to liquid phase additional resistance), which play a leading role in capillary pressure, to the artificial CO_2_ gas cap immiscible rigid stable gas flooding process is second only to buoyancy. The influence of buoyancy, capillary pressure, additional resistance, viscosity and gravity in ACGCIRSF process decreases in turn, and the effect of filtrational resistance such as buoyancy, capillary pressure, additional resistance and viscosity are conducive to maintaining the stability of the gas displacing oil front.

Study shows that the dimensionless group model ($N_{{{\text{GOI}}}}$) is more reliable, practical and effective than the existing dimensionless group models and critical rate models because of the comprehensive consideration of the influencing factors and the complete construction of the mechanical action mechanism in the process of artificial gas cap immiscible gas flooding or artificial gas cap immiscible stable gas flooding or artificial gas cap immiscible rigid stable gas flooding. More importantly, the model and algorithm not only can be used as a creterion to assess the stablity and efficiency of crestal gas injection for stable flooding such as artificial gas cap immiscible rigid stable gas flooding, artificial gas cap immiscible stable gas flooding, gas assisted gravity drainage, gravity assisted gas injection, and crestal gas injection for stable gravity flooding for theoretical investigation, numerical simulation, laboratory test and field trial project design or operation. Compared with the traditional processes of crestal gas injection for gas flooding, the technique of artificial CO_2_ gas cap immiscible rigid stable gas flooding which could not only greatly improve crude oil recovery but also realize CO_2_ geological storage on a large scale, shows excellent gas–oil interface control ability and sequestration potential in tectonic and carbonate oil reservoirs or abandoned oil reservoirs, including but not limited to fault block reservoirs, especially oil reservoirs with dipping strata in the later stage of extremely high water cut development, etc., providing a green and feasible new path for deep carbon sequestration. So this achievement is innovative because it reveals the transport and storage laws during the development of artificial CO_2_ gas cap immiscible stable gas flooding. The established dimensionless number ($N_{{{\text{GOI}}}}$) provides a comprehensive solution, from mechanism research to engineering practice, for the low-carbon development of oil and gas reservoirs under crestal gas injection. This research’s value extends far beyond theory and technology, providing a viable pathway for the oil and gas industry to balance ‘emissions reduction’ and ‘production’ by significantly improving crude oil recovery (on the energy supply side) and efficiently sequestering CO_2_ (on the carbon neutral side). In addition, the scientificity, the rationality, and the applicability of the proposed dimensionless group number ($N_{{{\text{GOI}}}}$) and the artificial CO_2_ gas cap immiscible rigid stable gas flooding process or the artificial gas cap immiscible stable gas flooding process have been verified only by theoretical demonstration and a limited range of field and experiment tests. Therefore, more field tests and more laboratory experiments are needed to fully validate the new dimensionless group, after testing or considering the each phase permeability and the range of its variables. The analysis of influencing factors and their sensitivity must be based on the actual variation range of the reservoir parameters; otherwise, the conclusion will have no physical significance. It is anticipated that, on the basis of the present study, further physical–mechanical mechanism studies will be conducted in the future. The combination of microscopic visualisation experiments and simulations has the potential to reveal further information about the mechanisms in question. Such a combination would also facilitate the prediction of cross-scale CO_2_ transport and storage patterns, ranging from nano- to pore- to core-scale. The result of such a combination would be enhanced geological adaptability and engineering application value of the dimensionless number ($N_{{{\text{GOI}}}}$).

## Data Availability

No datasets were generated or analysed during the current study.

## List of symbols

$$\alpha$$: Strata dip, °
$\beta$: Interface angle between the gas–oil flooding front interface and the horizontal plane, °
$S_{{{\text{gp}}}}$: Volume of oil displaced by artificial CO_2_ gas cap gas in pores per unit volume, dimensionless
$S_{{{\text{wi}}}}$: Volume of bound water in pores per unit volume, dimensionless
$S_{{{\text{or}}}}$: Volume of residual oil in pores per unit volume, dimensionless
$f_{{\text{g}}}$: Buoyancy of artificial gas cap gas in pores per unit volume, N
$S_{{{\text{wp}}}}$: Volume of oil discharge by artificial CO_2_ gas cap gas in pores per unit volume, dimensionless
$\rho_{{\text{o}}}$: Oil density in reservoir, g/cm^3^
$\rho_{{\text{g}}}$: Gas density in artificial CO_2_ gas cap, g/cm^3^
$$g$$: Gravitational acceleration, 9.8 m/s^2^
$G_{{\text{g}}}$: Gravity of artificial CO_2_ gas cap gas in pores per unit volume, N
$F_{{\text{g}}}$: Difference between buoyancy and gravity of artificial CO_2_ gas cap gas in pores per unit volume,
$\frac{{\partial F_{{\text{g}}} }}{\partial h}$: Gradient of difference between buoyancy and gravity in vertical direction, Pa/m
$\frac{{\partial F_{{\text{g}}} }}{\partial x}$: Gradient of difference between buoyancy and gravity at stratigraphic direction, Pa/m
$G_{{\text{o}}}$: Gravity of artificial reservoir oil, N
$\frac{{\partial G_{{\text{o}}} }}{\partial h}$: Gradient of gravity of reservoir oil at vertical direction, Pa/m
$\frac{{\partial G_{{\text{o}}} }}{\partial x}$: Gradient of gravity of reservoir oil at stratigraphic direction, Pa/m
$v_{{{\text{g}}\angle }}$: Darcy flow rate of gas at stratigraphic direction, m/s
$v_{{{\text{o}}\angle }}$: Darcy flow rate of reservoir oil at stratigraphic direction, m/s
$k_{\angle }$: Air permeability of rock at the stratigraphic direction, mD
$K_{{{\text{rg}}\angle }}$: Gas phase relative permeability at the stratigraphic direction, dimensionless
$K_{{{\text{rog}}\angle }}$: Liquid phase relative permeability at the stratigraphic direction, dimensionless
$\mu_{{\text{g}}}$: Gas viscosity of artificial CO_2_ gas cap, mPa s
$\mu_{{\text{o}}}$: Formation crude oil viscosity, mPa s
$\frac{{\partial p_{{{\text{c}}\left( {\text{g-w}} \right)S_{{{\text{wi}}}} }} }}{\partial x}$: Capillary force gradient of gas–water, Pa/m
$\frac{{\partial p_{{{\text{c}}\left( {\text{o-w}} \right)S_{{{\text{wi}}}} }} }}{\partial x}$: Capillary force gradient of oil–water, Pa/m
$\frac{{\partial p_{{{\text{c}}\left( {\text{g-o}} \right)}} }}{\partial x}$: Capillary force gradient of gas–oil, Pa/m
$\frac{{\partial p_{{\text{g}}} }}{\partial x}$: Pressure gradient of gas phase, Pa/m
$\frac{{\partial p_{{\text{o}}} }}{\partial x}$: Pressure gradient of oil phase, Pa/m
$v_{t}$: Darcy flow rate of gas, m/s
$p_{{{\text{c}}\left( {\text{g-o}} \right)}}$: Capillary of gas–oil in stratigraphic direction, Pa
$h$: Height of rise of the non-wetting phase in the capillary tube, m
$v_{{{\text{tc}}}}$: Darcy flow rate or rate of artificial CO_2_ gas cap gas cut, m/s
${N}_{\text{GOI}}$: Dimensionless group of the stability of artificial gas cap flooding, dimensionless
$$\phi$$: Porosity, dimensionless
$S_{{{\text{AF}}}}$: Sensitivity coefficient which represents the ratio of the percentage change in project analysis indicators to the percentage change in uncertain factors and is derived from the Engineering Economics, reflecting the sensitivity of evaluation indicator $A$ to the uncertain influencing factor $F$, dimensionless
$\Delta F/F$: Change rate of influencing factor, dimensionless
$\Delta A/A$: Change rate of the corresponding evaluation index value, dimensionless
$\rho_{{\text{o}}} g{\text{sin}}\alpha$: Gravity gradient per unit volume of crude oil at stratigraphic direction, Pa/m
$\left( {\rho_{{\text{o}}} - \rho_{{\text{g}}} } \right)g{\text{sin}}\alpha$: Gradient of difference between buoyancy and gravity, Pa/m
$\frac{{\mu_{{\text{o}}} v_{{\text{t}}} }}{{k_{\angle } K_{{{\text{rog}}\angle }} }}$: Driving pressure gradient of crude oil at stratigraphic direction, Pa/m
$\frac{{\mu_{{\text{g}}} v_{{\text{t}}} }}{{k_{\angle } K_{{{\text{rg}}\angle }} }}$: Driving pressure gradient of gas phase, Pa/m
$\frac{\partial p}{{\partial x}}$: Driving pressure gradient, Pa/m
$N^{\prime}_{{\text{B}}}$: New Bond number, dimensionless
${\text{Re}}$: Reynolds number, dimensionless
$v$: Darcy flow or injection rate, m/s
$$\rho$$: Density, g/cm^3^
$l$: Length of the core, m
$\mu$: Viscosity of the displacing phase, Pa s
${\text{Pe}}$: Peclet number, dimensionless
$$\lambda$$: Thermal conductivity, J/m s K
$c$: Heat capacity per unit mass, J/kg K
${\text{Sc}}$: Schmid number, dimensionless
$$D$$: Diffusion constant, m^2^/s
${\text{We}}$: Weber number, dimensionless
$$\sigma$$: Interfacial tension, N/m
${\uptheta }$: Wetting angle of fluid interface, dimensionless
${\text{Fr}}$: Froude number, dimensionless
$$g$$: Gravitational acceleration, 9.8 m/s^2^
${\text{Pr}}$: Prandtl number, dimensionless
${\text{Le}}$: Lewis number, dimensionless
$\beta$: Thermal cubic expansion coefficient, K^−^^1^
$$T$$: Temperature, K
${\text{Gr}}$: Grashof number, dimensionless
$I.P.$: Index of productivity, Bbl/D-Ac
$N_{{\text{G}}}$: Gravity number, dimensionless
$\Delta \rho$: Density difference between the displaced phase and the displacing phase, g/cm^3^
$K$: Absolute permeability of the porous media, mD
$$\phi$$: Porosity, dimensionless
$\mu_{{\text{o}}}$: Oil phase viscosity, Pa s
$v_{{\text{d}}}$: Oil phase rate, m/s
$k$: Reservoir permeability, mD
$\Delta \mu$: Viscosity difference between the displaced and displacing phase, Pa s
$\mu_{{\text{g}}}$: Gas viscosity, Pa s
$v_{{\text{g}}}$: Gas Darcy rate, m/s
$N^{\prime}_{{\text{G}}}$: Modified gravity number, dimensionless
$N_{{{\text{GD}}}}$: Gravity drainage number, dimensionless
$\rho_{{\text{g}}}$: Formation gas density, g/cm^3^
$\rho_{{\text{o}}}$: Formation crude oil density, g/cm^3^
$N_{{\text{C}}}$: Capillary number, dimensionless
$N_{{\text{B}}}$: Bond number, dimensionless
$N^{\prime}_{{{\text{GD}}}}$: Modified gravity number, dimensionless
$N_{{\text{G, Y}}}$: Yang gravity number, dimensionless
$\Delta \rho_{o,g}$: Density difference between oil phase and gas phase, g/cm^3^
$$\alpha$$: Strata dip, °
$N_{{\text{C,1}}}$: Capillary number, dimensionless
$N_{{\text{C,2}}}$: Capillary number, dimensionless
$k_{{\text{w}}}$: Specific permeabilities of the sample to the aqueous phase, mD
$k_{{\text{a}}}$: Specific permeabilities of the sample to the air phase, mD
$\frac{\Delta P}{L}$: Imposed pressure gradient across the sample of length $L$, Pa/m
$N_{{\text{C,3}}}$: Capillary number, dimensionless
$N_{{{\text{ca}}}}$: Capillary number, dimensionless
$N_{{\text{C}}}$: Three-phase Capillary number, dimensionless
$P{\text{c}}_{{\text{i}}}$: Controlling density difference in capillary force specially defined for each case, Pa
$$P{\text{c}}$$: Capillary, Pa
$R_{{\text{a}}}$: Average pore throat radius, m
$H$: Thickness of reservoir, m
$K_{{{\text{ro}}}}$: Oil phase permeability, mD
$N_{{{\text{Cam}}}}$: Yang variation Capillary number, dimensionless
$S_{{\text{o}}}$: Oil saturation, dimensionless
$S_{{{\text{or}}}}$: Residual oil saturation, dimensionless
$N_{{\text{B}}}$: Macroscopic Bond number, dimensionless
$r$: Sphere radius, μm
$R$: Particle radius, μm
$h$: Height of the ganglion, m
$r_{{{\text{av}}}}$: Average pore throat radius of the porous medium, m
$h_{{{\text{av}}}}$: Average height of the non-wetting phase ganglia, m
$$L$$: Sample length, m
$P_{{\text{c}}}^{*}$: Characteristic capillary, taken as the critical entry pressure for gas, Pa
$Z$: Average position of the gas interface, m
$\sigma_{{_{og} }}$: Interfacial tension between oil and gas, N/m
$\Delta \rho_{{\text{i}}}$: Controlling density difference in capillary specially defined for each case, g/cm^3^
$\sigma_{{\text{i}}}$: Controlling interfacial tension in capillary specially defined for each case, N/m
$N_{{{\text{DB}}}}$: Dombrowski–Brownell number, dimensionless
$\tilde{N}_{{{\text{DB}}}}$: Modified Dombrowski–Brownell number, dimensionless
$k_{r}$: Relative permeability, dimensionless
$N^{\prime}_{{\text{b}}}$: Chen Bond number, dimensionless
$A$: Scaling constant, dimensionless
$\mu_{{\text{d}}}$: Displaced phase viscosity, Pa s
$N_{{{\text{co}}}}$: Rostami number, dimensionless
$N_{{\text{a}}}$: Yang comprehensive number, dimensionless
$N_{{{\text{dis}}}}$: Dissolution number, dimensionless
$N_{{{\text{D}}1}}$: Diffusion number, dimensionless
$N_{Pe}$: Peclet’s number, dimensionless
$N_{{{\text{D}}2}}$: Dispersion number, dimensionless
$N_{{u_{c} }}$: Dietz number, dimensionless
$u_{{\text{c}}}$: Gas injection rate, m/s
$N_{{\left( {{q \mathord{\left/ {\vphantom {q A}} \right. \kern-0pt} A}} \right)_{{{\text{st}}}} }}$: Rutherford number, dimensionless
$\mu_{{\text{s}}}$: Viscosity of the displacing fluids, Pa s
$\rho_{{\text{s}}}$: Density of the displacing fluids, g/cm^3^
$\left( {{q \mathord{\left/ {\vphantom {q A}} \right. \kern-0pt} A}} \right)_{{{\text{st}}}}$: Maximum flow rate for a stable displacement, m/s
$N_{{u_{{{\text{st}}}} }}$: Dumore number, dimensionless
$u_{{{\text{st}}}}$: Critical rate for stable displacement, m/s
$N_{{{\text{S}}u_{c} }}$: Slobod and Howlett number, dimensionless
$N_{{V_{{\text{c}}} }}$: Hill number, dimensionless
$V_{{\text{c}}}$: Critical rate of flow, m/s

## References

[1] Geertsma, J., Croes, G. A. & Schwarz, N. Theory of dimensionally scaled models of petroleum reservoirs. In *SPE-539-G*, vol. 207, 118–127 (1956). 10.2118/539-G

[2] Kulkarni, M. M. *Multiphase Mechanisms and Fluid Dynamics in Gas Injection Enhanced Oil Recovery Processes* Doctor thesis, Louisiana State University and Agricultural and Mechanical College (2005).

[3] Kulkarni, M. M. & Rao, D. N. In *2006 SPE Annual Technical Conference and Exhibition* San Antonio, Texas, USA (2006).

[4] Shook, M., Li, D. & Lake, L. Scaling immiscible flow through permeable media by inspectional analysis. *Situ* **16**, 311–349 (1992).

[5] Sharma, A. & Rao, D. N. In *SPE Symposium on Improved Oil Recovery*, vol. 23 (Society of Petroleum Engineers, Tulsa, Oklahoma, USA, 2008).

[6] Wu, K. et al. Prediction method of oil recovery in gas–assised gravity drainage process. *v* **19**, 61–65 (2012).

[7] Chatzis, I. & Morrow, N. R. Correlation of Capillary number relationships for sandstone. In *SPE-10114-PA*, vol. 24, 555–562. 10.2118/10114-PA (1984).

[8] Yang, C., Li, Y., Han, J. & Xu, X. Quantitative evaluation and screening method for gas assisted gravity drainage reservoirs. *Acta Pet. Sin.* **34**, 938–946. 10.7623/syxb201305015 (2013).

[9] Ren, S. et al. Gravity assisted gas injection: Assessment model and experimental study. *J. China Univ. Pet.*. **42**, 59–66 (2018).

[10] Morrow, N. R., Chatzis, I. & Taber, J. J. Entrapment and mobilization of residual oil in bead packs. In *SPE-14423-PA 3* 927–934 10.2118/14423-PA (1988).

[11] Grattoni, C., Jing, X. & Dawe, R. Dimensionless groups for three-phase gravity drainage flow in porous media. *J. Pet. Sci. Eng.* **29**, 53–65. 10.1016/S0920-4105(00)00090-5 (2001).

[12] Hove, A. O., Dawe, R. A. & Evans, R. N. Gravity segregation at the pore scale in cores under miscible and low interfacial tension conditions including in-situ tomography. *J. Pet. Sci. Eng.* **14**, 89–98. 10.1016/0920-4105(95)00037-2 (1995).

[13] Edwards, J. et al. New Orleans, Louisiana. In *1998 SPE Annual Technical Conference and Exhibition* (1998).

[14] Chen, X., Li, Y., Guan, C. & Chen, C. An optimized neural network prediction model for gas assisted gravity drainage recovery based on dimensional analysis. *Pet. Sci. Bull.* **4**, 288–299. 10.3969/j.issn.2096-1693.2019.03.026 (2019).

[15] Rostami, B., Kharrat, R., Pooladi-Darvish, M. & Ghotbi, C. Identification of fluid dynamics in forced gravity drainage using dimensionless groups. *Transp. Porous Media*. **83**, 725–740 (2010).

[16] Rostami, B. et al. A new approach to characterize the performance of heavy oil recovery due to various gas injection. *Int. J. Multiph. Flow*. **99**, 273–283 (2018).

[17] Kelkar, B. G. & Gupta, S. P. In *SPE Enhanced Oil Recovery Symposium*, vol 12 (Society of Petroleum Engineers, Tulsa, Oklahoma, 1988).

[18] Li, J. & Liu, B. Influence of gas injection parameters on the effect of unstable-state displacement by gas flooding. *J. Oil Gas Technol.* **31**, 130–134 (2009).

[19] Novakovic, D. *Numerical Reservoir Characterization Using Dimensionless Scale Numbers with Application in Upscaling* (2002).

[20] N Dietz, D. A theoretical approach to the problem of encroaching and by-passing edge water. *Proc. Koninklijke Nederlandse Akademie Van Wetenschappen* **56-B**, 83–92 (1953).

[21] RutherfordW. M. Miscibility relationships in the displacement of oil by light hydrocarbons. *SPE-10114-PA* **2** (340-346). 10.2118/449-PA (1962).

[22] Dumore, J. M. Stability considerations in downward miscible displacements. In *SPE-10114-PA*, vol. 4, 356–362 (1964). 10.2118/961-PA

[23] Slobod, R. L. & Howlett, W. E. The effects of gravity segregation in laboratory studies of miscible displacement in vertical unconsolidated porous media. In *SPE-10114-PA*, vol. 4, 1–8 (1964). 10.2118/743-PA

[24] Hill, D. G. In *SPE Formation Damage Control Symposium*, vol.12 (Society of Petroleum Engineers, Lafayette, Louisiana, 1982).

[25] Guo, P., Zhang, C. & Xiong, J. Present state of gas–assisted gravity Drainge. *Sci. Technol. Eng.* **15**, 176–184 (2015).

[26] Wang, R. Interface stability conditions and influencing factors of artificial gas cap flooding. *Sci. Technol. Eng.* **21** (2021).

[27] Gao, Z., Liu, Z. & Du, X. *Gas Injection Recovery Technology in Oil Field* 73–85 (Petroleum Industry, 1994).

[28] Sandrea, R. & Nielsen, R. F. Dynamics of petroleum reservoirs under gas injection (1974).

[29] Kong, X. *Advanced Seepage Mechanics*, 3 edn, 312–357 (University of Science and Technology of China, 2020).

[30] Gunawan, S. & Caie, D. Handil field: Three years of lean-gas injection into waterflooded reservoirs. *SPE Reserv. Eval. Eng.* **4**, 107–113. 10.2118/71279-PA (2001).

[31] Gillham, T., Cerveny, B. & Turek, E. *West Hackberry Tertiary Project*. Annual report, September 3, 1994–September 2, 1995, 1-225 (Amoco Production Co., Houston, TX, United States, 1996).

[32] Gillham, T. H. *West Hackberry Tertiary Project. Report No. DOE/BC/14963-19* (National Petroleum Technology Office, Tulsa, OK (US), United States, 1999).

[33] Rao, D. N. et al. *Development and Optimization of gas–Assisted Gravity Drainage (GAGD) Process for Improved Light Oil Recovery* (Louisiana State Univ., Baton Rouge, LA (United States), (2006).

[34] King, R. L. & Lee, W. J. An Engineering study of the Hawkins (Woodbine) field. In *SPE-5528-PA*, vol. 28, 123–128 (1976). 10.2118/5528-PA

[35] Carlson, L. O. In *SPE Enhanced Oil Recovery Symposium* (OnePetro, Tulsa, Oklahoma, 1988).

[36] Bai, F., Sheng, Y., Meng, Q., Song, F. & Weng, Y. Reservoir engineering research of the nitrogen injection pilot in Yanling oilfield. *Acta Pet. Sin.* **19**, 61–68 (1998).

[37] Xu, K. & Xu, N. The nitrogen injection technology in Yanling Oilfield. Oil drilling & production technology. *Oil Drill. Prod. Technol.* **20**, 69–75 (1998).

[38] Huang, D. Laboratory evaluation of the mechanism of the increased oil recovery with nitrogen injection for Yanling oilfield. *Acta Pet. Sin.* **13**, 67–75 (1992).

[39] Yu, Z. The case on the development of fractured carbonate pool(1)—Yanling oil field. *Pet. Explor. Dev.* **5**, 45–51 (1987).

[40] Wang, J. & Shen, Z. Give Yanling oil field adjustment in a word. *Oil Drill. Prod. Technol.* **3**, 41–44. 10.13639/j.odpt.1981.01.006 (1981).

[41] Bai, S. & Tang, F. *The Developent Models of Buried Hill Fractured Basement Reservoirs* (Petroleum industry, 1997).

[42] Welge, H. J. A simplified method for computing oil recovery by gas or water drive. In *SPE-5528-PA*, vol. 4, 91–98 (1952).

[43] Leverett, M. C. Capillary behavior in porous solids. In *SPE-539-G*, vol. 142, 152–169 (1941). 10.2118/941152-g

[44] Buckley, S. E. & Leverett, M. Mechanism of fluid displacement in sands. In *SPE-539-G*, vol. 146, 107–116 (1942).

[45] Xiong, D., Sun, L., Zhao, X., Zhang, Y. & Dong, G. The assessment on the economic feasibility of gas injecting plan for deep condensate oil and gas reservoir. *J. Xi’an Shiyou Univ. (Social Sci. Edn.)* **3**, 5–11 (2010).

[46] Zhu, Y. *Economic Evaluation on Proved Geological Reserves in K Oilfield K3 Fault Blocks. College of Business Administration*. Master thesis, Southwest Petroleum University (2018).

[47] huang, Y. *Petroleum Technical Economics* (China University of Geosciences, 2014).

[48] Rao, D. N., Ayirala, S. C., Kulkarni, M. M. & Sharma, A. P. In *SPE/DOE Symposium on Improved Oil Recovery*, vol. 12 (Society of Petroleum Engineers, Tulsa, Oklahoma, 2004).

[49] Pang, J. *Mechanism Studies of Crestal Gas Injection for Gravity Stable Flooding to Enhance Oil Recovery* Master thesis, Southwest Petroleum University (2006).

[50] Liang, S., Zhou, W. & Zhang, J. Investigation on effect application of the technology of crestal gas injection for stable gravity flooding. *J. Southwest. Pet. Univ. (Sci. Technol. Edn.)* **36**, 86–92. 10.11885/j.issn.1674-5086.2013.09.02.01 (2014).

[51] Saikia, B. D. & Rao, D. N. gas–assisted gravity drainage—a new process for CO_2_ sequestration and enhanced oil recovery. In *TechConnect Briefs*, vol 2, Materials for Energy, Efficiency and Sustainability: TechConnect Briefs 191–194 (2015).

[52] Zhou, W., Zhang, J., Tang, Y., Chai, X. & Yan, Z. Application of top gas Injection-Assisted gravity drainage in bottom water reservoirs. *J. Southwest. Pet. Univ. (Sci. Technol. Edn.)* **39**, 92–100. 10.11885/j.issn.1674-5086.2016.05.10.03 (2017).

[53] Diwu, P., Sun, C., Liu, R. & Zhao, W. Artificial gas cap flooding technology of complex fault block reservoir. *Adv. Mater. Res.* **1073–1076**, 2272–2275. 10.4028/www.scientific.net/AMR.1073-1076.2272 (2015).

[54] Chang, Y. et al. The study on crestal injection for fault block reservoir with high dip and low permeability. *Sci. Technol. Eng.* **16**, 179–183 (2016).

[55] Liu, W., Wu, Y., Li, W. & Sun, N. Screening criteria of gas cap-edge water drive fault block oil reservoir. *Special Oil Gas Reserv.* **23**, 104–108 + 156. 10.3969/j.issn.1006-6535.2016.01.023 (2016).

[56] Xu, B. *Research on Mechanism and Numerical Simulation of Artificial Gas Cap Displacement of Oil* Master thesis, China University of Geosciences (Beijing) (2017).

[57] Zhao, Y. *Research on Technology Policy of Fault Block Oil Reservoir Artificial Gas Cap Displacement of Oil* Master thesis, China University Of Petroleum(Beijing), (2018).

[58] Liu, W. Evaluation theory method and parameter boundary for the formation of artificial gas cap. *Oil Drill. Prod. Technol.* **42**, 207–213. 0.13639/j.odpt.2020.02.014 (2020).

[59] Yu, S. *The Development Models of Complicated Fault-Block Sandstone Oilfield* (Peroleum Industry, 1998).

[60] Jiang, Y., Liu, H., Zhang, Z., Wang, D. & Wang, Y. Research of reservoirs injecting strategy for different gas cap index and its application. *Well Test.* **26**, 25–27 (2017).

[61] Fang, N., Liu, Z., Lv, Z., Cheng, D. & Wen, J. Gas channeling pattern and full-life development strategy for oil reservoir with large gas cap. *Special Oil Gas Reserv.* **25**, 117–121. 10.3969/j.issn.1006-6535.2018.03.023 (2018).

[62] Feng, Y. *The Research on Development Technology and Countermeasures of JZ25-1S Gas Cap Reservoir with Side Water* Master thesis, China University of Petroleum(Beijing) (2017).

[63] Cao, H., Zhang, X. & Chen, L. Efficient development and production maintenance measures of reservoir with little oilring and large gas–cap. *Special Oil Gas Reserv.* **23**, 97–101. 10.3969/j.issn.1006-6535.2016.03.023(2016).

[64] Haley, K. A., Gillham, T. H. & Yannimaras, D. *West Hackberry Tertiary Project, Class. Report No. DOE/BC/14963-21*, vol. 36 (Amoco Production Company, Houston, Texas, 2002).

[65] Wang, D. *Appicadion of Nirogen and Fue Gas To Oil and Gas Field Development* (Petroleum Industry, 1991).

[66] Rivas, O., Embid, S. & Bolívar, F. Ranking reservoirs for carbon dioxide flooding processes. *SPE Adv. Technol. Ser.* **2**, 95–103. 10.2118/23641-pa (1994).

[67] Diaz, D., Bassiouni, Z., Kimbrell, W. & Wolcott, J. In *SPE Improved Oil Recovery Conference?* SPE-35431-MS (SPE).

[68] Lepski, B., Bassiouni, Z. & Wolcott, J. In *SPE Improved Oil Recovery Conference?* SPE-39659-MS (SPE).

[69] Pang, Y., Guo, H., Yang, Z. & Luo, Z. *Developing Instance of Gas Injection in Overseas Oilfelds* (Peroleum Industry, 2001).

